# The role of glycosyltransferase enzyme GCNT3 in colon and ovarian cancer prognosis and chemoresistance

**DOI:** 10.1038/s41598-018-26468-4

**Published:** 2018-05-31

**Authors:** Lara P. Fernández, Ruth Sánchez-Martínez, Teodoro Vargas, Jesús Herranz, Roberto Martín-Hernández, Marta Mendiola, David Hardisson, Guillermo Reglero, Jaime Feliu, Andrés Redondo, Ana Ramírez de Molina

**Affiliations:** 1 Molecular Oncology Group, IMDEA Food Institute, CEI UAM + CSIC, Madrid, Spain; 2 Biostatistics and Bioinformatics Unit, IMDEA-Food Institute, CEI UAM + CSIC, Madrid, Spain; 3 Molecular Pathology Section, Institute of Medical and Molecular Genetics (INGEMM) La Paz University Hospital, Madrid, Spain; 4 Molecular Pathology and Therapeutic Targets Lab, IdiPAZ, La Paz University Hospital, Madrid, Spain; 5 Pathology Department, IdiPAZ, La Paz University Hospital, Madrid, Spain; 6 Translational Oncology Lab, IdiPAZ, La Paz University Hospital, Madrid, Spain; 7 Clinical Oncology Department, La Paz University Hospital, Madrid, Spain; 8 CIBERONC CB16/12/00398, La Paz University Hospital, Madrid, Spain; 9 Catedra UAM-AMGEN, La Paz University Hospital, Madrid, Spain

## Abstract

Glycosyltransferase enzyme GCNT3, has been proposed as a biomarker for prognosis in colorectal cancer (CRC). Our study goes in depth into the molecular basis of GCNT3 role in tumorigenesis and drug resistance, and it explores its potential role as biomarker in epithelial ovarian cancer (EOC). High levels of *GCNT3* are associated with increased sensibility to 5-fluoracil in metastatic cells. Accordingly, GCNT3 re-expression leads to the gain of anti-carcinogenic cellular properties by reducing cell growth, invasion and by changing metabolic capacities. Integrated transcriptomic and proteomic analyses reveal that GCNT3 is linked to cellular cycle, mitosis and proliferation, response to drugs and metabolism pathways. The vascular epithelial growth factor A (VEGFA) arises as an attractive partner of GCNT3 functions in cell invasion and resistance. Finally, GCNT3 expression was analyzed in a cohort of 56 EOC patients followed by a meta-analysis of more than one thousand patients. This study reveals that GCNT3 might constitute a prognostic factor also in EOC, since its overexpression is associated with better clinical outcome and response to initial therapy. GCNT3 emerges as an essential glycosylation-related molecule in CRC and EOC progression, with potential interest as a predictive biomarker of response to chemotherapy.

## Introduction

Despite major advances in our understanding of cancer, resistance to chemotherapy is an ongoing challenge. The mechanisms of resistance are due in part, to alterations in the pattern of mucins expression^1^. Mucins are high-molecular-weight O-glycoproteins that create a mucosal protection system at the surface of the gastrointestinal tract. In the tumour local environment, a modified expression of mucins could form an improper network that makes target sites inaccessible to drugs^2,3^. The structural and functional characteristics of mucins are mainly settle by their carbohydrate moieties which are synthesized among others, by glycosyltransferases enzymes^4^. Due to the frequent alteration of the pattern of mucins and glycosyltransferases expression in cancers^5–8^ as well as their molecular characteristics, glycosyltransferases are thought to also be involved in drug response^1,3,9,10^.

The mucin-type core 2 1,6-N-acetylglucosaminyltransferase enzyme (C2GnT-M), encoded by the *GCNT3* gene, is a glycosyltransferase enzyme whose expression is altered in cancer processes^10–13^. GCNT3 catalyzes the formation of core 2 O-glycan, core 4 O-glycan and I branches^14^ and its pattern of expression has been mainly associated with colorectal cancer (CRC) prognosis^11,13,15–17^. *GCNT3* gene expression has been found down-regulated in CRC samples in comparison to non-pathological colon tissue^11,13,15^. Moreover, *GCNT3* transfection in certain CRC cells seemed to reduce cell proliferation, adhesion, invasion, and induced cell death, and also inhibited tumor growth *in vivo*^11^. Previous studies of our group suggested that GCNT3 could be a potential marker for good prognosis in CRC and it might also be a biomarker to monitor tumor response to chemotherapy in these patients^13^.

In cancers of sporadic origin, the majority of deaths are frequently due to diagnosis at an advanced stage. Like in CRC, up to 90% of epithelial ovarian cancers (EOC) might be cured if identified at an early stage^18^. Both, are examples of tumors that may present widespread disease without an obvious primary site^19^. Detection of EOC is usually delayed because of a lack of clear symptoms and an absence of ideal biomarkers^20^. Among EOC, serous carcinoma is the most common subtype and has the poorest prognosis with a five-year survival rate of 10–20%^21^. Frequently, EOC are diagnosed at stage III or IV and poor overall survival rates are due to the rapid metastasis, drug resistance and high recurrence possibility^20^.

In order to further investigate the role of GCNT3 as a biomarker for cancer patients, here, we took an integrated analysis of GCNT3 landscape of interactors and regulators. The results obtained in this study provide us new insights into GCNT3 transcriptional and proteomic networks, and confirm the involvement of GCNT3 in relevant biological processes and pathways related to cancer and drug resistance. Besides CRC, we expand the clinical use of GCNT3 as a new biomarker tool by introducing its potential applicability for EOC patients.

## Results

### GCNT3 overexpression contributes to reduce 5-FU resistance in metastatic CRC cells

**Figure 1:**
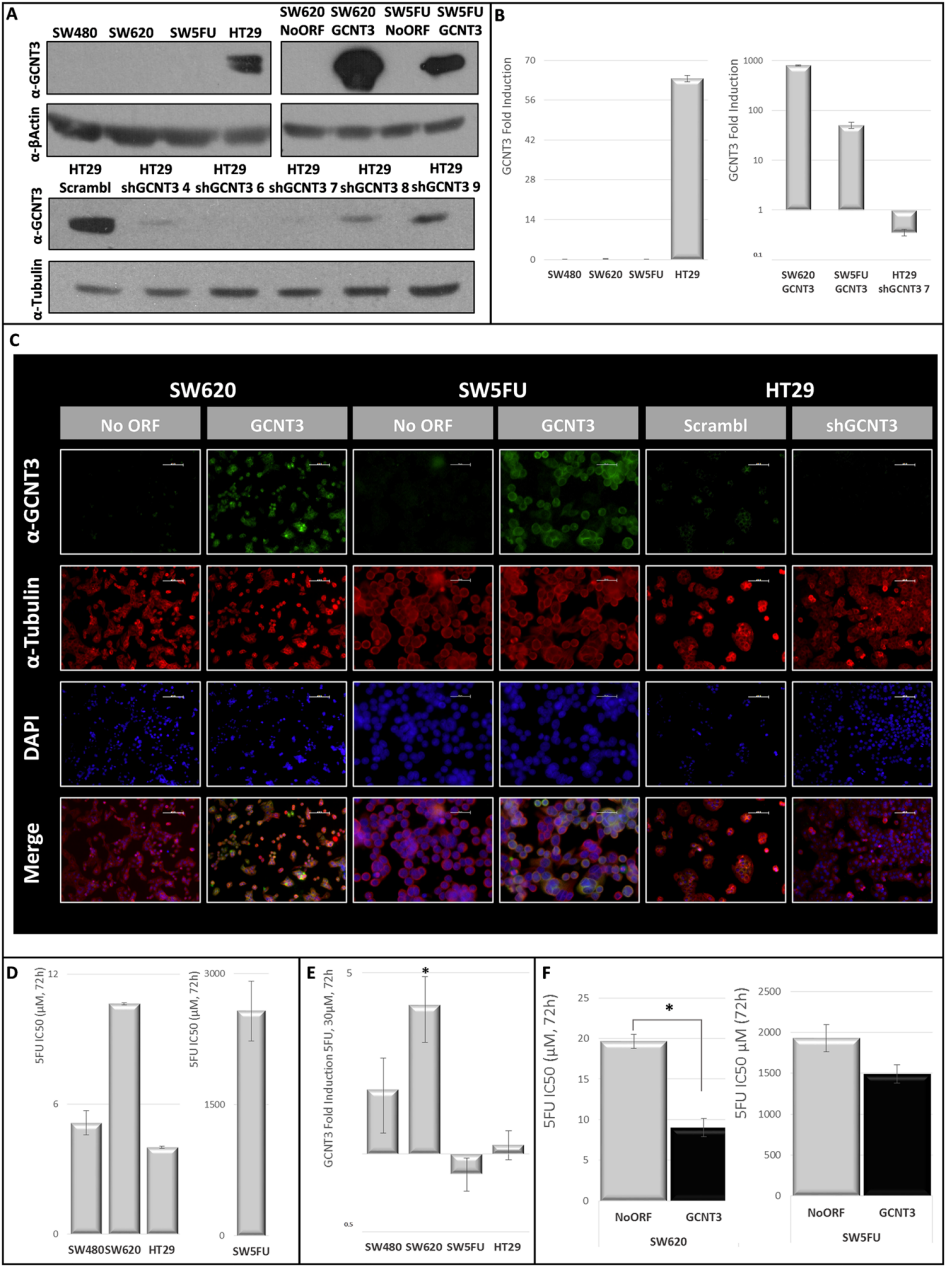
GCNT3 overexpression reduces 5FU resistance in CRC cells. (Panel A) Protein expression levels of GCNT3 in non-infected colorectal cancer (CRC) cells, stable cell lines overexpressing GCNT3 and a battery of shGCNT3. Proteins were detected by western blot using specific antibodies against GCNT3, β-Actin and β-Tubulin, as a loading control. Full-length blots/gels are presented in Supplementary Fig. 2. (Panel B) mRNA expression levels of GCNT3 measured by RT-QPCR, in non-infected CRC cells, stable cell lines overexpressing GCNT3 and shGCNT3 number 7. Data represent mean ± SEM of three independent experiments. (Panel C) Representative immunofluorescence images of GCNT3 (green) and Tubulin (red) of NoORF, GCNT3, Scrambl and shGCNT3 7 cells. Nuclei were stained with DAPI (blue). Scale bars 50 µm. (Panel D) Comparison of 5-fluoracil (5FU) IC50 values (concentration needed for 50% of viability inhibition) between non-infected CRC cells. Cell viability assays were performed after 72 h treatment. Data represent mean ± SEM of at least two independent experiments each performed in triplicate. (Panel E) Induction of GCNT3 expression by 5FU in CRC cells. Tumour cells were treated with 30 µM 5FU, during 72 h, and their mRNA GCNT3 expression was measured by RT-QPCR and represented in comparison to controls (vehicle-treated cells). Results are expressed as the mean ± SEM. of three independent experiments, each performed in triplicate. Student’s t test was applied to assess statistically significant differences (*p < 0.05). (Panel F) Comparison of 5FU IC50 values between NoORF and GCNT3 cells. Cell viability assays were performed after 72 h treatment. Data represent mean ± SEM of at least three independent experiments each performed in triplicate. Asterisk indicates statistically different values in GCNT3 cells respect to the control (NoORF cells), (*p < 0.05).

In order to investigate the role of GCNT3 as cancer prognostic factor as well as its relationship with drug resistant, we analyzed mRNA and protein expression levels of *GCNT3* in a panel of several CRC cell lines. Only the non-invasive HT29 cell line, that was isolated from a primary tumor, showed GCNT3 expression (mRNA and protein). By contrast, cells belonging to metastatic and invasive SW family did not exhibit measurable GCNT3 expression (Fig. 1. Panels A and B). To characterize *in vitro* GCNT3 effects, we generated CRC cell models of *GCNT3* overexpression and inhibition. We stably overexpressed *GCNT3* gene in SW620 and SW5FU cell lines, as we were interested in invasiveness and drug resistance. The overexpression of *GCNT3* in both cellular types was demonstrated by western blot and immunofluorescence (protein), and by qPCR (mRNA) (Fig. 1. Panels A, B and C). Besides, we inhibited *GCNT3* expression in HT29 cells. We tested activity of several shRNAs targeting GCNT3 (shGCNT3s) as it is shown in Fig. 1. Panel A. We found that shGCNT3 7 had the best inhibitory capacity (protein and mRNA) (Fig. 1. Panels A, B and C).

With the aim of studying GCNT3 relation with drug resistance, we determined the anti-proliferative activity of 5FU in our CRC cell lines panel (Fig. 1. Panel D). As a quantitative measure of this issue, we calculated IC50 parameter (concentration corresponding to 50% viability inhibition) of 5FU (0–5000 µM) in non-infected CRC cells. Unsurprisingly, SW5FU resistant cells were almost not sensitive to 5FU action and they showed the highest IC50 value (2573.2 ± 342.3 µM, mean ± SEM). Among 5FU non-resistant cells, SW620 line was the less sensitive to the drug action (IC50: 10.62 ± 0.04 µM).

Next, we examined the modulation of *GCNT3* expression in the presence of 5FU (30 µM). We observed a robust induction of *GCNT3* expression in SW family of non-resistant cells with a statistically significant fold-increase of 3.76 in SW620 metastatic cells. As expected, we did not observe such induction in SW5FU resistant cells or in HT29 cell line, which has the highest levels of endogenous GCNT3 (Fig. 1. Panel E).

To better evaluate the putative role of GCNT3 in drug resistance, we analyzed the anti-proliferative effect of 5FU in our GCNT3 overexpression models. We observed a 54.2% of reduction in the 5FU concentration needed for 50% of viability inhibition (IC50), in GCNT3 SW620 cells (NoORF IC50: 19.6 ± 0.84 *vs* GCNT3 IC50: 9.01 ± 1.11, *t-Student p-value* = 0.01) (Fig. 1. Panel F). Besides, we are also able to detect an IC50 reduction (22.8%) in GCNT3 SW5FU resistant cells (NoORF IC50: 1928.9 ± 167.5 *vs* GCNT3 IC50: 1489.3 ± 111.6). These data indicate that high levels of GCNT3 expression are associated with an increase of sensibility to 5FU in metastatic CRC cells, demonstrating the active function of GCNT3 for diminishing 5FU chemotherapy resistance.

### GCNT3 diminishes cell proliferation, invasion and alters metabolic properties of CRC cells

The increased cell growth and invasive properties are crucial for cancer progression and metastasis formation. Moreover, cancer cells usually acquire metabolic benefits to promote cell survival and proliferation, and we have recently related these metabolic alterations to increased invasive potential. To elucidate how cancer cells modify their carcinogenic properties upon GCNT3 modulation, we performed cell growth and invasion experiments in our GCNT3 cellular models.

**Figure 2:**
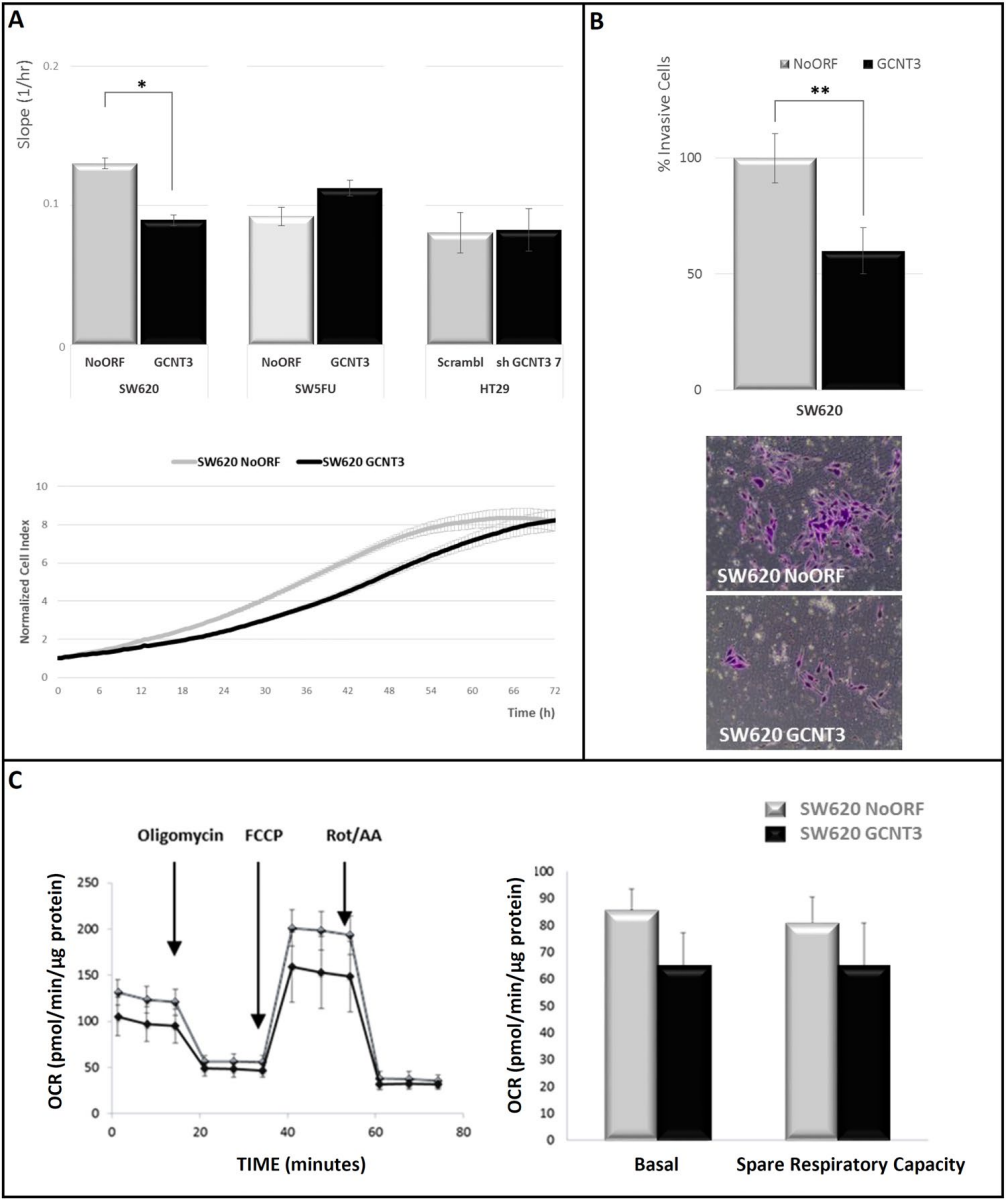
GCNT3 overexpression reduces proliferation, invasion and changes metabolic capacities of CRC cells. (Panel A) xCELLigence proliferation assay of NoORF, GCNT3, Scramble and shGCNT3 7 cells. The rate of proliferation is determined by analyzing the slope of the proliferation line between the 12 and 60 h interval. Complete growth curve of NoORF and GCNT3 SW620 cells from 0 to 72 h after seeding is represented. Results were expressed as 12 h normalized cell index value. Data are represented as mean ± SEM of four independent experiments each performed in triplicate. Student’s t test was applied to assess statistically significant differences (*p < 0.05). (Panel B) Boyden chamber transwell assay of GCNT3 SW620 cell invasion through Matrigel. After 96 h, SW620 cells were fixed and stained with crystal violet (bottom panels) and counted under an optical microscope. Pictures were taken using an Olympus CKX41 microscope (Olympus, Tokyo, Japan), with a 20X LCAch objective and registered using analysis getIT software (Olympus). Scale bars 100 µm. Data are represented as mean ±SEM of three independent experiments each performed in triplicate. Student’s t test was applied to assess statistically significant differences (**p < 0.01). (Panel C) Oxygen consumption rate (OCR) of NoORF and GCNT3 SW620 cells. Bioenergetics parameters were obtained by adding 2 µM Oligomycin to block ATP-linked OCR, 0.2 µM FCCP to uncouple mitochondria for maximal OCR and 0.5 µM Rotenone/Antimycin A (Rot/AA) to shut down mitochondrial respiration. Right panel reflects the quantification of basal respiration (oxygen consumption used to meet cellular ATP demand, calculated by subtracting non-mitochondrial OCR obtained upon Rot/AA addition) and spare respiratory capacity (capability to respond to an energetic demand, calculated as the difference between maximal and basal OCR). Left panel represents OCR measurements over time for cells stably expressing NoORF or GCNT3. We show representative experiments of 6 measures (n = 3).

We used the xCELLigence™ system to real-time *in vivo* assay cell proliferation and viability (Fig. 2. Panel A). We observed that GCNT3 over-expression reduced cell growth in SW620 metastatic cells (31.3% of reduction in growth curve slope (1/hr), p-*value* = 0.02). These changes were not observed in resistant SW5FU cells or in the non-metastatic HT29 model. Next, we tested invasive capacities of GCNT3 cells. We measured cell invasion starting from a serum free environment to rule out proliferation differences (Fig. 2. Panel B). Significantly, 40% less GCNT3 SW620 cells were able to invade through matrigel when compared to control cells (p-*value* = 0.02). We did not observe such differences in 5FU resistant cells (data not shown).

It is well know the relationship between metabolic alterations and different malignant aspects of tumor cells, such as the pro-proliferative Warburg effect^22^. To further investigate GCNT3 effect on reducing the malignant behavior, we checked the glycolytic potential, as the aerobic glycolysis is one of the most remarkable features of proliferative cancer cells. Extracellular acidification rate was measured to assay glycolytic function of GCNT3 and NoORF SW620 cells. However, we were not able to find a differential behavior between both lines (data not shown). Then, we tested the oxidative metabolism reflecting the mitochondrial activity of our models (Fig. 2. Panel C). Curiously, GCNT3 cells presented lower oxygen consumption rate (OCR) than NoORF cells (both OCR, basal and stressed (Spare Respiratory Capacity)), highlighting metabolic differences after GCNT3 re-expression.

All these data support the anti-carcinogenic role of GCNT3 re-expression by reducing cell growth and invasion. Moreover, our data suggest that different metabolic performances are associated with a GCNT3 increase in cancer cells.

### The genomic and proteomic landscapes of GCNT3 are linked to cell cycle and response to drug pathways

**Figure 3:**
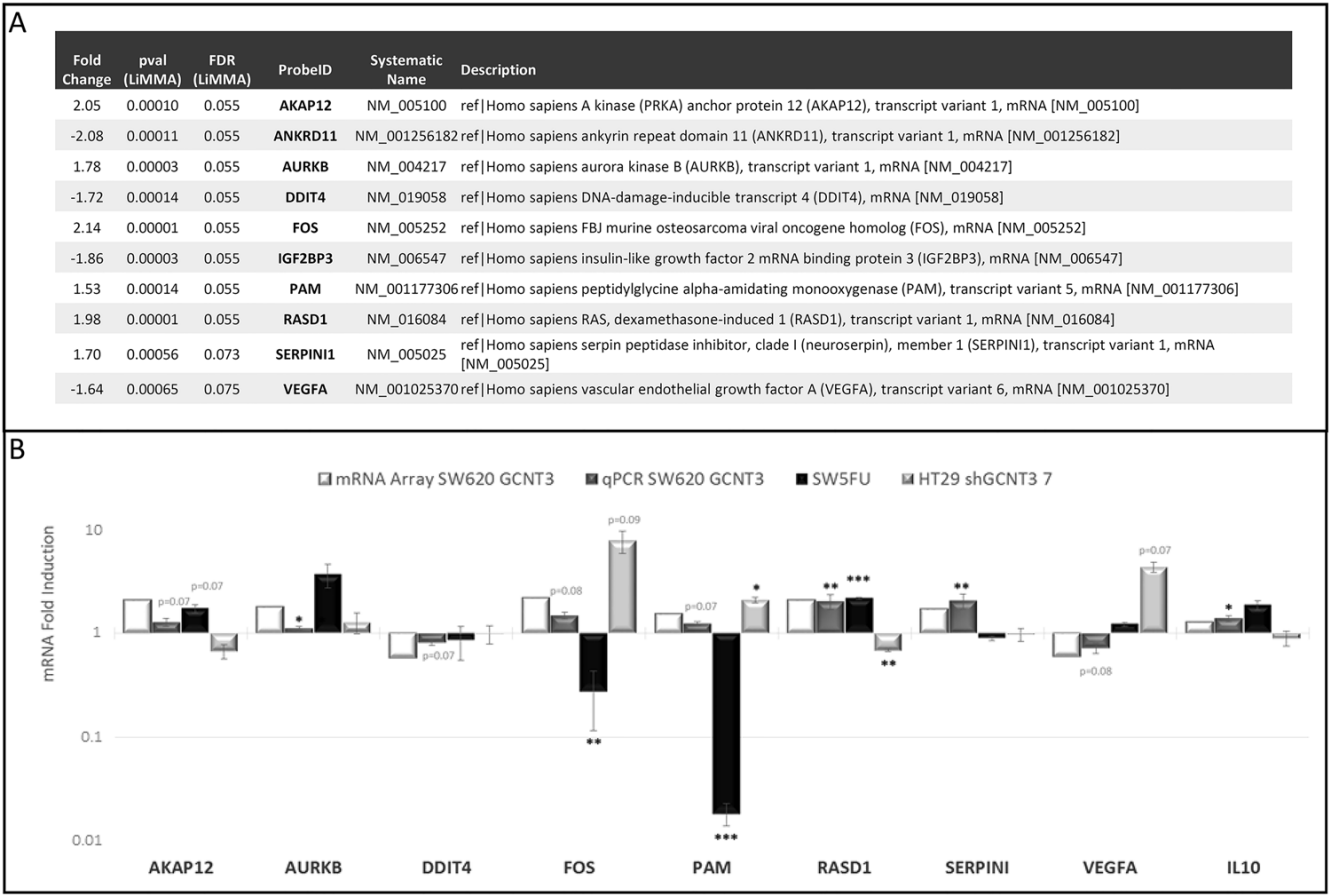
Genomic analysis of GCNT3 overexpression. (Panel A) Description of candidate genes selected for validation. (Panel B) Experimental validation of GCNT3 microarray results by qRT-PCR. Bar graph showing the correlation of microarray data with qRT-PCR transcript levels in three CRC cellular models. The X axis shows the selected panel of validated genes and the Y-axis represent the relative fold change by microarray or qRT-PCR. Data represent mean ± SEM of three independent experiments each performed in triplicate. Student’s t test was applied to assess statistically significant differences (*p < 0.05, **p < 0.01, ***p < 0.001).

With the aim of identifying GCNT3 putative partners, regulators and/or downstream targets, we performed genomic and proteomic analyses in GCNT3 SW620 cells. Data from three independent experiments were assayed in a whole genome microarray analysis. We compared patterns of global expression between GCNT3 and NoORF SW620 cells. As expected, GCNT3 probe showed the highest fold change in gene expression (7.7). To select putative genes regulated by GCNT3 over-expression, we established a cut-off based on a LiMMA p-value < 0.001 (FDR p-value = 0.08). We applied a second cut-off, based on fold change gene expression (±1.5). Our analysis showed 152 differentially deregulated probes (34 upregulated and 118 downregulated, Supplementary Table 3). Among them, we selected ten candidate genes for validation, according also to bibliographic criteria (Fig. 3. Panel A). We used GeneCodis3 tool to performed Gene ontology (GO) analysis and we identified 93 over-represented GO-terms (Supplementary Table 4), that included: mitotic cell cycle, M phase of mitotic cell cycle, cell division, mitotic prometaphase, mitosis, cell proliferation and response to drug.

**Figure 4:**
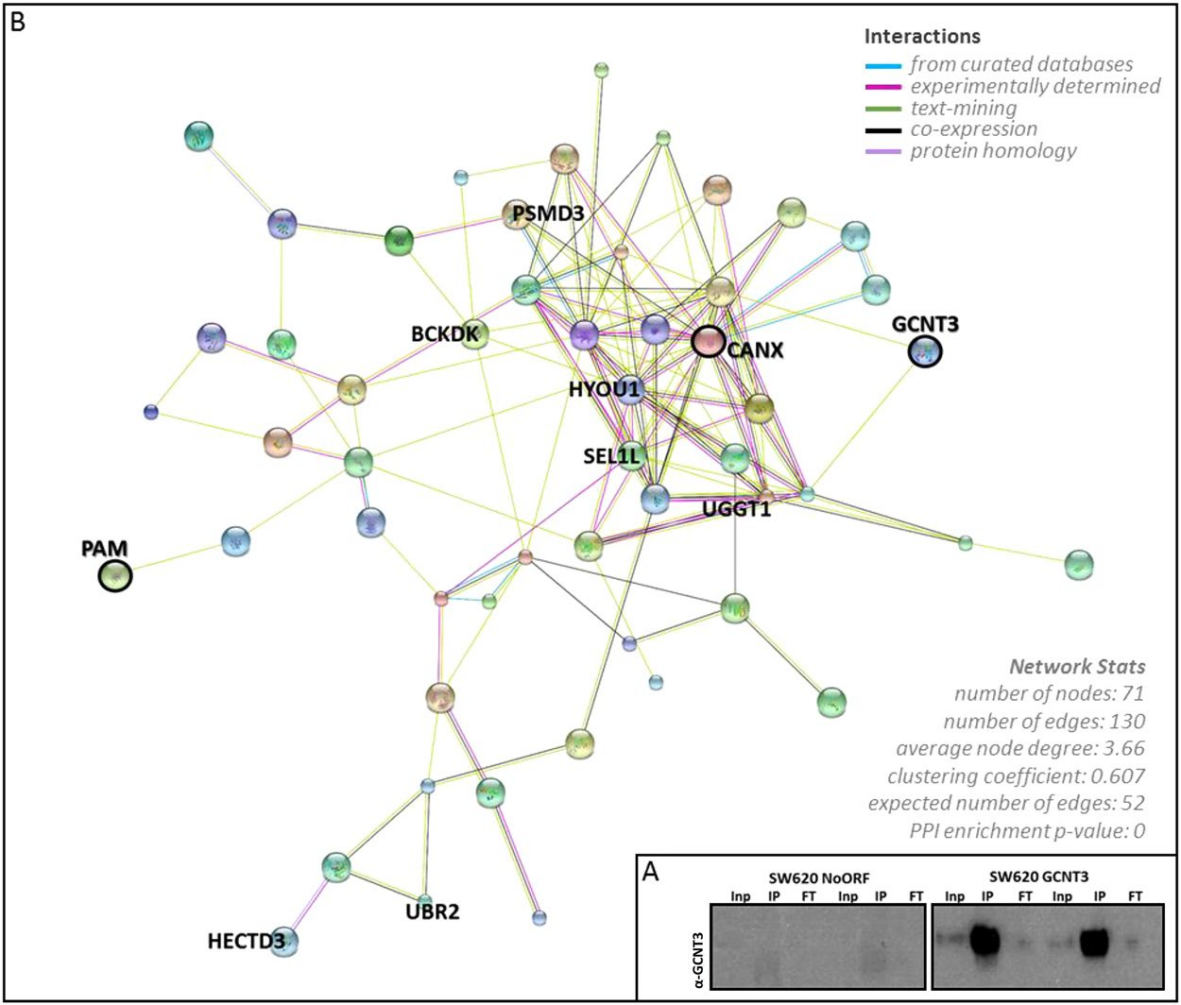
Proteomic analysis of GCNT3 overexpression. (Panel A) Immunoprecipitation of NoORF and GCNT3 SW620 cell extracts using V5 antibody. V5-GCNT3 tag protein was detected by Western blot (Inp: Input, IP: Immunoprecipitate, FT; Flow through). Experiments performed in duplicate. Full-length blots/gels are presented in Supplementary Fig. 3. (Panel B) Protein-protein interaction network of GCNT3 using STRING. Statistically significant interactors of proteomic study were included in the analysis (p-value > 0.0005). Only top-10 statistically significant proteins included in nodes are named, as well as, PAM and CANX.

To identify proteins that directly interacted with GCNT3, we opted for immunoprecipitation using the V5 monoclonal antibody in GCNT3 SW620 cells, followed by proteomic assay. Western blot analysis confirmed that GCNT3 was reliably immunoprecipitated (Fig. 4. Panel A). Overall, we were able to obtain 135 statistically significant measurements of interactors (Supplementary Table 5). The comparison of statistically significant results for both, genomic and proteomic approaches, showed 2 shared genes/proteins: PAM, a peptidyl-glycine alpha-amidating monooxygenase, and CANX or calnexin.

We illustrated protein-protein interaction networks using STRING (Fig. 4. Panel B). We included statistically significant interactors (p-value < 0.0005) and we only represented those implicated in the 71 nodes that we obtained. We highlighted Top-10 statistically significant proteins included in nodes as well as, common genes/proteins for both approaches (PAM and CANX).

The GO analysis of proteomic study, identified 231 GO terms (Supplementary Table 6) that included: cellular protein metabolic process, nucleosome assembly, mitotic cell cycle, cell cycle checkpoint, G1/S transition of mitotic cell cycle and response to drug.

**Figure 5:**
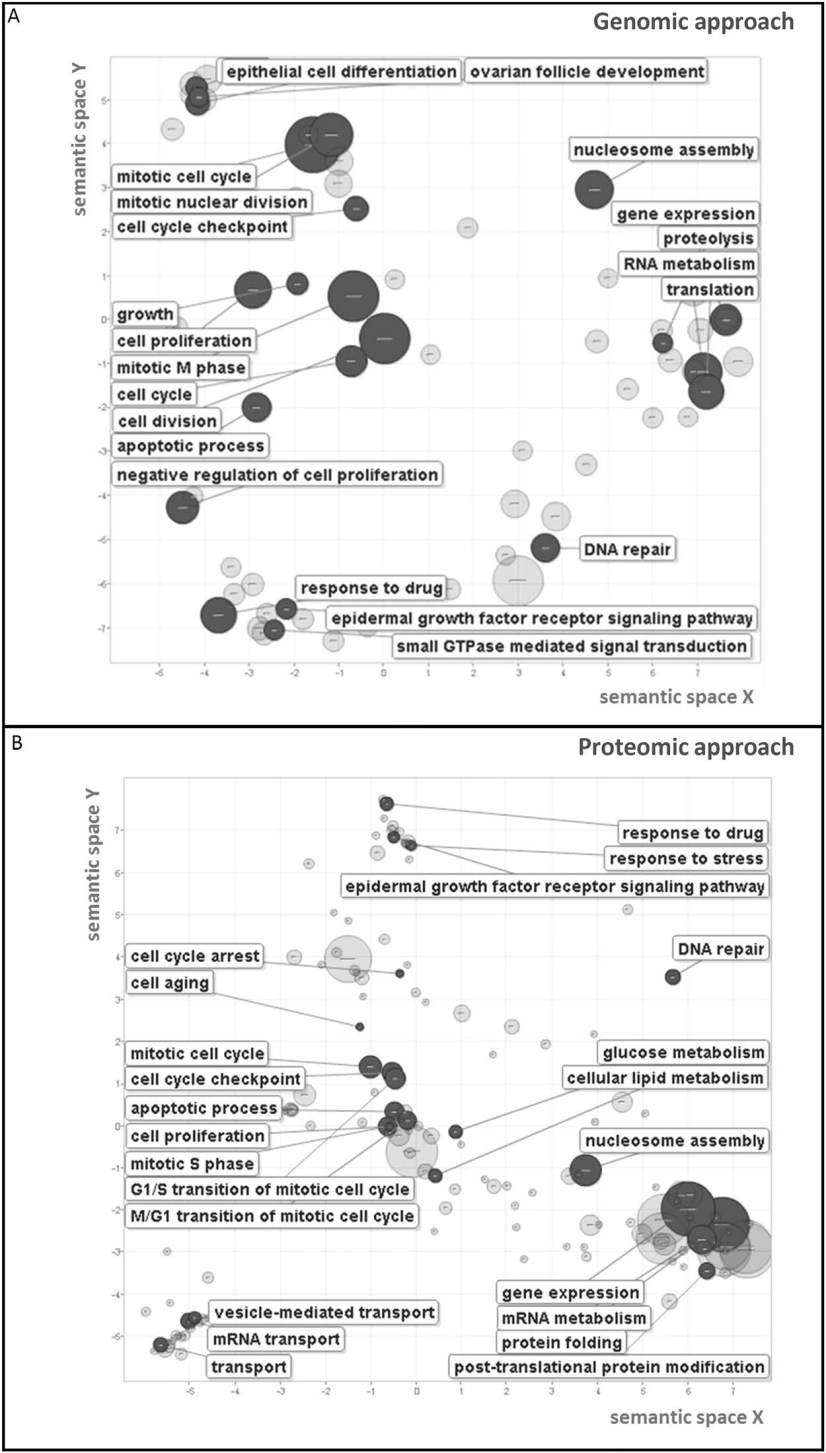
Biological processes enriched by GCNT3 overexpression in CRC cells. REViGO Scatterplot of GO categories enriched in GCNT3 genomic (Panel A) and proteomic (Panel B) analysis. GO enrichment analysis for statistically significant transcripts and proteins. The remaining terms after the redundancy reduction were plotted in a two dimensional space. Bubble sizes indicates the p-value (log10 p-value). Semantic space is based on the semantic similarity, which is the degree of relatedness between two entities by measuring the similarity of their annotation meanings. The list of enriched GO terms is subjected to redundancy reduction, based on the “most informative common ancestor” approach in REVIGO and is represented by cluster representatives in a scatterplot. The x- and y-axes of the scatterplot represent the distance between the cluster representatives.

To clarify GCNT3 GO analysis, we used REViGO tool for removing redundancies in GO terms, of GCNT3 genomic (Fig. 5. Panel A) and proteomic (Fig. 5. Panel B) studies. We observed that the overexpression of GCNT3 shared several semantic spaces in both approaches, being important those linked to cellular cycle, mitosis and proliferation, response to drugs, epidermal growth factor receptor signalling pathway, gene expression and metabolism.

### Independent validation of microarray data in various cellular models of invasiveness and 5FU resistance

We performed qPCR validations of microarray data by three independent experiments in several cellular models: GCNT3 SW620 (metastatic/invasive), GCNT3 SW5FU (resistant) and shGCNT3 HT29 (non-metastatic/non-invasive).

Six out of ten selected genes for validation were upregulated and 4 of them were downregulated in microarray analysis of GCNT3 SW620 cells (Fig. 3. Panel A). Moreover, we also included for validation two interesting genes that were not initially included because they did not satisfy the second cut-off criteria (fold change gene expression (±1.5)). They were: *CANX* (Fold change, 1.32), the calnexin that was also statistically significant in the proteomic analysis and *IL10* (Fold change, 1.28), an interleukin implicated in response to drug (GO:0042493).

We have validated microarray expression data for all selected genes in SW620 cells (Fig. 3. Panel B) but *ANKRD11*, *IGF2BP* and *CANX*, whose expression levels did not show differences between GCNT3 and NoORF cells (data not shown). All validation experiments were performed three times, attesting to the robustness of the results. We confirmed that mRNA levels of *AKAP12*, *AURKB*, *FOS*, *PAM*, *RASD1*, *SERPINI* and *IL10* were upregulated, and those of *DDIT4* and *VEGFA* were downregulated in GCNT3 SW620 cells. Additionally, we proved that VEGFA protein levels were diminished when GCNT3 was upregulated (Supplementary Fig. 1.)

Then, we analysed mRNA expression of validated genes in GCNT3 SW5FU cells. As expected, as cells came from the same cellular origin, several genes had identical behaviour in SW620 and SW5FU lines (*AKAP12*, *AURKB*, *DDIT4*, *RASD1* and *IL10*). However, four out of nine validated genes did not follow the previous tendency which suggests that they could be implicated in the acquisition of resistance to 5FU. These genes were *FOS*, *PAM*, *SERPINI* and *VEGFA*.

Finally, we measured mRNA expression of validated genes in the HT29 non-metastatic/non-invasive GCNT3 inhibition model and we confirmed that *AKAP12*, *IL10* and specially *RASD1* and *VEGFA* have opposite gene expression effects to those found in GCNT3 SW620 model. These data represent an extra-validation of our results.

Overall, these results highlight VEGFA as a putative relevant partner of GCNT3 functions in cell invasion and resistance to drug.

### GCNT3 high-expressing Stage III-IV EOC patients have better response to conventional treatment and clinical outcome

We aim to explore the potential utility of GCNT3 as marker of prognosis and response to treatment in other types of cancer that were frequently diagnosed at advanced stages. Resistance to chemotherapy is among the most relevant problems in the management of ovarian cancer and the O-glycan pathway had been previously associated with *in vitro* drug sensitivity and overall survival in ovarian cancer^23^. Besides, our genomic analysis of GO pathways found an intriguing role of GCNT3 in ovarian follicle development (Fig. 5. Panel A). Finally, VEGFA have been also related to ovarian cancer prognosis^24,25^. All these considerations led us to examine the potential relevance of GCNT3 on epithelial ovarian cancer (EOC).

We performed survival analysis based on *GCNT3* expression in a series of 56 EOC of advanced FIGO stage (III and IV) and high grade (2 and 3) patients. The final dataset included 48 and 8 samples of stages III and IV respectively. Twenty tumours were classified as grade 2 and 35 as grade 3. Median age at diagnosis was 56 years (range 35–85). Median time to treatment failure (TTF) was 16.5 months (range 1–46), and median overall survival (OS) was 40 months (range 2–75). We identified recurrence in 48 patients (85.7%) of which 35 patients (65.4%) died. Sixty-eight percent of the patients achieved a complete response to initial therapy. A brief description of studied population is shown in Supplementary Table 2.

**Figure 6:**
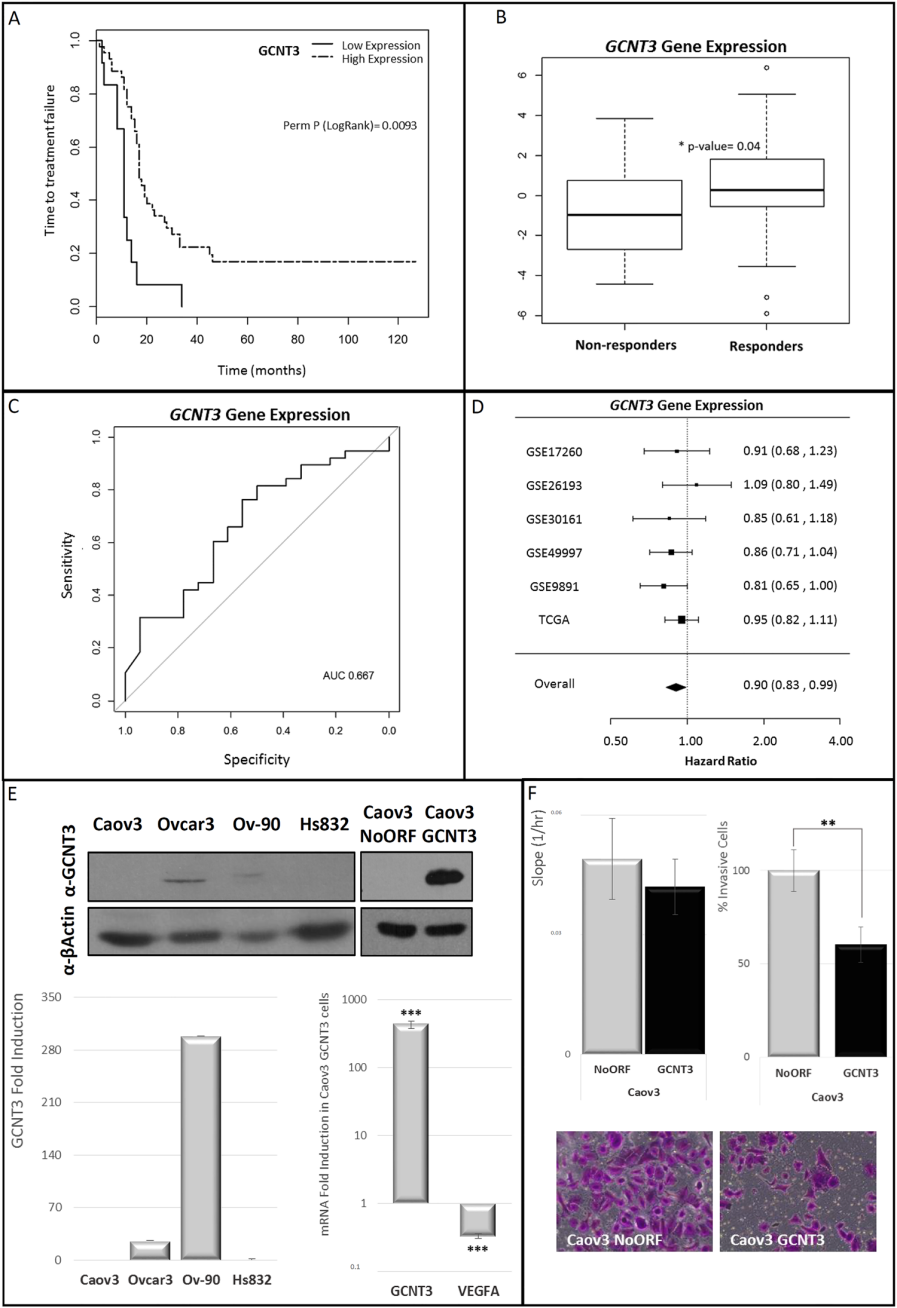
Clinical relevance of GCNT3 expression in epithelial ovarian cancer (EOC). (Panel A) Association between GCNT3 expression and time to treatment failure (TTF) in EOC. Kaplan–Meier plots for GCNT3 expression in 56 EOC patients. (Panel B) Association of GCNT3 gene expression profiles with response. The expression levels of GCNT3 in non-responders and responders groups are shown. The median value of GCNT3 expression is indicated by the horizontal bar on the graph (Man-Whitney U-test for P values). (Panel C) ROC analysis of the GCNT3 signature in EOC patients. The AUC was 0.667. (Panel D) Forest plot showing the meta-analysis of hazard ratio (HR) and 95% confidence interval (CI) estimates for TTF for the prognostic significance of GCNT3 expression in EOC patients from six different studies. (Panel E) Protein and mRNA expression levels of GCNT3 in non-infected EOC cells and Caov3 stable cell lines overexpressing GCNT3. Proteins were detected by western blot using specific antibodies against GCNT3 and β-Actin. mRNA expression levels of GCNT3 and VEGFA were measured by RT-QPCR. Data represent mean ± SEM of three independent experiments. Student’s t test was applied to assess statistically significant differences (***p < 0.001). Full-length blots/gels are presented in Supplementary Fig. 4. (Panel F) Boyden chamber transwell assay of GCNT3 Caov3 invasion through Matrigel. After 96 h, Caov3 cells were fixed and stained with crystal violet (bottom panels) and counted under an optical microscope. Pictures were taken using an Olympus CKX41 microscope (Olympus, Tokyo, Japan), with a 20X LCAch objective and registered using analysis getIT software (Olympus). Scale bars 100 µm. On the left, xCELLigence proliferation assay of NoORF and GCNT3 Caov3 cells. The rate of proliferation is determined by analyzing the slope of the proliferation line between the 12 and 60 h interval. Data are represented as mean ± SEM of three independent experiments each performed in triplicate. Student’s t test was applied to assess statistically significant differences (**p < 0.01).

*GCNT3* expression was distributed differentially within these patients and Kapplan-Meier plots for TTF of *GCNT3* showed an association between high expression of this gene and better clinical outcome than those with lower *GCNT3* expression (Fig. 6. Panel A). Accordingly, risk of relapse by hazard ratio for *GCNT3* high-expressing ovarian cancer patients was 0.31 (CI 95%: 0.15–0.61, p = 0.02). We didn’t find associations of *GCNT3* expression within clinical variables (age and grade), suggesting that the effect of *GCNT3* gene expression and TTF was independent of these clinical factors.

Interestingly, *GCNT3* expression was associated with response to initial therapy (standardized protocol with a combination of taxane and platinum agents after debulking surgery). Those patients who are responders to initial therapy showed higher levels of *GCNT3* expression (p = 0.04) than non-responders (Fig. 6. Panel B). Logistic regression analysis showed statistically significant association between *GCNT3* expression and response to treatment (OR: 1.26 (CI 95%: 0.99–1.6, p = 0.04)). Besides, the area under the ROC curve (AUC) for predicting response, was found to be 0.67 (CI 95%: 0.51–0.82) (Fig. 6. Panel C).

These results suggest that *GCNT3* could constitute a prognostic and predictive factor in EOC since its overexpression is significantly associated with better clinical outcome and response to initial therapy.

To validate our results, we performed a meta-analysis based on *GCNT3* expression in a large series of EOC using publicly available gene expression datasets. The analysis comprised a total number of 1126 EOC patients. We investigated whether *GCNT3* expression was associated with relapse in six studies included in curatedOvarianData database^26^ for which TTF information was available. *GCNT3* expression had an overall protective effect for grade 2 and 3 patients, and the pooled HR, adjusted for grade and FIGO stage, was significantly less than 1 (HR = 0.90, (CI 95%: 0.83–0.99, p = 0.02)) (Fig. 6. Panel D). Also, *GCNT3* expression in FIGO stage III and IV patients had an adjusted overall HR of 0.92, (CI 95%: 0.84–1.01, p = 0.07). These data consistently validate our findings that upregulation of *GCNT3* is associated with better outcome in high grade EOC patients.

### GCNT3 also diminishes cell invasion and *VEGFA* expression in EOC cells

In order to further explore the role of GCNT3 in EOC prognosis, we tested GCNT3 expression in a panel of four ovarian cell lines. We found that GCNT3 levels of expression (protein and mRNA) were almost abolished in Caov3 cell line and in benign Hs832 ovarian cells. We observed measurable levels of GCNT3 protein and mRNA in Ovcar3 and Ov-90 cells (Fig. 6. Panel E). We decided to generate a GCNT3 overexpression model in Caov3 cells (Fig. 6. Panel E). To interrogate whether Caov3 cells modify their carcinogenic properties upon GCNT3 re-expression, we performed cell growth and invasion experiments following the same approach performed with CRC cellular models. We found that GCNT3 over-expression did not significantly change cell growth in Caov3 cells, however we can appreciate a trend in reduction cell growth (Fig. 6 Panel F). Next, we measured cell invasion and we observed that 40% less GCNT3 Caov3 cells were able to invade through matrigel when compared to control cells (p-*value* = 0.001) (Fig. 6 Panel F). These data confirm previous results in CRC cellular models and they validate the role of GCNT3 in cell invasion. Besides, we also validated by qPCR and western blot that, *VEGFA* expression was downregulated in GCNT3 Caov3 cells (Fig. 6. Panel E and Supplementary Fig. 1). Thus, GCNT3 role in cell invasion and drug resistance could be mediated through VEGFA in these two different cancer models, CRC and EOC, with relevant clinical implications.

## Discussion

A crucial point on cancer management is to diagnose patients who would benefit from particular therapies. Cancer patients’ were usually stratified by tissue-specific scores such as the Dukes’ in CRC^27^, or FIGO in EOC^28^ and, in general, these have been generalized by TNM staging^29^. Several molecular characterizations have been proposed as prognostic biomarkers for many cancer types. However, most of these biomarkers are only useful for a specific cancer type or even specific subtypes^30^. More interesting is to identify biomarkers that can be used for multiple types of cancer. The researcher’s challenge is to advance in our ability to predict response in the clinic and to identify patients who are most likely to benefit from certain drugs.

*GCNT3* pattern of expression have been mainly associated with colon cancer prognosis^11,13,15–17^, but also with pancreatic cancer^12^ and hepatocellular carcinoma^10^. GCNT3-deficient mice have impaired the mucosal barrier and increased susceptibility to colitis^31^. Moreover, they exhibited significantly more colon damage with increased ulceration and increased damage to the crypts in the colon. Finally, it have been also reported that absence of GCNT3 results in a defect in the immune system, characterized by a reduction in immunoglobulin levels that may be associated with an increase in disease susceptibility^31^.

Understanding the basis of GCNT3 glycosyltransferase action in different types of cancer will provide us new clues about the carcinogenesis and associated drug resistance processes. Moreover, a cancer-type glycosylation signature have been recently described highlighting the role of this type of enzymes on cancer profiling and prognosis^32^ and the O-glycan pathway have been associated with *in vitro* sensitivity to gemcitabine and overall survival in ovarian cancer^23^. In this context, we explored CRC and EOC cellular models of GCNT3 over-expression and inhibition (Fig. 1. Panels A, B and C and Fig. 6. Panel E).

5FU is the most extensively used CRC treatment, frequently combined with other chemotherapeutic drugs^33^. We characterized 5FU growth inhibition in our panel of CRC cells where we observed a correlation between GCNT3 endogenous expression and 5FU sensitivity. Moreover, we validated GCNT3 mRNA induction after 5FU treatment^34^. Next, we compared 5FU growth inhibition in GCNT3 SW620 cells and, remarkably, we detected a clear reduction in IC50 parameter indicating that GCNT3 is increasing cell sensitivity to the drug. Besides, we also observed a trend in 5FU growth inhibition in GCNT3 SW5FU resistant cells, suggesting that GCNT3 is not the unique modulator of 5FU resistance. It had been described that multiple factors might contribute to 5FU resistance and gene expression data suggest that altered regulation of nucleotide metabolism, amino acid metabolism, cytoskeleton organization, transport, and oxygen metabolism may underlie the differential resistance to 5FU (38).

Our analysis of GCNT3 overexpression in in SW620 cells demonstrates that GCNT3 reduces cellular growth and invasion which means an inconvenience for tumor development and expansion (Fig. 2. Panels A and B). These results, in part, have been previously described^11^, but here, we have validated them in other cellular models and using different technologies. This is the first time that metabolic capacities of GCNT3 cells have been analyzed, and results showed that they display lower mitochondrial respiration than control cells. Then, GCNT3 acts at the level of metabolic reprogramming, one of the emerging hallmarks of cancer^35^. Our results demonstrate a cancer suppressive activity of GCNT3, as its re-expression is accompanied by diminished protumoral characteristics such as growth, invasion and OCR.

Besides GCNT3 anti-carcinogenic properties, as many glycosylases, it must be taking part of a complex network of interactors and modulators^10,32^. In an attempt to gain insight into the molecular mechanism underlying the effect of GCNT3 overexpression, we studied for first time, using genomic technologies, the GCNT3 landscape of targets, interactors and regulators. Our goal was to find new potential interactors and modulators of GCNT3 *in vivo*. Microarray analysis of GCNT3 cells has revealed 152 putative deregulated probes. Several genes were validated by qPCR: *AKAP12*, *AURKB*, *DDIT4*, *FOS*, *IL10*, *PAM*, *RASD1*, *SERPINI1* and *VEGFA* (Fig. 3). Moreover, *PAM* and *CANX* or calnexin, were putative regulators and direct interactors of GCNT3 as they were statistically significant in both genomic and proteomic approaches. However, we were not able to validate CANX by qPCR. Proteomic study also revealed 133 more putative previously unknown GCNT3 interactors and in future studies, we will take advantage of these additional results (Supplementary Table 5).

Both lists of genes and proteins allowed us to obtain relevant information about main pathways influenced by GCNT3 action. After removing redundancies in GO terms, we observed that GCNT3 overexpression is mainly linked to pathways related to cellular cycle, mitosis, proliferation, epidermal growth factor receptor signaling and, importantly, response to drugs (Fig. 5.). Since 1985, it have been described that substantial alterations of the cellular glycoprotein pattern are expected to occur during cell proliferation^36^ which is in concordance with our results. Besides and interestingly, other glycosyltransferase such OGT (O-GlcNAc transferase) is necessary and essential for G0/G1 transition^37^, cell-cycle regulation^38^ and during mitosis, ensuring correct chromosomal segregation^39^. O-glycan pathway had been previously associated with drug sensitivity (31) and here, we confirmed our hypothesis of GCNT3 implication in the drug response processes. Our study is the first in which GCNT3 is directly linked to these pathways. Together, these results expand upon the classical GCNT3-associated functions and provide us new insights about GCNT3 transcriptional networks in cancer cells.

Our study points out VEGFA as an important player in GCNT3-associated functions in cancer and drug resistance. We hypothesized that IL10 could also collaborate in such functions. It has been suggested that IL10, an immunosuppressive cytokine, stimulates *VEGFA* gene expression^40^. VEGFA is a 34- to 42-kDa, dimeric, disulfide-bound glycoprotein^41^ that could be modified by O-glycosilation. It have been demonstrated that higher *VEGFA* expression is associated with increased vascular density, development of metastasis and drug resistance, indicating poorer prognosis in CRC^42^.

We moved the GCNT3 expression analysis to EOC where, clinically, better or alternative methods to identify cancer risk groups are also needed. We assayed the possible role of *GCNT3* expression as a prognostic biomarker in 56 stage III-IV EOC patients. Due to the difficulty in obtaining a new set of samples, we have validated our results by meta-analysis in more than one thousand patients from six different datasets. We found that patients who presented lower expression of *GCNT3* were significantly more likely to present treatment failure than those with a higher expression (Fig. 6. Panels A and D). Moreover, we found an association between *GCNT3* expression and response to treatment and the area under the ROC curve (AUC) for predicting response, was found to be 0.67 (Fig. 6. Panels B and C). This suggests that *GCNT3* expression analysis might be a promising tool to predict response to conventional treatment and thus contributing to personalized medicine. Moreover, there is also a possibility that these treatments are effective only in the patients with higher GCNT3 indicating that these drugs work through GCNT3 mediated mechanisms.

The aforementioned results, as well as the *in vitro* studies in EOC cells reporting the role of GCNT3 in inhibiting tumour invasion (Fig. 6. Panel F), point to GCNT3 as a promising target in EOC therapy. Moreover, like in CRC, in EOC cells GCNT3 overexpression is associated with diminished *VEGFA* expression (Fig. 6. Panel E). VEGFA have been also implicated in ovarian cancer prognosis^24,25^. Interestingly, it is well known that crucial growth factors like VEGFA, that sustain cancer cells or their receptors (VEGFR), could be targeted using antibodies or protein tyrosine kinase inhibitors^43^. Although our findings were highly consistent, further *in vivo* experiments and analysis of GCNT3 in different stages of EOC are needed to validate the role of GCNT3 in ovarian cancer.

Overall, our results open new perspectives of GCNT3 in the translational biomarker research. GCNT3 could be used for the stratification CRC and EOC patients with high risk of relapse, and also might be a biomarker to monitor the response to treatment. Agents that induce the expression of *GCNT3* might be potential antitumor drugs for CRC and EOC, with the aim of reducing adverse events and overcoming drug resistance that is a current and necessary demand for patients and health systems.

## Materials and Methods

### Cell culture, Treatments and Stable cell lines generation

CRC, EOC, and HEK-293T cells were obtained directly from American Type Culture Collection (ATCC, Middlesex, UK). ATCC performs cell line authentication through STR profiling and mycoplasma contamination testing. Frozen aliquots were stored and cells were passaged in the laboratory for fewer than 6 months after resuscitation. Cells were cultured and maintained under manufacture’s conditions. Cells were treated with vehicle or 5-fluorouracil (5FU) (Sigma-Aldrich, St. Louis, MO, USA) at effective antitumor concentrations. We generated and characterized SW5FU cells by exposing SW620 cells to increasing concentrations of 5FU for 15 months as previously described^34^.

Generation of GCNT3 stable overexpression models and shGCNT3 cells was performed using lentiviral systems as previously described^44^. HEK-293T cells were transfected using Lipofectamine 2000 (Life Technologies, Thermo Fisher Scientific, Waltham, MA, USA) with lentiviral vectors expressing V5-GCNT3/NoORF empty vector (DNA 2.0, Newark, California, USA), or with Mission specific lentiviral vectors (Sigma-Aldrich, St. Louis, MO, USA) along with a set of packaging plasmids (Addgene, Cambridge, MA, USA).

### Antibodies, Western blotting and Immunofluorescence

We used primary antibodies against GCNT3 (Sigma-Aldrich, St. Louis, MO, USA, HPA011154), V5 Epitope Tag (Invitrogen- Thermo Fisher Scientific, Waltham, MA, USA, R960-25), Vascular endothelial growth factor A (VEGFA) (abcam, Cambridge, UK), α-Tubulin (Sigma-Aldrich, St. Louis, MO, USA, T9026) and β-Actin (Sigma-Aldrich, St. Louis, MO, USA, A1978). The following secondary antibodies were used: Horseradish peroxidase conjugated antibodies anti-mouse and anti-rabbit (Merk-Millipore, Darmstadt, Germany), Alexa 594-conjugated anti-mouse and Alexa 488-conjugated anti-rabbit antibodies (Invitrogen-Thermo Fisher Scientific, Waltham, MA, USA). Cells were lysed and proteins were separated by SDS–polyacrylamide gel electrophoresis and transferred into a nitrocellulose membrane (Bio-Rad, Hercules, California, USA). Immunofluorescence assays were performed as previously described^44^.

### Gene expression analysis

We used RNeasy Mini Kit or RNeasy FFPE Kit (Qiagen, Germantown, MD, USA) to obtain total RNA from cultured cancer cells or from formalin-fixed, paraffin-embedded (FFPE) tumor samples previously deparafinated, respectively.

VeriQuest SYBR Green qPCR Master Mix (Affymetrix, Santa Clara, CA, USA) was used for gene expression analysis of culture cells as previously described^13,17^ (see oligos used in Supplementary Table 1). In clinical samples, *GCNT3* gene expression was analyzed using the specific TaqMan probe (Hs00953355_m1, Life Technologies, Carlsbad CA, USA).

The whole gene expression modulated by GCNT3, was also tested using expression arrays (Human Gene Expression 4 × 44 K v2 Microarray Kit (G4852A-026652)). Comparisons between GCNT3 and NoORF SW620 cells were performed using triplicates. Ten µg of total RNA for each condition were sent to the Genomics Unit of the National Centre for Biotechnology (CNB, Madrid) for RNA quality evaluation, amplification, labelling and hybridization to arrays according to the manufacturer’s protocols. To normalize the data set, loess within-slide normalization and quantiles between-slides normalization were performed. Differential expression was assessed by using the LIMMA method. Benjamini and Hochberg false discovery rate (FDR) was applied for multiple test correction. Genes that showed p-values < 0.001 were considered differentially expressed and an additional cutoff threshold of 50% change in gene expression was used to define a gene as being differentially regulated. Differential expression analysis was visualized using FIESTA viewer^45^.

Functional analysis of Gene Ontology (GO) terms and gene set enrichment analysis were carried out using the GeneCodis3 tool^46–48^. REViGO tool was used to summarize Gene Ontology terms by removing redundant GO terms^49^. All microarray data can be downloaded from the Gene Expression Omnibus (GEO; http://www.ncbi.nlm.nih.gov/geo/) database under accession number GSE88792.

### Proliferation, Cell viability and Invasion assays

Cell proliferation and viability was measured in real-time using the xCELLigence™ system (ACEA Biosciences, San Diego, CA) according to manufacturer’s protocols. After 24 hours, cells were treated with the corresponding 5FU concentration. Real-time monitoring of proliferation and cell viability was performed for 8 days in 30 min intervals. Cell viability was also determined by the 3-(4,5-dimethyl-thyazol-2-yl)-2,5-diphenyl-tetrazolium (MTT) assay as previously described^44^.

Depending on the cellular type, a density between 25000 and 200000 cells per well was seeded in serum-free medium into inserts of a BD MatrigelTM invasion chamber (BD Biosciences, San Jose, CA, USA). Invasive cells were measured as previously described^44^.

### Oxygen consumption rate (OCR) and Extracellular acidification rate (ECAR)

We monitored OCR and ECAR as indicators of mitochondrial respiration and glycolytic function with an XF96 Extracellular Flux Analyzer using XF Cell Mito Stress Test kit and XF Glycolysis Stress kit according to manufacturer instructions (Seahorse Biosciences, North Billerica, MA, USA). OCR and ECAR were measured 3 times following injection of each drug, and normalized to protein content. At least 6 replicates per condition were done for each experiment.

### Immunoprecipitation and Proteomic Assay

Sixty millions of NoORF and GCNT3 SW620 cells (2 replicates) were lysed and 5 mg of total protein were incubated for 1 h at 4 °C with 200 µl protein A beads (Roche, Basel Switzerland) as a pre-clear step. Pre-cleared lysates were incubated overnight at 4 °C with 2 µg of anti V5 antibody followed by 2 h incubation with 100 µl of protein A beads. After incubation, beads were washed and transferred to Proteomic Core Unit of the Spanish National Cancer Research Centre (CNIO) for analysis. Peptides were analyzed by LC-MS/MS, Impact (Bruker Daltonics) coupled online to a nanoLC Ultra system (Eksigent), equipped with nanoelectrospray ion source supplemented with a CaptiveSpray nanoBooster operated at 0.2 bar/minute with isopropanol as dopant. For protein identification and quantification raw data were analyzed by MaxQuant (1.5.1.2) using a Human Uniprot Canonical database plus the most common contaminants (20584 entries in total).

We used STRING (http://string-db.org/) to illustrate protein-protein interaction networks. Functional analysis of Gene Ontology (GO) terms and protein set enrichment analysis were carried out using the GeneCodis3 tool^46–48^. REViGO tool was used to summarize Gene Ontology terms by removing redundant GO terms^49^.

### Patients Selection

A total number of 56 FFPE samples were obtained from ovarian cancer patients of La Paz University Hospital (Madrid, Spain) with the authorization of Ethics Committee of La Paz University Hospital. According to regulations, all patients provided informed consent and experimental protocols were approved by the hospital ethic committee. All methods were carried out in accordance with relevant guidelines and regulations. Clinical and histopathological parameters were prospectively collected (Supplementary Table 2). All cases were of high stage according to the International Federation of Gynecology and Obstetrics (FIGO) (stages III–IV). Patients were treated following a standardized protocol with a combination of taxane and platinum agents after debulking surgery. Samples were collected and basal gene expression was analyzed before treatment. After that, these initial measures of gene expression were correlated with response to treatment. Overall Survival (OS) was defined from the date of primary surgery to the date of patient death. Time to treatment failure (TTF) was defined as the time interval between the start of the treatment and the first confirmed sign of disease recurrence or progression. Response to initial therapy was evaluated according to radiologic imaging criteria.

### Statistical analysis

Student’s t test was used to determine statistically significant differences between groups. All reported p values were two-sided. Statistical significance was defined as p < 0.05. The statistical analyses were performed using the R statistical software version 3.1.1 (www.r-project.org).

*GCNT3* expression (Q) in tumor samples was quantified with the 2–ΔCt method. The Kaplan-Meier method was used to estimate relapse-free survival, and Log-rank test and Univariate Cox regression analysis were used to test the association between relapse-free survival and *GCNT3* gene expression. We applied multivariate proportional hazards Cox regression modeling to adjust for potential confounding factors. Hazard ratios (HR) and 95% Confidence Intervals (95% CI) were calculated from the Cox regression model. An arbitrary cutoff point stratifying high and low *GCNT3* expression was established according to the largest prediction ability evaluated by the c-index methodology^34^ using 100 times 5-fold cross validation. The non-parametric Mann-Whitney test was used to determine the differences in the distribution of *GCNT3* gene expression data among the different series within each clinical variable. Statistical significance was defined as p *value* < 0.05. The curatedOvarianData package^26^ was used for efficient meta-analysis of *GCNT3* expression in ovarian cancer datasets.

### Data availability

All microarray data can be downloaded from the Gene Expression Omnibus (GEO; http://www.ncbi.nlm.nih.gov/geo/) database under accession number GSE88792.

## Electronic supplementary material

Supplementary Information
