# Supplementary material for: The role of glycosyltransferase enzyme GCNT3 in colon and ovarian cancer prognosis and chemoresistance
Source: Sci Rep. 2018 May 31;8:8485. doi: 10.1038/s41598-018-26468-4 (PMC5981315; doi:10.1038/s41598-018-26468-4)
Supplement: Supplementary file 1 — Supplementary Information [file 41598_2018_26468_MOESM1_ESM.pdf]

SUPPLEMENTARY INFORMATION

The role of glycosyltransferase enzyme GCNT3 in colon and ovarian cancer prognosis and chemoresistance

Lara P. Fernández[1], Ruth Sánchez-Martínez[1], Teodoro Vargas[1], Jesús Herranz[2], Roberto Martín-Hernández[2], Marta Mendiola[3,4], David Hardisson[4,5], Guillermo Reglero[1], Jaime Feliu[6,7,8,9], Andrés Redondo[6,7,9] and Ana Ramírez de Molina[1]

Authors's Affiliations: [1]Molecular Oncology Group, IMDEA Food Institute, CEI UAM + CSIC, Madrid, Spain; [2]Biostatistics and Bioinformatics Unit, IMDEA-Food Institute, CEI UAM+CSIC, Madrid; [3]Molecular Pathology Section, Institute of Medical and Molecular Genetics (INGEMM) La Paz University Hospital, Madrid, Spain; [4]Molecular Pathology and Therapeutic Targets Lab, IdiPAZ, La Paz University Hospital, Madrid (Spain); [5]Pathology Department, IdiPAZ, La Paz University Hospital, Madrid (Spain); [6]Translational Oncology Lab, IdiPAZ, La Paz University Hospital, Madrid (Spain); [7]Clinical Oncology Department, La Paz University Hospital, Madrid (Spain); [8]CIBERONC CB16/12/00398, La Paz University Hospital, Madrid (Spain); [9]Catedra UAM-AMGEN, La Paz University Hospital, Madrid (Spain).

Contact Information (Corresponding Author): Ana Ramírez de Molina, Molecular Oncology Group, IMDEA Food Institute, CEI UAM + CSIC. Carretera de Cantoblanco 8, Madrid, Spain. Phone +34672134921. E-mail: ana.ramirez@imdea.org

Running Title: GCNT3 in Colon and Ovarian cancer: Prognosis and Resistance

LIST OF SUPPLEMENTARY MATERIAL

Supplementary Figure 1. Regulation of VEGFA protein expression after GCNT3 overexpression.

Supplementary Figure 2. Full-length blots/gels of Figure 1 (Panel A).

Supplementary Figure 3. Full-length blots/gels of Figure 4 (Panel A).

Supplementary Figure 4. Full-length blots/gels of Figure 6 (Panel E).

Supplementary Figure 5. Full-length blots/gels of Supplementary Figure1 (Panel A).

Supplementary Table 1. Oligos used for mRNA expression assays.

Supplementary Table 2. Clinical characteristics of epithelial ovarian cancer patients

Supplementary Table 3. Genomic analysis. List of 152 differentially deregulated probes.

Supplementary Table 4. Gene ontology (GO) terms obtained from transcriptomic study of GCNT3.

Supplementary Table 5. Proteomic analysis. List of 135 statistically significant GCNT3 interactors.

Supplementary Table 6. Gene ontology (GO) terms obtained from proteomic study of GCNT3 interactors.

Supplementary Figure 1. Regulation of VEGFA protein expression after GCNT3 overexpression.

Panel A) Protein expression levels of VEGFA in stable cell lines overexpressing GCNT3 and shGCNT3. Proteins were detected by western blot using specific antibodies against VEGFA and β-Actin as a loading control. Representative western blot assays of three independent experiments are shown. Full-length blots/gels are presented in Supplementary Fig 5.

Panel B) Protein levels were quantified relative to controls (NoORF or Scramble) by densitometry using Image J.

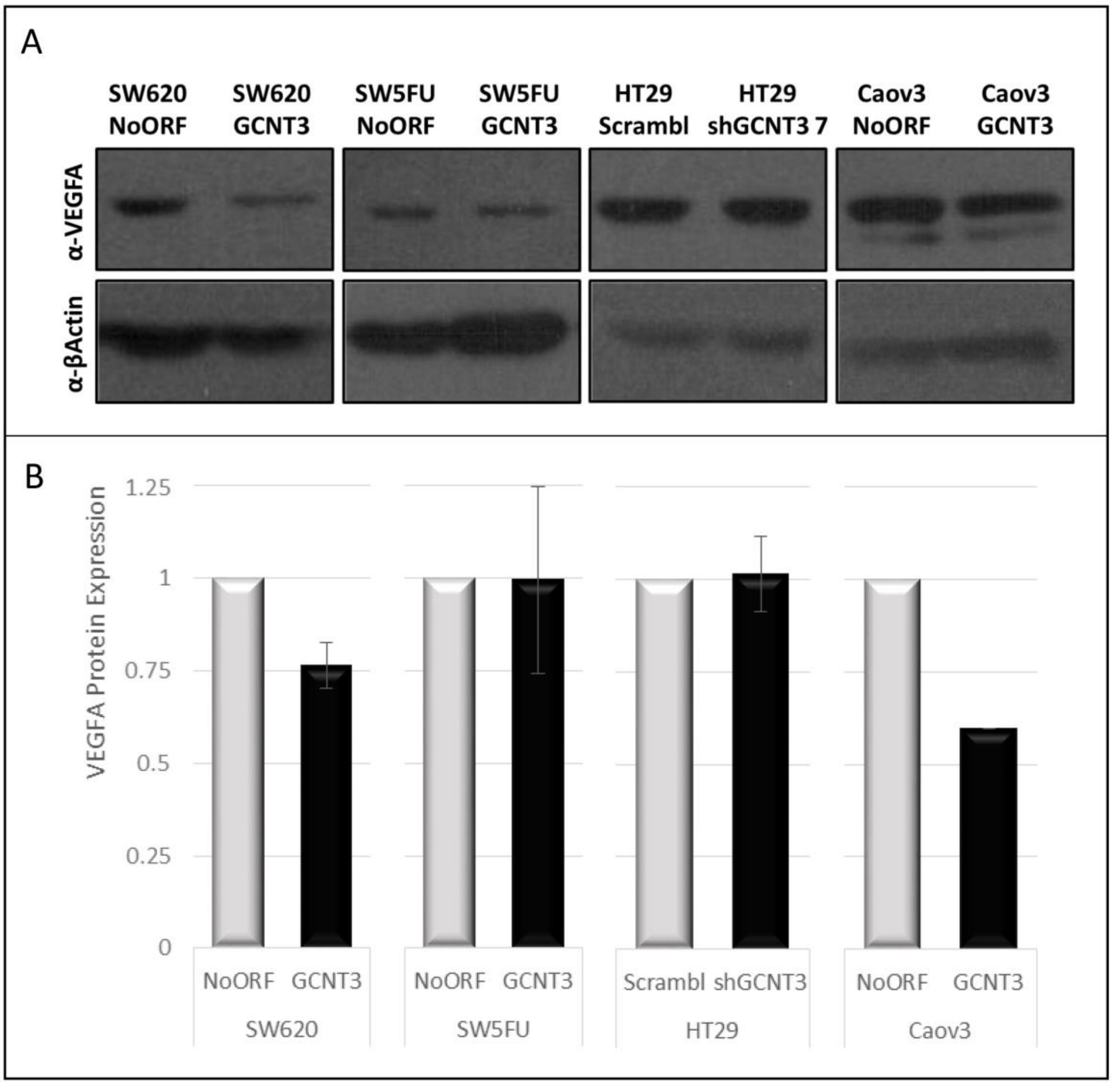

Supplementary Figure 2. Full-length blots/gels of Figure 1 (Panel A).

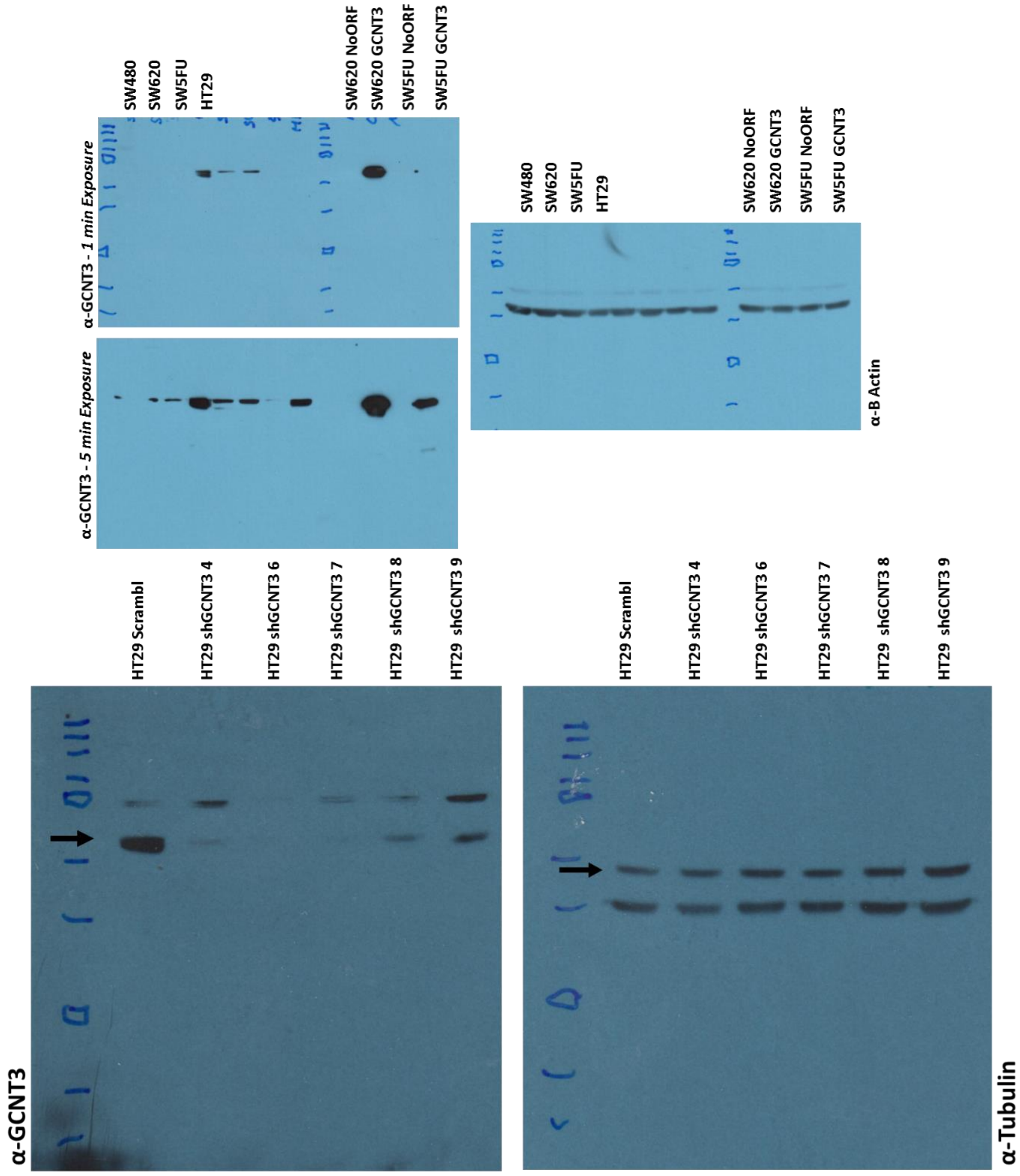

Supplementary Figure 3. Full-length blots/gels of Figure 4 (Panel A).

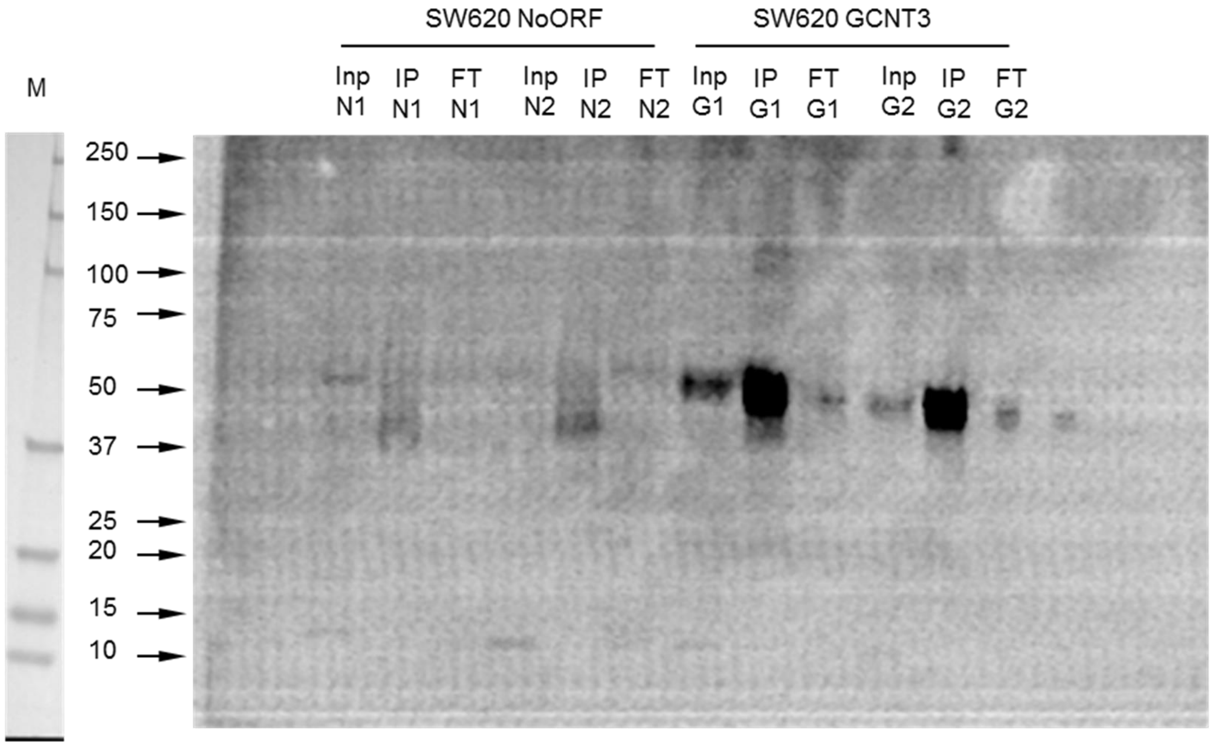

Anti GCNT3 Sigma (Rabbit)

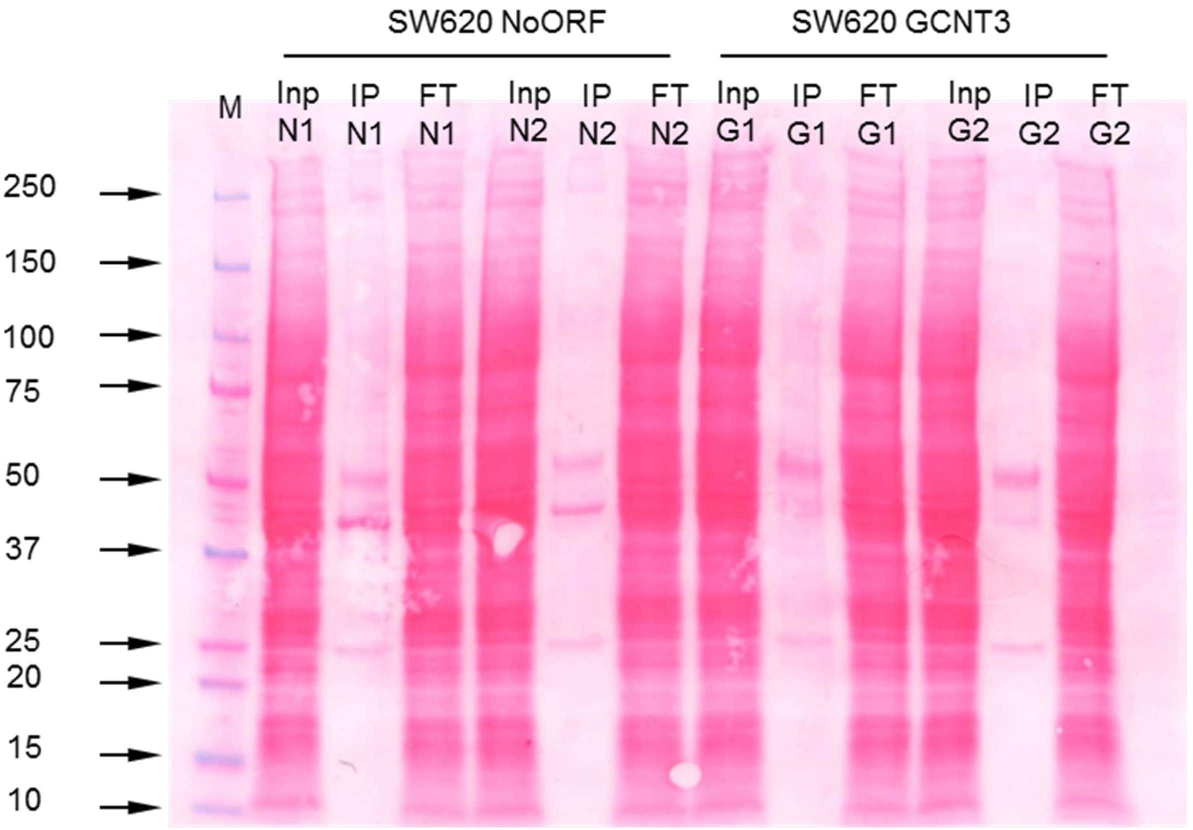

Supplementary Figure 5. Full-length blots/gels of Supplementary Figure1 (Panel A).

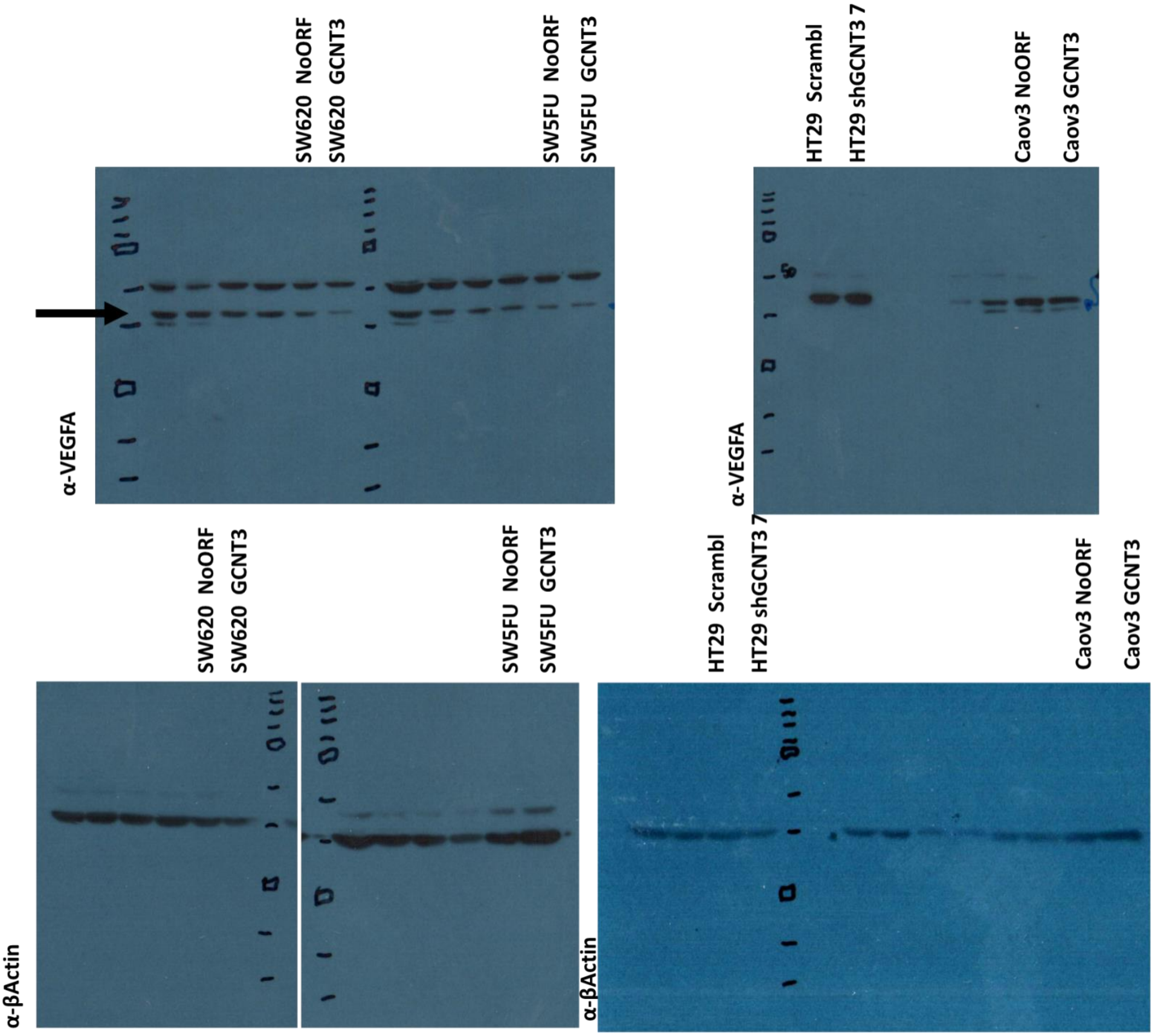

Supplementary Table 1. Oligos used for mRNA expression assays.

Gene	Orientation	Sequence (5'->3')
AKAP12	Forward	CTCCTGCCTTGGGAGTTTGC
	Reverse	GGCCATTTAGGTCACCCTCC
ANKRD11	Forward	ACACGGGATTTGTCGGTGAA
	Reverse	GGGTGCTTTGGTGTTGTGTC
ATR	Forward	GAGTTTTGGCCTCCACACGG
	Reverse	AAGTGCTAGCTGGTTGTGCT
AURKB	Forward	CGATCATGGAGGAGTTGGCA
	Reverse	GGGGTTATGCCTGAGCAGTT
CANX	Forward	CCGGGAGGCTAGAGATCATGG
	Reverse	CAGGGGCATCTTCATCCCAG
CASP9	Forward	CAGGCCCCATATGATCGAGG
	Reverse	TCGACAACTTTGCTGCTTGC
DDIT4	Forward	CTGTCCTCACCATGCCTAGC
	Reverse	ATCCAGGTAAGCCGTGTCTTC
EGR1	Forward	TGACCCGTTCGGATCCTTTC
	Reverse	GAGTGGTTTGGCTGGGGTAA
FOS	Forward	CAGACTACGAGGCGTCATCC
	Reverse	AGTTGGTCTGTCTCCGCTTG
GAPDH	Forward	TGGTATCGTGGAAGGACTCATGAC
	Reverse	ATGCCAGTGAGCTTCCCGTTCAGC
GCNT3	Forward	CACCAGAGACTGTGAGCACTTC
	Reverse	CATACACAGCTCGCAGTAGCC T
IGF2BP3	Forward	ACTGCACGGGAAACCCATAG
	Reverse	CCAGCACCTCCCACTGTAAAT
IL10	Forward	TACGGCGCTGTCATCGATTT
	Reverse	AAGGTTTCTCAAGGGGCTGG
MSH2	Forward	GGAGGTGAGGAGGTTTCGAC
	Reverse	TTAAGGGCTCTGACTGCTGC
PAM	Forward	TATTCCGGTGGTTGTCCTGC
	Reverse	CGTCTCGAGTTTGTGTTCAGA
PCNA	Forward	GCCAGAGCTCTTCCCTTACG
	Reverse	TAGCTGGTTTCGGCTTCAGG
PPARG	Forward	AGTCGTGGTACTTTACGCC
	Reverse	TCGTTAAAGGCTGACTCTCGT
PTEN	Forward	CGCCTCCTCTTCGTCTTTTCTA
	Reverse	GCTGTGGTGGGTTATGGTCTT
RASD1	Forward	CCCCTCTGCCCAATGAAACT
	Reverse	TGAAAACGTCTCCTGTGAGG
SERPINI1	Forward	GGAAACCGGTGCAAAACCTC
	Reverse	AGGCCTAAACTGCGACTTCC
VEGF	Forward	TTTCGTCCAACTTCTGGGCT
	Reverse	CGGCTTGTCACATTTTTCTTGT
XRCC4	Forward	AGCGGGCGTTTTGGAAGATA
	Reverse	GGCTTTTCCTTTTCTTGAAGCTG

Supplementary Table 2. Clinical characteristics of epithelial ovarian cancer patients

Characteristics	N(%)
**Patients**	56
**Median Age (Range)**	56 (35-85)
**Stage**	
IIIA	7 (12.5)
IIIB	9 (16.1)
IIIC	32 (57.1)
IV	8 (14.3)
**Histology**	
Serous	51 (91.1)
Endometriod	2 (3.6)
Other	3 (5.4)
**Grade**	
1	1 (1.8)
2	20 (35.7)
3	35 (62.5)
**Surgical Outcome (Stage III)**	
Optimal (<1cm)	13 (23.2)
Sub-optimal (≥1cm)	21 (37.5)
**Response to Initial Therapy**	
Complete Response	38 (67.9)
Partial Response	11 (19.6)
Progression Disease	4 (7.1)
Stable Disease	3 (5.4)
**Recurrences**	48 (85.7)
**Deaths**	36 (64.3)
**Survival (months)**	
Median DFS (Range)	16 (1-127)
Median OS (Range)	35 (2-127)

Supplementary Table 3. Genomic analysis. List of 152 differentially deregulated probes.

Fold Change	pval (LiMMA)	FDR (LiMMA)	ProbeID	Systematic Name	Description
-2.71	0.00025	0.066	**ADAMTS7**	NM_014272	ref|Homo sapiens ADAM metallopeptidase with thrombospondin type 1 motif, 7 (ADAMTS7), mRNA [NM_014272]
-2.06	0.00025	0.066	**AK123993**	AK123993	gb|Homo sapiens cDNA FLJ41999 fis, clone SPLEN2029683. [AK123993]
-1.90	0.00053	0.073	**AK124190**	AK124190	gb|Homo sapiens cDNA FLJ42196 fis, clone THYMU2033816. [AK124190]
2.05	0.00010	0.055	**AKAP12**	NM_005100	ref|Homo sapiens A kinase (PRKA) anchor protein 12 (AKAP12), transcript variant 1, mRNA [NM_005100]
1.66	0.00023	0.063	**AMACR**	NM_001167595	ref|Homo sapiens alpha-methylacyl-CoA racemase (AMACR), transcript variant 3, mRNA [NM_001167595]
-2.08	0.00011	0.055	**ANKRD11**	NM_001256182	ref|Homo sapiens ankyrin repeat domain 11 (ANKRD11), transcript variant 1, mRNA [NM_001256182]
1.60	0.00006	0.055	**APOBEC3B**	NM_004900	ref|Homo sapiens apolipoprotein B mRNA editing enzyme, catalytic polypeptide-like 3B (APOBEC3B), transcript variant 1, mRNA [NM_004900]
-1.88	0.00005	0.055	**APPL1**	NM_012096	ref|Homo sapiens adaptor protein, phosphotyrosine interaction, PH domain and leucine zipper containing 1 (APPL1), mRNA [NM_012096]
-1.72	0.00012	0.055	**ATP8**	ENST00000361851	ens|mitochondrially encoded ATP synthase 8 [Source:HGNC Symbol;Acc:HGNC:7415] [ENST00000361851]
1.78	0.00003	0.055	**AURKB**	NM_004217	ref|Homo sapiens aurora kinase B (AURKB), transcript variant 1, mRNA [NM_004217]
-1.87	0.00013	0.055	**BACE1**	NM_012104	ref|Homo sapiens beta-site APP-cleaving enzyme 1 (BACE1), transcript variant a, mRNA [NM_012104]
-1.57	0.00008	0.055	**C12orf65**	NM_152269	ref|Homo sapiens chromosome 12 open reading frame 65 (C12orf65), transcript variant 1, mRNA [NM_152269]
-1.66	0.00068	0.076	**C6orf89**	NM_152734	ref|Homo sapiens chromosome 6 open reading frame 89 (C6orf89), transcript variant 1, mRNA [NM_152734]
-1.54	0.00007	0.055	**CAMSAP2**	NM_001297707	ref|Homo sapiens calmodulin regulated spectrin-associated protein family, member 2 (CAMSAP2), transcript variant 1, mRNA [NM_001297707]
1.54	0.00015	0.058	**CDCA2**	NM_152562	ref|Homo sapiens cell division cycle associated 2 (CDCA2), mRNA [NM_152562]
-2.03	0.00004	0.055	**CDK11B**	NM_033487	ref|Homo sapiens cyclin-dependent kinase 11
-1.79	0.00079	0.077	**CELF1**	NM_198700	ref|Homo sapiens CUGBP, Elav-like family member 1 (CELF1), transcript variant 2, mRNA [NM_198700]
-1.53	0.00058	0.074	**CENPP**	NM_001012267	ref|Homo sapiens centromere protein P (CENPP), transcript variant 1, mRNA [NM_001012267]
-2.25	0.00009	0.055	**CHAC1**	NM_024111	ref|Homo sapiens ChaC glutathione-specific gamma-glutamylcyclotransferase 1 (CHAC1), transcript variant 1, mRNA [NM_024111]
1.54	0.00026	0.066	**CHGB**	NM_001819	ref|Homo sapiens chromogranin B (secretogranin 1) (CHGB), mRNA [NM_001819]
-1.80	0.00014	0.055	**CTNNAL1**	NM_003798	ref|Homo sapiens catenin (cadherin-associated protein), alpha-like 1 (CTNNAL1), transcript variant 1, mRNA [NM_003798]
-1.58	0.00070	0.076	**CXXC5**	NM_016463	ref|Homo sapiens CXXC finger protein 5 (CXXC5), mRNA [NM_016463]
-1.55	0.00017	0.059	**CYGB**	NM_134268	ref|Homo sapiens cytoglobin (CYGB), mRNA [NM_134268]
1.53	0.00009	0.055	**CYTL1**	NM_018659	ref|Homo sapiens cytokine-like 1 (CYTL1), mRNA [NM_018659]
1.53	0.00011	0.055	**CYTL1**	NM_018659	ref|Homo sapiens cytokine-like 1 (CYTL1), mRNA [NM_018659]
1.56	0.00009	0.055	**CYTL1**	NM_018659	ref|Homo sapiens cytokine-like 1 (CYTL1), mRNA [NM_018659]
1.57	0.00006	0.055	**CYTL1**	NM_018659	ref|Homo sapiens cytokine-like 1 (CYTL1), mRNA [NM_018659]
1.59	0.00011	0.055	**CYTL1**	NM_018659	ref|Homo sapiens cytokine-like 1 (CYTL1), mRNA [NM_018659]
1.57	0.00016	0.059	**CYTL1**	NM_018659	ref|Homo sapiens cytokine-like 1 (CYTL1), mRNA [NM_018659]
1.51	0.00018	0.060	**CYTL1**	NM_018659	ref|Homo sapiens cytokine-like 1 (CYTL1), mRNA [NM_018659]
1.53	0.00045	0.073	**CYTL1**	NM_018659	ref|Homo sapiens cytokine-like 1 (CYTL1), mRNA [NM_018659]
-1.72	0.00014	0.055	**DDIT4**	NM_019058	ref|Homo sapiens DNA-damage-inducible transcript 4 (DDIT4), mRNA [NM_019058]
-1.73	0.00013	0.055	**DDIT4**	NM_019058	ref|Homo sapiens DNA-damage-inducible transcript 4 (DDIT4), mRNA [NM_019058]
-1.73	0.00014	0.057	**DDIT4**	NM_019058	ref|Homo sapiens DNA-damage-inducible transcript 4 (DDIT4), mRNA [NM_019058]

-1.72	0.00016	0.059	**DDIT4**	NM_019058	ref|Homo sapiens DNA-damage-inducible transcript 4 (DDIT4), mRNA [NM_019058]
-1.73	0.00021	0.061	**DDIT4**	NM_019058	ref|Homo sapiens DNA-damage-inducible transcript 4 (DDIT4), mRNA [NM_019058]
-1.73	0.00027	0.067	**DDIT4**	NM_019058	ref|Homo sapiens DNA-damage-inducible transcript 4 (DDIT4), mRNA [NM_019058]
-1.77	0.00041	0.073	**DDIT4**	NM_019058	ref|Homo sapiens DNA-damage-inducible transcript 4 (DDIT4), mRNA [NM_019058]
-1.67	0.00076	0.077	**DDIT4**	NM_019058	ref|Homo sapiens DNA-damage-inducible transcript 4 (DDIT4), mRNA [NM_019058]
-1.67	0.00087	0.079	**DDRGK1**	ENST00000380201	ens|DDRGK domain containing 1 [Source:HGNC Symbol;Acc:HGNC:16110] [ENST00000380201]
-1.69	0.00004	0.055	**DDX17**	NM_006386	ref|Homo sapiens DEAD (Asp-Glu-Ala-Asp) box helicase 17 (DDX17), transcript variant 1, mRNA [NM_006386]
1.65	0.00019	0.060	**DKK1**	NM_012242	ref|Homo sapiens dickkopf WNT signaling pathway inhibitor 1 (DKK1), mRNA [NM_012242]
-1.61	0.00040	0.073	**DRD5**	NM_000798	ref|Homo sapiens dopamine receptor D5 (DRD5), mRNA [NM_000798]
-1.95	0.00001	0.055	**EBLN3**	NR_036592	ref|Homo sapiens endogenous Bornavirus-like nucleoprotein 3 (EBLN3), non-coding RNA [NR_036592]
-1.79	0.00032	0.068	**EHF**	NM_012153	ref|Homo sapiens ets homologous factor (EHF), transcript variant 2, mRNA [NM_012153]
-1.68	0.00079	0.077	**EML4**	ENST00000409040	ens|echinoderm microtubule associated protein like 4 [Source:HGNC Symbol;Acc:HGNC:1316] [ENST00000409040]
-2.28	0.00050	0.073	**ENST00000414130**	ENST00000414130	ens|high mobility group AT-hook 1 pseudogene 8 (functional) [Source:HGNC Symbol;Acc:HGNC:39183] [ENST00000414130]
-1.92	0.00001	0.055	**EPS8**	NM_004447	ref|Homo sapiens epidermal growth factor receptor pathway substrate 8 (EPS8), mRNA [NM_004447]
1.69	0.00022	0.062	**FAM46C**	NM_017709	ref|Homo sapiens family with sequence similarity 46, member C (FAM46C), mRNA [NM_017709]
-2.00	0.00006	0.055	**FBRSL1**	NM_001142641	ref|Homo sapiens fibrosin-like 1 (FBRSL1), mRNA [NM_001142641]
2.14	0.00001	0.055	**FOS**	NM_005252	ref|Homo sapiens FBJ murine osteosarcoma viral oncogene homolog (FOS), mRNA [NM_005252]
-1.52	0.00004	0.055	**FUT1**	NM_000148	ref|Homo sapiens fucosyltransferase 1 (galactoside 2-alpha-L-fucosyltransferase, H blood group) (FUT1), mRNA [NM_000148]
-1.56	0.00008	0.055	**FUT1**	NM_000148	ref|Homo sapiens fucosyltransferase 1 (galactoside 2-alpha-L-fucosyltransferase, H blood group) (FUT1), mRNA [NM_000148]
-1.54	0.00031	0.068	**FUT1**	NM_000148	ref|Homo sapiens fucosyltransferase 1 (galactoside 2-alpha-L-fucosyltransferase, H blood group) (FUT1), mRNA [NM_000148]
-1.52	0.00034	0.070	**FUT1**	NM_000148	ref|Homo sapiens fucosyltransferase 1 (galactoside 2-alpha-L-fucosyltransferase, H blood group) (FUT1), mRNA [NM_000148]
-1.51	0.00053	0.073	**FUT1**	NM_000148	ref|Homo sapiens fucosyltransferase 1 (galactoside 2-alpha-L-fucosyltransferase, H blood group) (FUT1), mRNA [NM_000148]
-1.53	0.00047	0.073	**FUT1**	NM_000148	ref|Homo sapiens fucosyltransferase 1 (galactoside 2-alpha-L-fucosyltransferase, H blood group) (FUT1), mRNA [NM_000148]
-1.61	0.00080	0.077	**FUT1**	NM_000148	ref|Homo sapiens fucosyltransferase 1 (galactoside 2-alpha-L-fucosyltransferase, H blood group) (FUT1), mRNA [NM_000148]
-1.54	0.00082	0.078	**FUT1**	NM_000148	ref|Homo sapiens fucosyltransferase 1 (galactoside 2-alpha-L-fucosyltransferase, H blood group) (FUT1), mRNA [NM_000148]
-1.78	0.00008	0.055	**G0S2**	NM_015714	ref|Homo sapiens G0/G1 switch 2 (G0S2), mRNA [NM_015714]
-1.63	0.00017	0.059	**GOLGA6L4**	NM_001267536	ref|Homo sapiens golgin A6 family-like 4 (GOLGA6L4), mRNA [NM_001267536]
-1.62	0.00009	0.055	**HIST1H1E**	NM_005321	ref|Homo sapiens histone cluster 1, H1e (HIST1H1E), mRNA [NM_005321]
-1.81	0.00066	0.075	**HIST1H2AH**	NM_080596	ref|Homo sapiens histone cluster 1, H2ah (HIST1H2AH), mRNA [NM_080596]
-1.56	0.00003	0.055	**HIST1H2AJ**	NM_021066	ref|Homo sapiens histone cluster 1, H2aj (HIST1H2AJ), mRNA [NM_021066]
-1.70	0.00002	0.055	**HIST1H2AK**	NM_003510	ref|Homo sapiens histone cluster 1, H2ak (HIST1H2AK), mRNA [NM_003510]
-1.58	0.00005	0.055	**HIST1H2AM**	NM_003514	ref|Homo sapiens histone cluster 1, H2am (HIST1H2AM), mRNA [NM_003514]
-1.70	0.00053	0.073	**HIST1H3B**	NM_003537	ref|Homo sapiens histone cluster 1, H3b (HIST1H3B), mRNA [NM_003537]
-1.80	0.00011	0.055	**HIST1H3F**	NM_021018	ref|Homo sapiens histone cluster 1, H3f (HIST1H3F), mRNA [NM_021018]
-1.62	0.00011	0.055	**HIST1H3H**	NM_003536	ref|Homo sapiens histone cluster 1, H3h (HIST1H3H), mRNA [NM_003536]
-1.55	0.00027	0.067	**HIST1H4A**	NM_003538	ref|Homo sapiens histone cluster 1, H4a (HIST1H4A), mRNA [NM_003538]
-1.67	0.00006	0.055	**HIST1H4D**	NM_003539	ref|Homo sapiens histone cluster 1, H4d (HIST1H4D), mRNA [NM_003539]
-2.22	0.00052	0.073	**HIST1H4D**	NM_003539	ref|Homo sapiens histone cluster 1, H4d (HIST1H4D), mRNA [NM_003539]
-1.76	0.00010	0.055	**HIST1H4E**	NM_003545	ref|Homo sapiens histone cluster 1, H4e (HIST1H4E), mRNA [NM_003545]
-1.54	0.00029	0.068	**HIST2H2AC**	NM_003517	ref|Homo sapiens histone cluster 2, H2ac (HIST2H2AC), mRNA [NM_003517]
-1.55	0.00033	0.069	**HIST2H4B**	NM_001034077	ref|Homo sapiens histone cluster 2, H4b (HIST2H4B), mRNA [NM_001034077]
-1.62	0.00025	0.066	**HP1BP3**	NM_016287	ref|Homo sapiens heterochromatin protein 1, binding protein 3 (HP1BP3), mRNA [NM_016287]

-1.59	0.00058	0.074	**IGF2BP1**	NM_006546	ref|Homo sapiens insulin-like growth factor 2 mRNA binding protein 1 (IGF2BP1), transcript variant 1, mRNA [NM_006546]
-1.86	0.00003	0.055	**IGF2BP3**	NM_006547	ref|Homo sapiens insulin-like growth factor 2 mRNA binding protein 3 (IGF2BP3), mRNA [NM_006547]
-1.86	0.00005	0.055	**KRTAP3-3**	NM_033185	ref|Homo sapiens keratin associated protein 3-3 (KRTAP3-3), mRNA [NM_033185]
-1.79	0.00009	0.055	**LARS**	NM_020117	ref|Homo sapiens leucyl-tRNA synthetase (LARS), mRNA [NM_020117]
-2.31	0.00051	0.073	**LINC01001**	NR_028326	ref|Homo sapiens long intergenic non-protein coding RNA 1001 (LINC01001), long non-coding RNA [NR_028326]
-1.71	0.00002	0.055	**LOC285178**	AK091571	gb|Homo sapiens cDNA FLJ34252 fis, clone FCBBF5000061. [AK091571]
1.58	0.00007	0.055	**LRRC42**	NM_052940	ref|Homo sapiens leucine rich repeat containing 42 (LRRC42), transcript variant 2, mRNA [NM_052940]
-1.81	0.00017	0.059	**MIR4435-1HG**	NR_015395	ref|Homo sapiens MIR4435-1 host gene (non-protein coding) (MIR4435-1HG), transcript variant 1, long non-coding RNA [NR_015395]
-1.72	0.00008	0.055	**MYCBP2**	NM_015057	ref|Homo sapiens MYC binding protein 2, E3 ubiquitin protein ligase (MYCBP2), mRNA [NM_015057]
-1.62	0.00081	0.078	**NBPF10**	NM_001039703	ref|Homo sapiens neuroblastoma breakpoint family, member 10 (NBPF10), transcript variant 2, mRNA [NM_001039703]
-1.76	0.00001	0.055	**NBPF9**	CR936830	gb|Homo sapiens mRNA; cDNA DKFZp686H059 (from clone DKFZp686H059). [CR936830]
-2.17	0.00003	0.055	**ND4L**	ENST00000361335	ens|mitochondrially encoded NADH dehydrogenase 4L [Source:HGNC Symbol;Acc:HGNC:7460] [ENST00000361335]
-1.65	0.00003	0.055	**ODF2L**	NM_001007022	ref|Homo sapiens outer dense fiber of sperm tails 2-like (ODF2L), transcript variant 2, mRNA [NM_001007022]
1.53	0.00014	0.055	**PAM**	NM_001177306	ref|Homo sapiens peptidylglycine alpha-amidating monooxygenase (PAM), transcript variant 5, mRNA [NM_001177306]
-1.91	0.00014	0.055	**PGM5P2**	NR_002836	ref|Homo sapiens phosphoglucomutase 5 pseudogene 2 (PGM5P2), non-coding RNA [NR_002836]
-2.10	0.00005	0.055	**PKD1**	NM_000296	ref|Homo sapiens polycystic kidney disease 1 (autosomal dominant) (PKD1), transcript variant 2, mRNA [NM_000296]
-1.54	0.00052	0.073	**PNPLA3**	NM_025225	ref|Homo sapiens patatin-like phospholipase domain containing 3 (PNPLA3), mRNA [NM_025225]
1.51	0.00043	0.073	**POLR1C**	NM_203290	ref|Homo sapiens polymerase (RNA) I polypeptide C, 30kDa (POLR1C), mRNA [NM_203290]
-1.58	0.00004	0.055	**PREPL**	NM_006036	ref|Homo sapiens prolyl endopeptidase-like (PREPL), transcript variant 1, mRNA [NM_006036]
-1.53	0.00014	0.057	**PTP4A2**	NM_080391	ref|Homo sapiens protein tyrosine phosphatase type IVA, member 2 (PTP4A2), transcript variant 1, mRNA [NM_080391]
1.98	0.00001	0.055	**RASD1**	NM_016084	ref|Homo sapiens RAS, dexamethasone-induced 1 (RASD1), transcript variant 1, mRNA [NM_016084]
1.99	0.00001	0.055	**RASD1**	NM_016084	ref|Homo sapiens RAS, dexamethasone-induced 1 (RASD1), transcript variant 1, mRNA [NM_016084]
2.01	0.00001	0.055	**RASD1**	NM_016084	ref|Homo sapiens RAS, dexamethasone-induced 1 (RASD1), transcript variant 1, mRNA [NM_016084]
2.01	0.00002	0.055	**RASD1**	NM_016084	ref|Homo sapiens RAS, dexamethasone-induced 1 (RASD1), transcript variant 1, mRNA [NM_016084]
2.02	0.00008	0.055	**RASD1**	NM_016084	ref|Homo sapiens RAS, dexamethasone-induced 1 (RASD1), transcript variant 1, mRNA [NM_016084]
2.04	0.00006	0.055	**RASD1**	NM_016084	ref|Homo sapiens RAS, dexamethasone-induced 1 (RASD1), transcript variant 1, mRNA [NM_016084]
2.06	0.00012	0.055	**RASD1**	NM_016084	ref|Homo sapiens RAS, dexamethasone-induced 1 (RASD1), transcript variant 1, mRNA [NM_016084]
2.09	0.00003	0.055	**RASD1**	NM_016084	ref|Homo sapiens RAS, dexamethasone-induced 1 (RASD1), transcript variant 1, mRNA [NM_016084]
2.10	0.00003	0.055	**RASD1**	NM_016084	ref|Homo sapiens RAS, dexamethasone-induced 1 (RASD1), transcript variant 1, mRNA [NM_016084]
2.14	0.00002	0.055	**RASD1**	NM_016084	ref|Homo sapiens RAS, dexamethasone-induced 1 (RASD1), transcript variant 1, mRNA [NM_016084]
-2.04	0.00036	0.071	**RMRP**	NR_003051	ref|Homo sapiens RNA component of mitochondrial RNA processing endoribonuclease (RMRP), RNase MRP RNA [NR_003051]
-3.03	0.00044	0.073	**RN7SL1**	NR_002715	ref|Homo sapiens RNA, 7SL, cytoplasmic 1 (RN7SL1), SRP RNA [NR_002715]
-1.96	0.00009	0.055	**RNU2-1**	NR_002716	ref|Homo sapiens RNA, U2 small nuclear 1 (RNU2-1), small nuclear RNA [NR_002716]
-1.62	0.00075	0.077	**RPL13P5**	NR_002803	ref|Homo sapiens ribosomal protein L13 pseudogene 5 (RPL13P5), non-coding RNA [NR_002803]
-1.55	0.00025	0.066	**SATB1**	NM_002971	ref|Homo sapiens SATB homeobox 1 (SATB1), transcript variant 1, mRNA [NM_002971]
1.70	0.00056	0.073	**SERPINI1**	NM_005025	ref|Homo sapiens serpin peptidase inhibitor, clade I (neuroserpin), member 1 (SERPINI1), transcript variant 1, mRNA [NM_005025]
-1.63	0.00013	0.055	**SGSM2**	ENST00000574857	ens|small G protein signaling modulator 2 [Source:HGNC Symbol;Acc:HGNC:29026] [ENST00000574857]

-1.78	0.00028	0.068	**SIKE1**	NM_001102396	ref|Homo sapiens suppressor of IKBKE 1 (SIKE1), transcript variant 1, mRNA [NM_001102396]
-1.53	0.00004	0.055	**SLC25A16**	BC001407	gb|Homo sapiens solute carrier family 25 (mitochondrial carrier; Graves disease autoantigen), member 16, mRNA (cDNA clone IMAGE:3139311), with apparent retained intron. [BC001407]
-1.51	0.00005	0.055	**SLC39A5**	NM_001135195	ref|Homo sapiens solute carrier family 39 (zinc transporter), member 5 (SLC39A5), transcript variant 2, mRNA [NM_001135195]
1.58	0.00039	0.071	**SLC5A3**	NM_006933	ref|Homo sapiens solute carrier family 5 (sodium/myo-inositol cotransporter), member 3 (SLC5A3), mRNA [NM_006933]
-1.60	0.00087	0.079	**SLX4IP**	NM_001009608	ref|Homo sapiens SLX4 interacting protein (SLX4IP), mRNA [NM_001009608]
-1.56	0.00013	0.055	**SMA4**	NR_024054	ref|Homo sapiens glucuronidase, beta pseudogene (SMA4), transcript variant 2, non-coding RNA [NR_024054]
-1.78	0.00077	0.077	**SMC1A**	NM_001281463	ref|Homo sapiens structural maintenance of chromosomes 1A (SMC1A), transcript variant 2, mRNA [NM_001281463]
-2.57	0.00084	0.078	**SNORA28**	NR_002964	ref|Homo sapiens small nucleolar RNA, H/ACA box 28 (SNORA28), small nucleolar RNA [NR_002964]
-2.51	0.00079	0.077	**SNORA73B**	NR_004404	ref|Homo sapiens small nucleolar RNA, H/ACA box 73B (SNORA73B), small nucleolar RNA [NR_004404]
-1.56	0.00007	0.055	**SNORA74A**	NR_002915	ref|Homo sapiens small nucleolar RNA, H/ACA box 74A (SNORA74A), small nucleolar RNA [NR_002915]
-1.72	0.00014	0.055	**SPATA13**	NM_153023	ref|Homo sapiens spermatogenesis associated 13 (SPATA13), transcript variant 2, mRNA [NM_153023]
-1.68	0.00032	0.068	**SRP72**	NM_006947	ref|Homo sapiens signal recognition particle 72kDa (SRP72), transcript variant 1, mRNA [NM_006947]
-1.67	0.00068	0.076	**SRSF11**	NM_001190987	ref|Homo sapiens serine/arginine-rich splicing factor 11 (SRSF11), transcript variant 2, mRNA [NM_001190987]
-1.86	0.00001	0.055	**SSB**	NM_003142	ref|Homo sapiens Sjogren syndrome antigen B (autoantigen La) (SSB), transcript variant 1, mRNA [NM_003142]
-1.92	0.00018	0.060	**SYT1**	NM_005639	ref|Homo sapiens synaptotagmin I (SYT1), transcript variant 1, mRNA [NM_005639]
1.63	0.00032	0.068	**SYT13**	NM_020826	ref|Homo sapiens synaptotagmin XIII (SYT13), transcript variant 1, mRNA [NM_020826]
-1.54	0.00003	0.055	**TBL1X**	NM_005647	ref|Homo sapiens transducin (beta)-like 1X-linked (TBL1X), transcript variant 1, mRNA [NM_005647]
-1.81	0.00048	0.073	**TCF25**	NM_014972	ref|Homo sapiens transcription factor 25 (basic helix-loop-helix) (TCF25), mRNA [NM_014972]
-1.79	0.00011	0.055	**TINAGL1**	NM_022164	ref|Homo sapiens tubulointerstitial nephritis antigen-like 1 (TINAGL1), transcript variant 1, mRNA [NM_022164]
-1.59	0.00070	0.076	**TMEM125**	NM_144626	ref|Homo sapiens transmembrane protein 125 (TMEM125), mRNA [NM_144626]
1.89	0.00004	0.055	**TNFAIP2**	NM_006291	ref|Homo sapiens tumor necrosis factor, alpha-induced protein 2 (TNFAIP2), mRNA [NM_006291]
-2.36	0.00029	0.068	**TRMT61A**	NM_152307	ref|Homo sapiens tRNA methyltransferase 61A (TRMT61A), mRNA [NM_152307]
-1.62	0.00007	0.055	**TXLNG**	NM_018360	ref|Homo sapiens taxilin gamma (TXLNG), transcript variant 1, mRNA [NM_018360]
-1.54	0.00048	0.073	**UBE2O**	NM_022066	ref|Homo sapiens ubiquitin-conjugating enzyme E2O (UBE2O), mRNA [NM_022066]
-1.63	0.00045	0.073	**UCP3**	NM_022803	ref|Homo sapiens uncoupling protein 3 (mitochondrial, proton carrier) (UCP3), transcript variant short, mRNA [NM_022803]
-1.66	0.00003	0.055	**UPF2**	NM_080599	ref|Homo sapiens UPF2 regulator of nonsense transcripts homolog (yeast) (UPF2), transcript variant 1, mRNA [NM_080599]
-1.64	0.00011	0.055	**USP16**	NM_001001992	ref|Homo sapiens ubiquitin specific peptidase 16 (USP16), transcript variant 2, mRNA [NM_001001992]
-2.03	0.00016	0.059	**USP42**	NM_032172	ref|Homo sapiens ubiquitin specific peptidase 42 (USP42), mRNA [NM_032172]
-1.52	0.00046	0.073	**UTP23**	NM_032334	ref|Homo sapiens UTP23, small subunit (SSU) processome component, homolog (yeast) (UTP23), mRNA [NM_032334]
-1.67	0.00085	0.079	**VAPB**	NM_004738	ref|Homo sapiens VAMP (vesicle-associated membrane protein)-associated protein B and C (VAPB), transcript variant 1, mRNA [NM_004738]
-1.64	0.00065	0.075	**VEGFA**	NM_001025370	ref|Homo sapiens vascular endothelial growth factor A (VEGFA), transcript variant 6, mRNA [NM_001025370]
-1.82	0.00018	0.060	**XLOC_l2_013383**	TCONS_l2_00025857	linc|BROAD Institute lincRNA (XLOC_l2_013383), lincRNA [TCONS_l2_00025857]
-1.61	0.00059	0.074	**ZKSCAN1**	NM_001287054	ref|Homo sapiens zinc finger with KRAB and SCAN domains 1 (ZKSCAN1), transcript variant 2, mRNA [NM_001287054]
-1.66	0.00002	0.055	**ZNF587**	NM_032828	ref|Homo sapiens zinc finger protein 587 (ZNF587), transcript variant 1, mRNA [NM_032828]
-1.72	0.00005	0.055	**ZNF587**	NM_032828	ref|Homo sapiens zinc finger protein 587 (ZNF587), transcript variant 1, mRNA [NM_032828]

-1.69	0.00018	0.060	**ZNF587**	NM_032828	ref|Homo sapiens zinc finger protein 587 (ZNF587), transcript variant 1, mRNA [NM_032828]
-1.75	0.00028	0.068	**ZNF587**	NM_032828	ref|Homo sapiens zinc finger protein 587 (ZNF587), transcript variant 1, mRNA [NM_032828]
-1.68	0.00055	0.073	**ZNF587**	NM_032828	ref|Homo sapiens zinc finger protein 587 (ZNF587), transcript variant 1, mRNA [NM_032828]
-1.80	0.00084	0.078	**ZNF587**	NM_032828	ref|Homo sapiens zinc finger protein 587 (ZNF587), transcript variant 1, mRNA [NM_032828]

Supplementary Table 4. Gene ontology (GO) terms obtained from transcriptomic study of GCNT3.

Items	Items_Details	Support	List size	Reference Support	Reference size	Hyp	Hyp_c	Genes
GO:0000278	mitotic cell cycle (BP)	34	751	304	34208	9.69E-15	1.75E-11	CDC20,MAPRE1,CENPP,BUB1,KIF23,ERCC6L,CCDC99,PSMD10,CDKN2C,AURKB,UBB,PLK1,PSMD6,SMC1A,RCC2,CCNB1,NINL,CENPF,CCNA2,RPA3,CDK5RAP2,GINS2,NUF2,PCNA,TYMS,ANAPC1,CCNB2,STAG2,OFD1,CENPA,NDC80,ORC6,KIF20A,NEDD1
GO:0000087	M phase of mitotic cell cycle (BP)	18	751	95	34208	2.63E-12	1.59E-09	CDC20,MAPRE1,CENPP,BUB1,KIF23,ERCC6L,CCDC99,AURKB,PLK1,SMC1A,RCC2,CENPF,DLGAP5,NUF2,STAG2,CENPA,NDC80,KIF20A
GO:0000122	negative regulation of transcription from RNA polymerase II promoter (BP)	36	751	416	34208	3.72E-12	1.68E-09	HSBP1,SATB1,RPS14,PPARG,LEF1,TBL1X,PSMD10,AURKB,EGR1,TBL1XR1,EFNA1,SETD8,PLK1,ASCL2,DKK1,FOXM1,C19orf2,PAWR,EPC1,NR6A1,TCF7L2,PCNA,VAX2,TCF25,NFIC,DRAP1,MDFI,RXRA,TAF3,PKIG,HOXA7,DNMT3A,MSX1,ZNF148,MXI1,FOSB
GO:0051301	cell division (BP)	30	751	286	34208	1.93E-12	1.74E-09	CDC20,MAPRE1,CDCA3,BUB1,ERCC6L,NEK4,CCDC99,CDCA2,SETD8,USP16,PSRC1,SMC1A,CDC40,RCC2,CCNB1,MAEA,CENPF,KIFC1,CCNA2,NUF2,ANAPC1,CCNB2,STAG2,ASPM,NDC80,CDK11A,NEDD1,SMC4,NEDD9,MAU2
GO:0000236	mitotic prometaphase (BP)	16	751	85	34208	4.79E-11	1.73E-08	CDC20,MAPRE1,CENPP,BUB1,ERCC6L,CCDC99,AURKB,PLK1,SMC1A,RCC2,CCNB1,CENPF,NUF2,STAG2,CENPA,NDC80
GO:0007067	mitosis (BP)	21	751	187	34208	1.16E-09	3.48E-07	CDCA3,KIF23,NEK4,EML4,CDCA2,SETD8,PLK1,USP16,PSRC1,CCNA2,NUF2,ANAPC1,CCNB2,OFD1,ASPM,NDC80,ANLN,CDK11A,NEDD1,NEDD9,MAU2
GO:0045944	positive regulation of transcription from RNA polymerase II promoter (BP)	36	751	578	34208	2.84E-08	7.34E-06	FOS,SMARCB1,PPARG,LEF1,VEGFA,KLF6,TBL1X,NFYA,EGR1,TBL1XR1,EVX1,FSTL3,LMO4,CHD8,PHIP,FOXL2,CYTL1,BCL11A,FOXM1,RNF10,TLR4,EPC1,IL10,TCF7L2,SIX1,NFIC,TBL1Y,RXRA,RHOQ,BCL9L,HOXA7,MSX1,ZNF148,THRAP3,PLAGL2,HIF3A
GO:0006334	nucleosome assembly (BP)	14	751	102	34208	5.36E-08	1.21E-05	CENPP,HIST1H1E,HIST1H2AD,HIST2H2AB,HIST2H2AC,HIST1H2AH,HIST1H1C,HP1BP3,HIST1H2BN,HIST1H2AJ,CENPA,HIST1H4A,HIST1H2BB,HIST1H2AC
GO:0016070	RNA metabolic process (BP)	22	751	257	34208	7.05E-08	1.42E-05	ZFP36L1,RPS14,EDC4,RPS29,UPF3A,PSMD10,UBB,RPL37A,RPL28,EIF4G2,PABPC1,SMG1,PSMD6,UPF2,RPL31,EIF4A1,XRN2,RPL39,SNUPN,LENG9,YWHAB,DDX6
GO:0008283	cell proliferation (BP)	24	751	312	34208	1.37E-07	2.47E-05	MAPRE1,EHF,EMP1,CDK9,BUB1,PRDM4,INSIG1,AURKB,PTEN,PLK1,SRRT,CDK5R1,CENPF,APPL1,DLGAP5,NR6A1,STIL,TCF7L2,PCNA,EPS8,PIM2,MET,CDK11A,CHRM3
GO:0042493	response to drug (BP)	23	751	301	34208	2.79E-07	4.58E-05	FYN,ARG2,FOS,PPARG,NES,PRSS8,ABCB1,SRP72,CPT1A,SMPD1,PTK2B,CCNB1,CENPF,IL10,MSH2,TYMS,TOP2A,PAM,GNAS,MET,CST3,PLIN2,PDGFA
GO:0006412	translation (BP)	20	751	241	34208	4.52E-07	6.80E-05	RPS14,MRPS7,RPS29,MRPS33,RPL37A,RPL28,EIF4G2,PABPC1,MRPS24,MRPS6,MRPS23,EEF1A1,RPL22L1,RPL31,EIF4A1,RPL39,RRBP1,EIF4E2,IGF2BP3,EIF3A
GO:0045893	positive regulation of transcription, DNA-dependent (BP)	29	751	468	34208	6.99E-07	9.71E-05	FOS,PPARG,EHF,LEF1,KLF6,TBL1X,EGR1,TBL1XR1,USP16,CHD8,PHIP,PSRC1,FOXL2,CCNA2,FOXM1,AFAP1L2,TAF15,RNF10,ZSCAN21,CDK5RAP2,EPC1,IL10,MED4,OVOL2,SIX1,PIM2,PLCB1,ANKRD49,THRAP3
GO:0000086	G2/M transition of mitotic cell cycle (BP)	13	751	115	34208	1.55E-06	2.00E-04	MAPRE1,NES,ABCB1,PLK1,CCNB1,NINL,CCNA2,FOXM1,CDK5RAP2,CCNB2,OFD1,PLCB1,NEDD1

GO:0016071	mRNA metabolic process (BP)	18	751	223	34208	2.50E-06	2.82E-04	ZFP36L1,RPS14,EDC4,RPS29,UPF3A,PSMD10,UBB,RPL37A,RPL28,PABPC1,SMG1,PSMD6,UPF2,RPL31,EIF4A1,RPL39,YWHAB,DDX6
GO:0008285	negative regulation of cell proliferation (BP)	23	751	341	34208	2.40E-06	2.90E-04	SMARCB1,PPARG,FTH1,CDKN2C,PTEN,PTK2B,SPRY2,BTG2,PAWR,ASPH,LEPREL2,STK4,SPRY1,OVOL2,TENC1,PIM2,TBRG1,RXRA,CAV2,HSF1,MSX1,PPAP2A,MXI1
GO:0007049	cell cycle (BP)	26	751	435	34208	4.99E-06	5.00E-04	CDC20,SMARCB1,CDCA3,CDK9,NEK4,DMTF1,AURKB,CDCA2,SETD8,ATR,USP16,PSRC1,G0S2,MAEA,APPL1,KIFC1,FOXM1,DLGAP5,PIM2,ASPM,TBRG1,NDC80,ANLN,SMC4,NEDD9,MAU2
GO:0010467	gene expression (BP)	25	751	408	34208	4.91E-06	5.21E-04	RPS14,PPARG,PPA2,CDK9,RPS29,RPL37A,RPL28,FARS2,PABPC1,SRSF7,SF3B3,SMC1A,CDC40,EEF1A1,RPL31,SRSF11,EIF4A1,NR6A1,RPL39,MED4,LARS,NUDT21,RXRA,EIF3A,IARS
GO:0006397	mRNA processing (BP)	16	751	202	34208	1.13E-05	1.07E-03	RNPC3,SPOP,LGALS3,HNRNPA1L2,ROD1,ESRP1,A1CF,SRSF7,PDCD11,SF3B3,CDK13,DHX15,SRSF11,XRN2,CELF1,NUDT21
GO:0006355	regulation of transcription, DNA-dependent (BP)	62	751	1609	34208	1.46E-05	1.32E-03	ZFP36L1,CDK9,PRDM4,KLF6,GTF2H5,DMTF1,NFYA,SF1,EVX1,ASCL2,LMO4,ZNF618,ZNF280D,ZMYM2,ZNF226,ZNF770,KRBA2,ASCC3,FOXL2,ZNF587,BCL11A,EEF1A1,PDE8B,ZNF525,BTG2,TGFBRAP1,FOXM1,CCNL1,XRN2,PAWR,ZSCAN21,LEO1,ZNF329,TXLNG,NFATC2IP,VAX2,CCNL2,TOP2A,ZKSCAN1,FOXN2,SIX1,KLHL31,NFIC,CHD9,ZNF720,MYCBP2,WWC1,RFX1,TAF3,MYT1,CEBPB,PASK,BCL9L,HSF1,ZNF658,ZBTB8A,CDK11A,PLAGL2,HIF3A,ARID3A,MXI1,BBX
GO:0006917	induction of apoptosis (BP)	15	751	185	34208	1.60E-05	1.38E-03	NUDT2,PPARG,LEF1,CDKN2C,PTEN,FOXL2,BNIP3L,PAWR,TP53BP2,TNFSF10,STK4,PDCD7,PMAIP1,CEBPB,PLAGL2
GO:0060325	face morphogenesis (BP)	6	751	26	34208	1.74E-05	1.43E-03	LEF1,DKK1,PLEKHA1,RAB3GAP1,ASPH,MSX1
GO:0006281	DNA repair (BP)	19	751	285	34208	2.08E-05	1.64E-03	EEPD1,NUDT1,UBB,ATR,SMG1,SMC1A,BTG2,FOXM1,RPA3,RAD51AP1,MSH2,PCNA,TYMS,SUMO1,TOP2A,POLQ,XRCC4,NSMCE2,SMC4
GO:0006915	apoptotic process (BP)	30	751	594	34208	2.58E-05	1.79E-03	ARG2,PLEC,SH3KBP1,RABEP1,SATB1,BUB1,TJP2,CASP9,NET1,PSMD10,UBB,PSMD6,PTK2B,APPL1,PAWR,ARHGEF3,TP53BP2,BCL7C,TNFSF10,PDCD7,DDIT4,FASTK,LMNA,TNS4,YWHAB,PMAIP1,GSN,RNF130,CST3,CDK11A
GO:0000079	regulation of cyclin-dependent protein kinase activity (BP)	8	751	55	34208	2.55E-05	1.84E-03	CDKN2C,PTEN,CCNB1,CDK5R1,CCNA2,CCNL1,CCNL2,CCNB2
GO:0001578	microtubule bundle formation (BP)	5	751	17	34208	2.50E-05	1.88E-03	PLK1,NAV1,PSRC1,CDK5RAP2,KIF20A
GO:0045892	negative regulation of transcription, DNA-dependent (BP)	23	751	401	34208	3.29E-05	2.20E-03	PPARG,LEF1,YWHAQ,TBL1X,TBL1XR1,SETD8,CHD8,FOXL2,TRIM66,CDK5R1,CENPF,FOXM1,EPC1,TCF7L2,SUMO1,OVOL2,YWHAB,TBL1Y,RASD1,PLCB1,CEBPB,HOXA7,ZNF148
GO:0043066	negative regulation of apoptotic process (BP)	18	751	272	34208	3.75E-05	2.42E-03	NES,LEF1,VEGFA,PSMD10,PTEN,PLK1,PTK2B,CHD8,PHIP,PLAUR,SPRY2,TSC22D1,BTG2,BNIP3L,STIL,PLK2,MSX1,PRDX3
GO:0006605	protein targeting (BP)	6	751	30	34208	4.16E-05	2.59E-03	YWHAQ,TOMM20L,AKAP12,SYNGR1,YWHAB,HOMER3
GO:0006260	DNA replication (BP)	12	751	152	34208	1.42E-04	8.29E-03	ATR,CENPF,RPA3,GINS2,PCNA,TYMS,TOP2A,POLQ,RBMS1,NFIC,TBRG1,ORC6
GO:0000132	establishment of mitotic spindle	4	751	13	34208	1.41E-04	8.47E-03	CCDC99,CDK5RAP2,CENPA,NDC80

	orientation (BP)							
GO:0000075	cell cycle checkpoint (BP)	11	751	131	34208	1.55E-04	8.76E-03	CDC20,PSMD10,UBB,ATR,PSMD6,SMC1A,CCNB1,RPA3,ANAPC1,CCNB2,ORC6
GO:0001701	in utero embryonic development (BP)	13	751	179	34208	1.76E-04	9.65E-03	VEGFA,ASCL2,ANKRD11,CHD8,CCNB1,PKD1,ATP7A,STIL,TPM1,MSH2,CCNB2,XRCC4,MSX1
GO:0048015	phosphatidylinositol-mediated signaling (BP)	8	751	73	34208	1.99E-04	1.06E-02	CASP9,PTEN,PTAFR,PCNA,TYMS,TOP2A,NDC80,GSN
GO:0044267	cellular protein metabolic process (BP)	17	751	284	34208	2.05E-04	1.06E-02	PFDN6,RPS14,RPS29,RPL37A,TUBB6,RPL28,ALG11,PABPC1,CCT6A,PLAUR,EEF1A1,RPL31,EIF4A1,RPL39,UGGT1,CANX,EIF3A
GO:0000910	cytokinesis (BP)	7	751	56	34208	2.18E-04	1.09E-02	KIF23,AURKB,PLK1,ANLN,KIF20A,ROCK2,STX2
GO:0030518	intracellular steroid hormone receptor signaling pathway (BP)	4	751	15	34208	2.59E-04	1.23E-02	GPER,MED4,THRAP3,PPAP2A
GO:0000070	mitotic sister chromatid segregation (BP)	4	751	15	34208	2.59E-04	1.23E-02	SMC1A,KIFC1,NDC80,SMC4
GO:0008380	RNA splicing (BP)	16	751	267	34208	3.08E-04	1.36E-02	RNPC3,LGALS3,HNRNPA1L2,SF1,PPIG,PABPC1,SRSF7,SF3B3,SMC1A,CDC40,CDK13,DHX15,DDX5,SRSF11,PDCD7,NUDT21
GO:0030855	epithelial cell differentiation (BP)	6	751	42	34208	2.94E-04	1.36E-02	PPARG,EHF,VEGFA,F11R,SIX1,STX2
GO:0006366	transcription from RNA polymerase II promoter (BP)	17	751	294	34208	3.07E-04	1.39E-02	FOS,DEAF1,CDK9,PRDM4,NFYA,EGR1,LMO4,SRSF7,CDC40,TSC22D1,FOXM1,SRSF11,MED4,NFIC,NUDT21,CEBPB,ZNF148
GO:0007052	mitotic spindle organization (BP)	4	751	16	34208	3.40E-04	1.46E-02	KIF3B,SMC1A,PLK2,NDC80
GO:0000082	G1/S transition of mitotic cell cycle (BP)	11	751	145	34208	3.76E-04	1.54E-02	PSMD10,CDKN2C,UBB,PSMD6,CCNB1,RPA3,PCNA,TYMS,PIM2,PLK2,ORC6
GO:0031145	anaphase-promoting complex-dependent proteasomal ubiquitin-dependent protein catabolic process (BP)	8	751	80	34208	3.75E-04	1.57E-02	CDC20,PSMD10,AURKB,UBB,PLK1,PSMD6,CCNB1,ANAPC1
GO:0016032	viral reproduction (BP)	18	751	329	34208	4.03E-04	1.62E-02	FYN,RPS14,CDK9,SLC25A4,RPS29,PSMD10,UBB,RPL37A,RPL28,PSMD6,RPL31,ZSCAN21,RPL39,ZKSCAN1,B2M,XRCC4,POM121,NUPL2
GO:0021873	forebrain neuroblast division (BP)	2	751	2	34208	4.81E-04	1.81E-02	LEF1,ASPM
GO:0048069	eye pigmentation (BP)	2	751	2	34208	4.81E-04	1.81E-02	LEF1,HPS1
GO:0007092	activation of mitotic anaphase-promoting complex activity (BP)	2	751	2	34208	4.81E-04	1.81E-02	PTEN,PLK1

GO:0001666	response to hypoxia (BP)	12	751	175	34208	5.20E-04	1.92E-02	ARG2,VEGFA,ASCL2,PTK2B,TLR4,DDIT4,UCP3,PAM,TFRC,CST3,HIF3A,PDGFA
GO:0006921	cellular component disassembly involved in apoptosis (BP)	6	751	49	34208	6.87E-04	2.48E-02	PLEC,SATB1,TJP2,LMNA,PMAIP1,GSN
GO:0014070	response to organic cyclic compound (BP)	9	751	112	34208	8.32E-04	2.55E-02	FOS,LEF1,CPT1A,BTG2,MSH2,RXRA,MET,CST3,PLIN2
GO:0007417	central nervous system development (BP)	9	751	112	34208	8.32E-04	2.55E-02	NES,PTEN,ASCL2,TP53BP2,STK4,PAM,XRCC4,SERPINI1,FMR1
GO:0006457	protein folding (BP)	12	751	182	34208	7.36E-04	2.61E-02	PFDN6,DNAJC19,PPID,DNAJC21,PPIG,TUBB6,CCT6A,FKBP2,C19orf2,FKBP9,UGGT1,CANX
GO:0008360	regulation of cell shape (BP)	8	751	90	34208	8.28E-04	2.62E-02	FYN,SH3KBP1,VEGFA,MYH14,ARAP1,PTK2B,FMNL1,RHOQ
GO:0040007	growth (BP)	5	751	34	34208	8.26E-04	2.66E-02	SEPP1,VEGFA,BMP8B,VPS54,CCNB2
GO:0043086	negative regulation of catalytic activity (BP)	5	751	34	34208	8.26E-04	2.66E-02	PDE6D,PCSK9,CAMK2N1,NOSIP,ANP32E
GO:0034614	cellular response to reactive oxygen species (BP)	3	751	9	34208	8.02E-04	2.68E-02	FOS,TPM1,PRDX3
GO:0043517	positive regulation of DNA damage response, signal transduction by p53 class mediator (BP)	3	751	9	34208	8.02E-04	2.68E-02	ATR,PMAIP1,MSX1
GO:0006119	oxidative phosphorylation (BP)	3	751	9	34208	8.02E-04	2.68E-02	UQCRH,UQCRB,MSH2
GO:0018105	peptidyl-serine phosphorylation (BP)	6	751	52	34208	9.47E-04	2.76E-02	PLK1,ATR,SMG1,CDK5R1,PKD1,STK4
GO:0007568	aging (BP)	9	751	114	34208	9.44E-04	2.79E-02	FOS,AURKB,TYMS,UCP3,TOP2A,CANX,RXRA,GSN,TFRC
GO:0051437	positive regulation of ubiquitin-protein ligase activity involved in mitotic cell cycle (BP)	7	751	71	34208	9.41E-04	2.83E-02	CDC20,PSMD10,UBB,PLK1,PSMD6,CCNB1,ANAPC1
GO:0050821	protein stabilization (BP)	6	751	53	34208	1.05E-03	3.00E-02	PTEN,A1CF,NAPG,PIM2,TBRG1,MSX1
GO:0007213	G-protein coupled acetylcholine receptor signaling pathway (BP)	3	751	10	34208	1.13E-03	3.18E-02	CDK5R1,PLCB1,CHRM3
GO:0051439	regulation of ubiquitin-protein ligase activity involved in mitotic cell cycle (BP)	7	751	75	34208	1.30E-03	3.62E-02	CDC20,PSMD10,UBB,PLK1,PSMD6,CCNB1,ANAPC1

GO:0006468	protein phosphorylation (BP)	19	751	398	34208	1.44E-03	3.77E-02	FYN,SGK1,CDK9,BUB1,NEK4,GMFG,PLK1,ATR,PTK2B,CDK13,CDK5R1,STK4,FASTK,PIM2,PLK2,PASK,HSF1,CDK11A,ROCK2
GO:0034261	negative regulation of Ras GTPase activity (BP)	2	751	3	34208	1.42E-03	3.78E-02	SPRY2,SPRY1
GO:0072089	stem cell proliferation (BP)	2	751	3	34208	1.42E-03	3.78E-02	NES,ABCB1
GO:0007080	mitotic metaphase plate congression (BP)	3	751	11	34208	1.52E-03	3.82E-02	CCDC99,PSRC1,CCNB1
GO:0046209	nitric oxide metabolic process (BP)	3	751	11	34208	1.52E-03	3.82E-02	CALM2,ZDHHC21,NOSIP
GO:0010269	response to selenium ion (BP)	3	751	11	34208	1.52E-03	3.82E-02	ARG2,SEPP1,RXRA
GO:0007173	epidermal growth factor receptor signaling pathway (BP)	7	751	76	34208	1.41E-03	3.85E-02	SH3KBP1,UBB,PTK2B,SPRY2,SPRY1,EPS8,YWHAB
GO:0007264	small GTPase mediated signal transduction (BP)	16	751	312	34208	1.62E-03	4.00E-02	RASL11B,PTPLAD1,SH2D3A,YWHAQ,NET1,RAB12,ARAP1,ARHGEF3,RAC3,ARL1,RAB30,YWHAB,RASD1,RHOQ,DEPDC1B,ARL4A
GO:0007059	chromosome segregation (BP)	6	751	59	34208	1.84E-03	4.49E-02	CENPF,CDK5RAP2,NUF2,TOP2A,NDC80,MAU2
GO:0009790	embryo development (BP)	9	751	128	34208	2.12E-03	4.73E-02	BUB1,CDK5R1,FOXM1,TPM1,FOXN2,CELF1,MDFI,ATP5A1,PDGFA
GO:0044255	cellular lipid metabolic process (BP)	9	751	128	34208	2.12E-03	4.73E-02	TBL1X,TBL1XR1,CPT1A,AMACR,CPT2,CHD9,RXRA,AGPS,PLIN2
GO:0043065	positive regulation of apoptotic process (BP)	11	751	178	34208	2.02E-03	4.74E-02	NET1,SMPD1,FOXL2,TSC22D1,PAWR,TLR4,STK4,TOP2A,RXRA,PMAIP1,MSX1
GO:0010800	positive regulation of peptidyl-threonine phosphorylation (BP)	3	751	12	34208	2.00E-03	4.75E-02	PLK1,PHIP,MET
GO:0015074	DNA integration (BP)	3	751	12	34208	2.00E-03	4.75E-02	SMARCB1,KRBA2,PPFIBP1
GO:0007275	multicellular organismal development (BP)	35	751	945	34208	2.06E-03	4.76E-02	FYN,TNFAIP2,TSNAX,DEAF1,LSM14B,EHF,EMP1,NES,VEGFA,HOXA9,BMP8B,EVX1,MMP19,ASCL2,DKK1,NAV1,ATR,CDK13,SPRY2,UNC45A,CENPF,LEPR,STIL,ITGB5,VAX2,SPRY1,DISC1,MET,MYT1,HOXA7,RNF114,HOOK1,MSX1,HOXA6,FOSB
GO:0001541	ovarian follicle development (BP)	5	751	42	34208	2.19E-03	4.82E-02	VEGFA,MMP19,FOXL2,LFNG,ERMP1
GO:0007420	brain development (BP)	11	751	179	34208	2.11E-03	4.83E-02	SEPP1,NES,LEF1,RAB3GAP1,CDK5R1,CDK5RAP2,ECE2,TCF7L2,TMEM57,MET,CST3
GO:0006351	transcription, DNA-dependent (BP)	8	751	105	34208	2.24E-03	4.87E-02	HOXA9,ASCL2,POLR3C,EPC1,SIX1,COL4A2,HOXA7,ZNF148
GO:0045040	protein import into	2	751	4	34208	2.80E-03	4.97E-02	HSPA4,SAMM50

	mitochondrial outer membrane (BP)							
GO:0060547	negative regulation of necrotic cell death (BP)	2	751	4	34208	2.80E-03	4.97E-02	SLC25A4,FTH1
GO:0045617	negative regulation of keratinocyte differentiation (BP)	2	751	4	34208	2.80E-03	4.97E-02	OVOL2,HOXA7
GO:0051256	spindle midzone assembly involved in mitosis (BP)	2	751	4	34208	2.80E-03	4.97E-02	KIF23,AURKB
GO:0010626	negative regulation of Schwann cell proliferation (BP)	2	751	4	34208	2.80E-03	4.97E-02	ASCL2,RNF10
GO:0071569	protein ufmylation (BP)	2	751	4	34208	2.80E-03	4.97E-02	UFM1,UFC1
GO:0006122	mitochondrial electron transport, ubiquinol to cytochrome c (BP)	2	751	4	34208	2.80E-03	4.97E-02	UQCRH,UQCRB
GO:0030488	tRNA methylation (BP)	2	751	4	34208	2.80E-03	4.97E-02	TRMT61A,ALKBH8
GO:0006508	proteolysis (BP)	23	751	543	34208	2.31E-03	4.97E-02	AMZ1,PREPL,DPEP3,USP42,CASP9,PRSS8,TBL1X,PRSS16,ADAMTS7,MMP19,USP16,PSMD6,PCSK9,ERMP1,ECE2,FBXW2,TINAGL1,BACE1,PRSS1,USP15,SEC11C,TFRC,SCRN2

Supplementary Table 5. Proteomic analysis. List of 135 statistically significant GCNT3 interactors.

Protein IDs	Protein names	Gene names	(-)Log p-value (G/N)	RATIO: G vs N
Q5T447	E3 ubiquitin-protein ligase HECTD3	*HECTD3*	8.82372	4.58871
Q8IWV8	E3 ubiquitin-protein ligase UBR2	*UBR2*	8.609	8.26826
O14874	[3-methyl-2-oxobutanoate dehydrogenase [lipoamide]] kinase, mitochondrial	*BCKDK*	7.90897	9.62341
O95395	Beta-1,3-galactosyl-O-glycosyl-glycoprotein beta-1,6-N-acetylglucosaminyltransferase 3	*GCNT3*	7.89244	16.2941
O43242	26S proteasome non-ATPase regulatory subunit 3	*PSMD3*	7.70338	3.14391
Q9UBV2	Protein sel-1 homolog 1	*SEL1L*	6.89164	6.65591
Q9Y4L1	Hypoxia up-regulated protein 1	*HYOU1*	6.70966	7.62646
Q9NYU2	UDP-glucose:glycoprotein glucosyltransferase 1	*UGGT1*	6.54258	8.43405
A0FGR8	Extended synaptotagmin-2	*ESYT2*	6.1027	6.5107
Q14CZ7	FAST kinase domain-containing protein 3	*FASTKD3*	6.05268	3.26691
P46821	Microtubule-associated protein 1B;MAP1B heavy chain;MAP1 light chain LC1	*MAP1B*	6.01712	3.73571
Q9NVI1	Fanconi anemia group I protein	*FANCI*	5.80338	4.03871
Q9H5Q4	Dimethyladenosine transferase 2, mitochondrial	*TFB2M*	5.64761	3.3497
P16615	Sarcoplasmic/endoplasmic reticulum calcium ATPase 2	*ATP2A2*	5.56566	9.15333
Q9H936	Mitochondrial glutamate carrier 1	*SLC25A22*	5.48091	4.97025
P10321	HLA class I histocompatibility antigen, Cw-7 alpha chain	*HLA-C*	5.46607	6.74616
P05556	Integrin beta-1	*ITGB1*	5.44772	4.647
Q15165	Serum paraoxonase/arylesterase 2	*PON2*	5.41825	6.28091
Q9HCU5	Prolactin regulatory element-binding protein	*PREB*	5.21519	4.37881
P01892;P10316;P01891;P30459	HLA class I histocompatibility antigen, A-2 alpha chain;HLA class I histocompatibility antigen, A-69 alpha chain;HLA class I histocompatibility antigen, A-68 alpha chain;HLA class I histocompatibility antigen, A-74 alpha chain	*HLA-A*	5.18994	8.71486
Q8NBM4	Ubiquitin-associated domain-containing protein 2	*UBAC2*	5.17362	1.90826
Q9P2E5	Chondroitin sulfate glucuronyltransferase	*CHPF2*	5.17219	3.31574
O43747	AP-1 complex subunit gamma-1	*AP1G1*	5.16727	2.21284
P61619	Protein transport protein Sec61 subunit alpha isoform 1	*SEC61A1*	4.95791	6.64721
Q96KA5	Cleft lip and palate transmembrane protein 1-like protein	*CLPTM1L*	4.94787	2.56906
Q9NYU1	UDP-glucose:glycoprotein glucosyltransferase 2	*UGGT2*	4.87748	5.86539
Q9Y5L0	Transportin-3	*TNPO3*	4.851	5.26718
P13667	Protein disulfide-isomerase A4	*PDIA4*	4.80127	4.401
P61221	ATP-binding cassette sub-family E member 1	*ABCE1*	4.78569	1.76521
Q6ZRP7	Sulfhydryl oxidase 2	*QSOX2*	4.61757	5.06459
Q96DZ1	Endoplasmic reticulum lectin 1	*ERLEC1*	4.53432	3.91795
Q92947	Glutaryl-CoA dehydrogenase, mitochondrial	*GCDH*	4.52998	4.57925
Q8TC07	TBC1 domain family member 15	*TBC1D15*	4.4356	5.12027
P57678	Gem-associated protein 4	*GEMIN4*	4.39941	3.75927
Q8N3Y3	Glycosyltransferase-like protein LARGE2;Xylosyltransferase LARGE2;Beta-1,3-glucuronyltransferase LARGE2	*GYLTL1B*	4.3815	4.24627
O15269	Serine palmitoyltransferase 1	*SPTLC1*	4.33366	5.23772
Q5TAQ9	DDB1- and CUL4-associated factor 8	*DCAF8*	4.31973	4.28822
Q02978	Mitochondrial 2-oxoglutarate/malate carrier protein	*SLC25A11*	4.31928	4.7761
Q9BW27	Nuclear pore complex protein Nup85	*NUP85*	4.21892	4.16292

Q9BRT8;Q8IUF1;Q5RIA9;Q5JTY5;Q4V339	COBW domain-containing protein 1;COBW domain-containing protein 2;COBW domain-containing protein 5;COBW domain-containing protein 3;COBW domain-containing protein 6	*CBWD1;CBWD2;CBWD5;CBWD3;CBWD6*	4.16984	4.07014
P11717	Cation-independent mannose-6-phosphate receptor	*IGF2R*	4.0737	5.33048
P10253	Lysosomal alpha-glucosidase;76 kDa lysosomal alpha-glucosidase;70 kDa lysosomal alpha-glucosidase	*GAA*	4.03236	2.09295
Q9H0X4	Protein ITFG3	*ITFG3*	4.03216	3.78316
Q8WWC4	Uncharacterized protein C2orf47, mitochondrial	*C2orf47*	3.98712	4.43023
Q9NX61	Transmembrane protein 161A	*TMEM161A*	3.98409	2.74751
P27824	Calnexin	*CANX*	3.97437	9.26043
Q7L8L6	FAST kinase domain-containing protein 5	*FASTKD5*	3.94827	4.61435
O60762	Dolichol-phosphate mannosyltransferase subunit 1	*DPM1*	3.92794	3.8626
Q9Y653	G-protein coupled receptor 56;GPR56 N-terminal fragment;GPR56 C-terminal fragment	*GPR56*	3.8648	3.97598
Q5H9R7	Serine/threonine-protein phosphatase 6 regulatory subunit 3	*PPP6R3*	3.84825	3.91826
Q92673	Sortilin-related receptor	*SORL1*	3.82489	8.96156
Q8TEM1	Nuclear pore membrane glycoprotein 210	*NUP210*	3.81807	3.93744
O94822	E3 ubiquitin-protein ligase listerin	*LTN1*	3.79809	5.43276
Q6P3X3	Tetratricopeptide repeat protein 27	*TTC27*	3.79526	5.3366
O60613	15 kDa selenoprotein	*42628*	3.78446	3.82451
Q96P70	Importin-9	*IPO9*	3.75761	4.2719
O43149	Zinc finger ZZ-type and EF-hand domain-containing protein 1	*ZZEF1*	3.7213	5.92507
Q9HC07	Transmembrane protein 165	*TMEM165*	3.70854	3.80655
Q6YHU6	Thyroid adenoma-associated protein	*THADA*	3.66756	4.39736
Q9BXS4	Transmembrane protein 59	*TMEM59*	3.66069	4.17617
Q9H497	Torsin-3A	*TOR3A*	3.65872	4.71453
P46977	Dolichyl-diphosphooligosaccharide--protein glycosyltransferase subunit STT3A	*STT3A*	3.62803	5.95155
Q9BT22	Chitobiosyldiphosphodolichol beta-mannosyltransferase	*ALG1*	3.61792	5.62443
O75976	Carboxypeptidase D	*CPD*	3.61184	3.95187
P51571	Translocon-associated protein subunit delta	*SSR4*	3.5527	4.62907
Q9NX57	Ras-related protein Rab-20	*RAB20*	3.54591	5.45739
Q9UJS0	Calcium-binding mitochondrial carrier protein Aralar2	*SLC25A13*	3.54523	2.78334
P19021	Peptidyl-glycine alpha-amidating monooxygenase;Peptidylglycine alpha-hydroxylating monooxygenase;Peptidyl-alpha-hydroxyglycine alpha-amidating lyase	*PAM*	3.42874	3.6497
Q6P988	Palmitoleoyl-protein carboxylesterase NOTUM	*NOTUM*	3.38472	5.12362
O43592	Exportin-T	*XPOT*	3.38404	5.9641
Q13042	Cell division cycle protein 16 homolog	*CDC16*	3.31605	3.89515
Q9NXH8	Torsin-4A	*TOR4A*	3.28504	3.38634
Q9BWS9	Chitinase domain-containing protein 1	*CHID1*	3.28324	3.78836
O75197	Low-density lipoprotein receptor-related protein 5	*LRP5*	3.26966	4.18258
O60568	Procollagen-lysine,2-oxoglutarate 5-dioxygenase 3	*PLOD3*	3.25464	3.12973
P55060	Exportin-2	*CSE1L*	3.19635	2.3847
P52789	Hexokinase-2	*HK2*	3.17721	3.26775
Q969V3	Nicalin	*NCLN*	3.1766	2.60123
Q9NZ08	Endoplasmic reticulum aminopeptidase 1	*ERAP1*	3.16621	3.93249
Q96T76	MMS19 nucleotide excision repair protein homolog	*MMS19*	3.14957	3.4413
Q9H2H9	Sodium-coupled neutral amino acid transporter 1	*SLC38A1*	3.14008	3.2158

Q9C0E2	Exportin-4	*XPO4*	3,08311	3,47998
Q8NBJ5	Procollagen galactosyltransferase 1	*COLGALT1*	3,07312	3,30757
Q9ULC5	Long-chain-fatty-acid--CoA ligase 5	*ACSL5*	3,06398	4,136
Q15645	Pachytene checkpoint protein 2 homolog	*TRIP13*	3,0587	3,70127
Q9NYP7	Elongation of very long chain fatty acids protein 5	*ELOVL5*	3,04476	4,74811
Q13438	Protein OS-9	*OS9*	3,0428	3,88637
Q8IZ52	Chondroitin sulfate synthase 2	*CHPF*	3,04053	3,37988
Q12769	Nuclear pore complex protein Nup160	*NUP160*	2,99621	4,33823
Q9HD45	Transmembrane 9 superfamily member 3	*TM9SF3*	2,95684	4,88098
Q5T160	Probable arginine--tRNA ligase, mitochondrial	*RARS2*	2,92549	3,32649
Q15751	Probable E3 ubiquitin-protein ligase HERC1	*HERC1*	2,92195	3,60025
Q92973	Transportin-1	*TNPO1*	2,92082	3,08836
P30508;Q29960;Q07000;P30505;Q9TNN7;P30504;P30501	HLA class I histocompatibility antigen, Cw-12 alpha chain;HLA class I histocompatibility antigen, Cw-16 alpha chain;HLA class I histocompatibility antigen, Cw-15 alpha chain;HLA class I histocompatibility antigen, Cw-8 alpha chain;HLA class I histocompatibility antigen, Cw-5 alpha chain;HLA class I histocompatibility antigen, Cw-4 alpha chain;HLA class I histocompatibility antigen, Cw-2 alpha chain	*HLA-C*	2,92033	2,50506
P33527	Multidrug resistance-associated protein 1	*ABCC1*	2,87818	2,49586
P05534;P30447	HLA class I histocompatibility antigen, A-24 alpha chain;HLA class I histocompatibility antigen, A-23 alpha chain	*HLA-A*	2,84853	3,9893
Q9Y4R8	Telomere length regulation protein TEL2 homolog	*TELO2*	2,84767	3,78873
Q92621	Nuclear pore complex protein Nup205	*NUP205*	2,77102	2,00909
Q6UX53	Methyltransferase-like protein 7B	*METTL7B*	2,76825	2,28236
Q96JJ7	Protein disulfide-isomerase TMX3	*TMX3*	2,75799	5,0311
O95302	Peptidyl-prolyl cis-trans isomerase FKBP9	*FKBP9*	2,75191	5,7395
P55036	26S proteasome non-ATPase regulatory subunit 4	*PSMD4*	2,74588	1,63303
Q8N4Q1	Mitochondrial intermembrane space import and assembly protein 40	*CHCHD4*	2,73883	4,89078
Q9H490	Phosphatidylinositol glycan anchor biosynthesis class U protein	*PIGU*	2,73568	2,62799
Q13617	Cullin-2	*CUL2*	2,72629	3,55656
Q08AM6	Protein VAC14 homolog	*VAC14*	2,69271	5,37085
Q8IZV5	Retinol dehydrogenase 10	*RDH10*	2,62522	1,57007
Q9UM00	Transmembrane and coiled-coil domain-containing protein 1	*TMCO1*	2,61709	1,97214
Q13315	Serine-protein kinase ATM	*ATM*	2,50435	3,18972
Q8IXM3	39S ribosomal protein L41, mitochondrial	*MRPL41*	2,4758	1,65053
Q9HCN8	Stromal cell-derived factor 2-like protein 1	*SDF2L1*	2,41969	4,67022
Q14517	Protocadherin Fat 1;Protocadherin Fat 1, nuclear form	*FAT1*	2,39691	4,14644
Q13619	Cullin-4A	*CUL4A*	2,37824	3,72551
O00567	Nucleolar protein 56	*NOP56*	2,3765	2,28444
Q9Y6D6	Brefeldin A-inhibited guanine nucleotide-exchange protein 1	*ARFGEF1*	2,3472	2,70252
Q12797	Aspartyl/asparaginyl beta-hydroxylase	*ASPH*	2,31702	4,41349
O00165	HCLS1-associated protein X-1	*HAX1*	2,30784	3,67671
P12235	ADP/ATP translocase 1	*SLC25A4*	2,3025	3,10851
Q9UBB4	Ataxin-10	*ATXN10*	2,28645	2,79558
O15397	Importin-8	*IPO8*	2,27648	2,67801
P53007	Tricarboxylate transport protein, mitochondrial	*SLC25A1*	2,27146	3,65107
O14925;Q5SRD1	Mitochondrial import inner membrane translocase subunit Tim23;Putative mitochondrial import inner membrane translocase subunit Tim23B	*TIMM23;TIMM23B*	2,26358	2,51422

Q96A33	Coiled-coil domain-containing protein 47	*CCDC47*	2,2498	3,14363
P49959	Double-strand break repair protein MRE11A	*MRE11A*	2,24556	1,40589
Q8WUJ3	Cell migration-inducing and hyaluronan-binding protein	*CEMIP*	2,19967	3,06834
Q13098	COP9 signalosome complex subunit 1	*GPS1*	2,16271	2,46644
Q9Y5M8	Signal recognition particle receptor subunit beta	*SRPRB*	2,09703	3,14697
Q9P2R7	Succinyl-CoA ligase [ADP-forming] subunit beta, mitochondrial	*SUCLA2*	2,04178	3,33813
Q8WUY1	Protein THEM6	*THEM6*	2,02123	1,49977
Q96AY3	Peptidyl-prolyl cis-trans isomerase FKBP10	*FKBP10*	1,97278	1,3684
Q14393	Growth arrest-specific protein 6	*GAS6*	1,86818	3,72892
Q13217	DnaJ homolog subfamily C member 3	*DNAJC3*	1,71242	3,55121
O60488	Long-chain-fatty-acid--CoA ligase 4	*ACSL4*	1,52078	2,26157
Q96HE7	ERO1-like protein alpha	*ERO1L*	1,39717	1,49019
Q8WVX9	Fatty acyl-CoA reductase 1	*FAR1*	1,38572	3,10168

Supplementary Table 6. Gene ontology (GO) terms obtained from proteomic study of GCNT3 interactors.

Items	Items_Details	Support	List size	Reference Support	Reference size	Hyp	Hyp_c	Genes
GO:0044267	cellular protein metabolic process (BP)	105	718	284	34208	8,92E-102	1,34E-98	TUBA1C,RPL10,TUBA1A,UGGT2,RPS16,RPN2,RPL12,RPS14,RPL3,RPL36,RPS26,RPL5,RPL11,RPS25,EEF2,TUBA4A,CCT4,RPS13,RPL36A,CCT2,RPL7,RPL32,RPL7A,EEF1G,RPS7,TUBB2B,RPL37A,RPS15,DPM1,RPL23,RPL15,RPL28,RPL14,RPL13,PDIA3,RPS19,EIF3C,PABPC1,RPS9,CCT6A,UBA52,RPSA,TUBB2A,GAS6,TUBB2C,EEF1A1,RPS23,RPS4X,HSPD1,CCT5,RPL18A,EIF5A2,RPS18,RPS24,TCP1,EIF4A1,EIF5A,RPS12,RPS2,RPL38,RPL22,RPS11,STT3A,RPL24,PREB,RPL35,CCT8,RPL27,SEC13,GANAB,RPL35A,PIGU,CCT3,DDOST,RPL18,RPS5,RPL9,EEF1B2,CALR,RPL13A,UGGT1,RPL30,ALG1,CANX,RPL21,TUBB3,RPLP0,RPN1,RPS20,RPL6,RPLP2,RPS8,RPL8,RPL17,CCT7,RPS27,RPS3,RPS3A,RPL27A,RPL29,RPL4,RPL23A,RPS6,RPL10A,RPS15A
GO:0016071	mRNA metabolic process (BP)	95	718	223	34208	3,99E-99	2,99E-96	RPL10,TNPO1,RPS16,PSMA2,HNRNPD,RPL12,RPS14,RPL3,RPL36,TNKS1BP1,PSMC2,RPS26,RPL5,RPL11,RPS25,PSMC5,PCBP1,RPS13,CNOT1,RPL36A,RPL7,RPL32,UBB,RPL7A,PSMA7,PSMD11,PABPC3,RPS7,RPL37A,RPS15,RPL23,PCBP2,RPL15,RPL28,HSPB1,RPL14,RPL13,RPS19,PABPC1,PSMD6,RPS9,UBA52,RPSA,YWHAZ,RPS23,PSMD4,RPS4X,RPL18A,HSPA8,PSMD8,RPS18,RPS24,PPP2R1A,PSMD12,EIF4A1,UBC,RPS12,RPS2,RPL38,RPL22,PSMC3,RPS11,RPL24,RPL35,RPL27,RPL35A,RPL18,RPS5,RPL9,RPL13A,XPO1,RPL30,RPL21,RPLP0,RPS20,RPL6,RPLP2,RPS8,UPF1,PSMD2,RPL8,RPL17,RPS27,RPS3,PSMA4,RPS3A,HSPA1A,RPL27A,RPL29,RPL4,RPL23A,RPS6,RPL10A,RPS15A,PSMD3
GO:0006414	translational elongation (BP)	70	718	93	34208	2,78E-98	1,39E-95	RPL10,RPS16,RPL12,RPS14,RPL3,RPL36,RPS26,RPL5,RPL11,RPS25,EEF2,RPS13,RPL36A,RPL7,RPL32,RPL7A,EEF1G,RPS7,RPL37A,RPS15,RPL23,RPL15,RPL28,RPL14,RPL13,RPS19,TUFM,RPS9,UBA52,RPSA,EEF1A1,RPS23,RPS4X,RPL18A,RPS18,RPS24,EIF5A,RPS12,RPS2,RPL38,RPL22,RPS11,RPL24,RPL35,RPL27,RPL35A,RPL18,RPS5,RPL9,EEF1B2,RPL13A,RPL30,RPL21,RPLP0,RPS20,RPL6,RPLP2,RPS8,RPL8,RPL17,RPS27,RPS3,RPS3A,RPL27A,RPL29,RPL4,RPL23A,RPS6,RPL10A,RPS15A
GO:0010467	gene expression (BP)	116	718	408	34208	2,18E-97	8,18E-95	RPL10,RPS16,HNRNPD,RPL12,RPS14,RPL3,RPL36,HNRNPC,RPS26,RPL5,RPL11,RPS25,TRIM28,EEF2,HNRNPK,HNRNPF,PCBP1,GARS,DARS,RPS13,SRSF3,RPL36A,SNRPE,KARS,RPL7,RPL32,RPL7A,EEF1G,RPS7,RPL37A,RPS15,RPL23,PCBP2,RPL15,RPL28,TARS,RPL14,RPL13,PRPF8,RBMX,SNRNP200,RPS19,EIF3C,PABPC1,RPS9,UBA52,SRSF7,SF3B3,SMC1A,AIMP2,RPSA,SRSF1,HNRNPH2,EEF1A1,RPS23,PTBP1,RPS4X,RPL18A,RPS18,SF3B1,RPS24,EIF4A1,RPS12,RPS2,RPL38,SNRPF,RPL22,RPS11,TCEB1,RPL24,RPL35,TCEB2,RPL27,EPRS,DHX9,RPL35A,HNRNPU,SNRPD1,FUS,RARS2,RPL18,RPS5,RPL9,EEF1B2,THOC4,RPL13A,LARS,RPL30,RARS,HNRNPM,HNRNPA2B1,EFTUD2,RPL21,RPLP0,RPS20,RPL6,YBX1,MARS,RPLP2,RPS8,HNRNPUL1,RPL8,RPL17,HNRNPH1,RPS27,RPS3,RPS3A,RPL27A,RPL29,HNRNPA1,IARS,RPL4,RPL23A,RPS6,RPL10A,RPS15A
GO:0016070	RNA metabolic process (BP)	97	718	257	34208	5,78E-95	1,73E-92	RPL10,TNPO1,RPS16,PSMA2,HNRNPD,RPL12,RPS14,RPL3,RPL36,TNKS1BP1,PSMC2,RPS26,RPL5,RPL11,RPS25,PSMC5,RPS13,CNOT1,RPL36A,SNRPE,RPL7,RPL32,UBB,RPL7A,PSMA7,CNP,PSMD11,RPS7,RPL37A,RPS15,RPL23,RPL15,RPL28,HSPB1,RPL14,RPL13,RPS19,PABPC1,PSMD6,RPS9,UBA52,RPSA,YWHAZ,RPS23,GEMIN4,PSMD4,RPS4X,RPL18A,HSPA8,PSMD8,RPS18,RPS24,PPP2R1A,PSMD12,EIF4A1,UBC,RPS12,RPS2,RPL38,SNRPF,RPL22,PSMC3,RPS11,RPL24,RPL35,RPL27,RPL35A,SNRPD1,RPL18,RPS5,RPL9,RPL13A,XPO1,RPL30,RPL21,RPLP0,RPS20,RPL6,RPLP2,RPS8,UPF1,PSMD2,RPL8,RPL17,RPS27,RPS3,PSMA4,RPS3A,HSPA1A,RPL27A,RPL29,RPL4,RPL23A,RPS6,RPL10A,RPS15A,PSMD3
GO:0019058	viral infectious cycle (BP)	67	718	91	34208	6,31E-93	1,58E-90	RPL10,KPNB1,RPS16,RPL12,RPS14,RPL3,RPL36,RPS26,RPL5,RPL11,RPS25,RPS13,RPL36A,RPL7,RPL32,RPL7A,RPS7,RPL37A,RPS15,RPL23,RPL15,RPL28,RPL14,RPL13,RPS19,RPS9,UBA52,RPSA,RPS23,RPS4X,RPL18A,RPS18,RPS24,RPS12,RPS2,RPL38,RPL22,RPS11,RPL24,RPL35,RPL27,RPL35A,SLC25A6,RPL18,RPS5,RPL9,RPL13A,XPO1,RPL30,RPL21,RPLP0,RPS20,RPL6,RPLP2,RPS8,RPL8,RPL17,RPS27,RPS3,RPS3A,RPL27A,RPL29,RPL4,RPL23A,RPS6,RPL10A,RPS15A
GO:0019083	viral transcription (BP)	64	718	82	34208	9,88E-92	2,12E-89	RPL10,RPS16,RPL12,RPS14,RPL3,RPL36,RPS26,RPL5,RPL11,RPS25,RPS13,RPL36A,RPL7,RPL32,RPL7A,RPS7,RPL37A,RPS15,RPL23,RPL15,RPL28,RPL14,RPL13,RPS19,RPS9,UBA52,RPSA,RPS23,RPS4X,RPL18A,RPS18,RPS24,RPS12,RPS2,RPL38,RPL22,RPS11,RPL24,RPL35,RPL27,RPL35A,RPL18,RPS5,RPL9,RPL13A,RPL30,RPL21,RPLP0,RPS20,RPL6,RPLP2,RPS8,RPL8,RPL17,RPS27,RPS3,RPS3A,RPL27A,RPL29,RPL4,RPL23A,RPS6,RPL10A,RPS15A
GO:0016032	viral reproduction (BP)	102	718	329	34208	9,76E-90	1,83E-87	RPL10,KPNB1,RPS16,PSMA2,XRCC6,RPL12,RPS14,RPL3,RPL36,PSMC2,RPS26,RPL5,RPL11,RPS25,SLC25A4,PPIA,PSMC5,RPS13,RPL36A,RPL7,RPL32,UBB,RPL7A,PSMA7,PSMD11,RPS7,RPL37A,RPS15,RPL23,NUP210,RPL15,RPL28,RPL14,RPL13,RPS19,PSMD6,RPS9,UBA52,RPSA,RPS23,PSMD4,RPS4X,RPL18A,PSMD8,RPS18,RPS24,AP1B1,AP1G1,PSMD12,UBC,RPS12,RPS2,RPL38,RPL22,PSMC3,RPS11,TCEB1,RPL24,RPL35,TCEB2,RPL27,RPL35A,SKP1,NUP160,SLC25A6,RPL18,RPS5,RPL9,NUP205,RPL13A,XPO1,NUP93,RPL30,AP2B1,RPL21,RPLP0,RPS20,RPL6,XRCC5,RPLP2,BANF1,RPS8,CUL5,NUP85,PSMD2,RPL8,RPL17,RPS27,RPS3,AP2A1,PSMA4,RPS3A,RPL27A,RPL29,SLC25A5,RPL4,LCK,RPL23A,RPS6,RPL10A,RPS15A,PSMD3
GO:0006415	translational termination (BP)	64	718	87	34208	9,86E-89	1,64E-86	RPL10,RPS16,RPL12,RPS14,RPL3,RPL36,RPS26,RPL5,RPL11,RPS25,RPS13,RPL36A,RPL7,RPL32,RPL7A,RPS7,RPL37A,RPS15,RPL23,RPL15,RPL28,RPL14,RPL13,RPS19,RPS9,UBA52,RPSA,RPS23,RPS4X,RPL18A,RPS18,RPS24,RPS12,RPS2,RPL38,RPL22,RPS11,RPL24,RPL35,RPL27,RPL35A,RPL18,RPS5,RPL9,RPL13A,RPL30,RPL21,RPLP0,RPS20,RPL6,RPLP2,RPS8,RPL8,RPL17,RPS27,RPS3,RPS3A,RPL27A,RPL29,RPL4,RPL23A,RPS6,RPL10A,RPS15A
GO:0031018	endocrine pancreas development (BP)	64	718	121	34208	1,26E-74	1,90E-72	RPL10,RPS16,RPL12,RPS14,RPL3,RPL36,RPS26,RPL5,RPL11,RPS25,RPS13,RPL36A,RPL7,RPL32,RPL7A,RPS7,RPL37A,RPS15,RPL23,RPL15,RPL28,RPL14,RPL13,RPS19,RPS9,UBA52,RPSA,RPS23,RPS4X,RPL18A,RPS18,RPS24,RPS12,RPS2,RPL38,RPL22,RPS11,RPL24,RPL35,RPL27,RPL35A,RPL18,RPS5,RPL9,RPL13A,RPL30,RPL21,RPLP0,RPS20,RPL6,RPLP2,RPS8,RPL8,RPL17,RPS27,RPS3,RPS3A,RPL27A,RPL29,RPL4,RPL23A,RPS6,RPL10A,RPS15A

GO:0006412	translation (BP)	80	718	241	34208	5,81E-73	7,93E-71	RPL10,RPS16,RPL12,RPS14,RPL3,RPL36,RPS26,RPL5,RPL11,RPS25,EEF2,DARS,RPS13,RPL36A,RPL7,RPL32,RPL7A,EEF1G,RPS7,RPL37A,RPS15,RPL23,RPL15,RPL28,TARS,RPL14,RPL13,RPS19,EIF3C,PABPC1,RPS9,UBA52,RPSA,EEF1A1,RPS23,RPL22L1,RPS4X,RPL18A,RPS18,RPS24,PABPC4,EIF4A1,RPS12,RPS2,RPL38,RPL22,RPS11,SLC25A10,RPL24,RPL35,RPL27,RPL35A,RPL18,RPS5,RPL9,EEF1B2,RPL13A,RPL30,IGF2BP3,RPL21,RPLP0,MRPL41,GCN1L1,RPS20,RPL6,RPLP2,RPS8,RPL8,RPL17,RPS27,RPS3,RPL36AL,RPS3A,RPL27A,RPL29,RPL4,RPL23A,RPS6,RPL10A,RPS15A
GO:0006334	nucleosome assembly (BP)	36	718	102	34208	2,12E-34	2,65E-32	HIST1H2BM,HIST1H2BC,H2AFZ,HIST1H1E,H2AFJ,NPM1,HIST1H2BD,HIST1H2AD,HIST3H2A,HIST2H2AC,HIST1H2AG,HIST1H2AH,HIST1H2BH,H2AFV,HIST3H3,H3F3A,HIST2H2AA3,HIST1H1C,HIST1H2BN,HIST1H2BK,HIST3H2BB,RUVBL1,HIST1H2AJ,NAP1L1,HIST1H2BO,HIST1H1D,HIST1H2BA,HIST2H2BF,H2AFX,HIST1H2AA,HIST1H4A,HIST1H2BJ,HIST1H2BB,HIST1H2AB,HIST2H2BE,HIST1H2AC
GO:0006457	protein folding (BP)	40	718	182	34208	4,79E-29	5,53E-27	TUBA1C,TUBA1A,UGGT2,PDIA6,DNAJA1,PPIA,TUBA4A,CCT4,CCT2,RUVBL2,TUBB2B,HSP90AA1,PDIA3,CCT6A,DNAJA3,TUBB2A,TUBB2C,CCT5,HSPA8,BAG2,TCP1,PPIB,DNAJA2,FKBP9,HSPA9,CCT8,GANAB,FKBP10,CCT3,RP2,CALR,UGGT1,CANX,DNAJB11,TUBB3,DNAJC3,HSP90AB1,ERO1L,CCT7,HSP90B1
GO:0008380	RNA splicing (BP)	45	718	267	34208	2,62E-27	2,80E-25	HNRNPD,LGALS3,HNRNPA1L2,HNRNPC,HNRNPK,HNRNPF,PCBP1,SRSF3,SNRPE,PCBP2,PRPF8,RBMX,SNRNP200,PABPC1,SRSF7,SF3B3,SMC1A,SRSF1,HNRNPH2,GEMIN4,DHX15,PTBP1,NONO,SF3B1,DDX5,PPP2R1A,SNRPF,DHX9,STRAP,HNRNPU,SNRPD1,FUS,THOC4,HNRNPM,HNRNPA2B1,EFTUD2,DDX39A,YBX1,RBMXL1,PRPF39,SFPQ,HNRNPUL1,HNRNPH1,SYNCRIP,HNRNPA1
GO:0000398	nuclear mRNA splicing, via spliceosome (BP)	35	718	167	34208	7,95E-25	7,95E-23	HNRNPD,HNRNPC,HNRNPK,HNRNPF,PCBP1,SRSF3,SNRPE,PCBP2,PRPF8,RBMX,SNRNP200,PABPC1,SRSF7,SF3B3,SMC1A,SRSF1,HNRNPH2,PTBP1,SF3B1,DDX5,SNRPF,DHX9,HNRNPU,SNRPD1,FUS,THOC4,HNRNPM,HNRNPA2B1,EFTUD2,DDX39A,YBX1,HNRNPUL1,HNRNPH1,SYNCRIP,HNRNPA1
GO:0044419	interspecies interaction between organisms (BP)	46	718	328	34208	2,23E-24	2,09E-22	TNPO1,KPNB1,IPO7,C1QBP,PSMC2,KRT18,SLC25A4,HNRNPK,PPIA,NPM1,KARS,YWHAE,VAC14,PSMA7,HLA-A,DDB1,PDCD6IP,IPO5,CUL2,DYNLL1,HSPD1,HSPA8,GNB2L1,SCRIB,PSMC3,TCEB1,COPB1,SLC25A6,VIM,HLA-C,THOC4,XPO1,RPLP0,BANF1,CUL5,TFRC,SYNCRIP,VDAC1,PSMA4,HNRNPA1,SLC25A5,LCK,DDX3X,CUL4A,ITGB1,ABCE1
GO:0000278	mitotic cell cycle (BP)	38	718	304	34208	1,34E-18	1,18E-16	TUBA1A,PSMA2,DYNC1H1,PSMC2,TUBB,TUBA4A,PSMC5,POLD4,YWHAE,UBB,PSMA7,CDK1,PSMD11,HSP90AA1,PRKACA,PSMD6,UBA52,SMC1A,TUBB2C,DYNLL1,PSMD4,PSMD8,PPP2R1A,PSMD12,UBC,CDC16,RFC5,PSMC3,SEC13,SKP1,NUP160,XPO1,NUP85,PSMD2,MCM3,RPS27,PSMA4,PSMD3
GO:0002474	antigen processing and presentation of peptide antigen via MHC class I (BP)	23	718	94	34208	2,20E-18	1,83E-16	PSMA2,PSMC2,PSMC5,UBB,PSMA7,PSMD11,PDIA3,HLA-A,PSMD6,UBA52,PSMD4,PSMD8,ERAP1,PSMD12,UBC,PSMC3,SEC13,HLA-C,CALR,CANX,PSMD2,PSMA4,PSMD3
GO:0051084	'de novo' posttranslational protein folding (BP)	16	718	36	34208	5,95E-18	4,70E-16	TUBA1C,TUBA1A,TUBA4A,CCT4,CCT2,TUBB2B,CCT6A,TUBB2A,TUBB2C,CCT5,TCP1,CCT8,CCT3,UGGT1,TUBB3,CCT7
GO:0051437	positive regulation of ubiquitin-protein ligase activity involved in mitotic cell cycle (BP)	19	718	71	34208	3,24E-16	2,43E-14	PSMA2,PSMC2,PSMC5,UBB,PSMA7,CDK1,PSMD11,PSMD6,UBA52,PSMD4,PSMD8,PSMD12,UBC,CDC16,PSMC3,SKP1,PSMD2,PSMA4,PSMD3
GO:0006200	ATP catabolic process (BP)	20	718	82	34208	3,90E-16	2,79E-14	ACTC1,PSMC2,ATP5B,MYH14,PSMC5,ATP2A2,RUVBL2,HSP90AA1,PSMD6,HSPA5,ATP6V0D1,MYH9,HSPD1,HSPA8,ATP5C1,MSH2,ATP1A1,ATP5A1,MACF1,ABCC1
GO:0000075	cell cycle checkpoint (BP)	24	718	131	34208	4,95E-16	3,38E-14	PSMA2,PSMC2,PSMC5,UBB,PSMA7,CDK1,PSMD11,DDB1,PSMD6,UBA52,SMC1A,PSMD4,PSMD8,PSMD12,UBC,CDC16,RFC5,PSMC3,PSMD2,MCM3,MRE11A,ATM,PSMA4,PSMD3
GO:0000082	G1/S transition of mitotic cell cycle (BP)	25	718	145	34208	5,42E-16	3,54E-14	PSMA2,PSMC2,PSMC5,UBB,PSMA7,CDK1,PSMD11,PPP6C,PSMD6,UBA52,CUL2,PSMD4,PSMD8,PSMD12,UBC,PSMC3,SKP1,CAMK2D,CUL5,PSMD2,MCM3,PSMA4,CUL4A,PSMD3,ITGB1
GO:0006928	cellular component movement (BP)	21	718	95	34208	6,18E-16	3,87E-14	ACTR2,TUBB,DSTN,ARPC2,ARPC1B,HSPB1,CAPZA1,ACTR3,TUBB2C,CALD1,MYH9,TPM4,CAPZB,TPM1,ACTG1,ARPC3,VIM,CAPZA2,ARPC5,TPM3,MACF1
GO:0051439	regulation of ubiquitin-protein ligase activity involved in mitotic cell cycle (BP)	19	718	75	34208	9,92E-16	5,95E-14	PSMA2,PSMC2,PSMC5,UBB,PSMA7,CDK1,PSMD11,PSMD6,UBA52,PSMD4,PSMD8,PSMD12,UBC,CDC16,PSMC3,SKP1,PSMD2,PSMA4,PSMD3

GO:0031145	anaphase-promoting complex-dependent proteasomal ubiquitin-dependent protein catabolic process (BP)	19	718	80	34208	3,64E-15	2,10E-13	PSMA2,PSMC2,PSMC5,UBB,PSMA7,CDK1,PSMD11,PSMD6,UBA52,PSMD4,PSMD8,PSMD12,UBC,CDC16,PSMC3,SKP1,PSMD2,PSMA4,PSMD3
GO:0051436	negative regulation of ubiquitin-protein ligase activity involved in mitotic cell cycle (BP)	17	718	65	34208	1,81E-14	1,00E-12	PSMA2,PSMC2,PSMC5,UBB,PSMA7,PSMD11,PSMD6,UBA52,PSMD4,PSMD8,PSMD12,UBC,CDC16,PSMC3,PSMD2,PSMA4,PSMD3
GO:0006977	DNA damage response, signal transduction by p53 class mediator resulting in cell cycle arrest (BP)	17	718	66	34208	2,39E-14	1,28E-12	PSMA2,PSMC2,PSMC5,UBB,PSMA7,PSMD11,PSMD6,UBA52,PSMD4,PSMD8,PSMD12,UBC,PSMC3,PSMD2,ATM,PSMA4,PSMD3
GO:0006915	apoptotic process (BP)	46	718	594	34208	4,09E-14	2,12E-12	KPNB1,PSMA2,ACTC1,PLEC,SRA1,PSMC2,TJP2,PSMC5,YWHAE,UBB,PSMA7,CDK1,PSMD11,DSP,LMNB1,SPTAN1,PDCD6IP,PSMD6,UBA52,TJP1,CASP14,RHOT1,AIMP2,CSE1L,DYNLL2,DNAJA3,DYNLL1,PSMD4,PSMD8,PSMD12,LGALS1,UBC,CTNNB1,PSMC3,CLPTM1L,LMNA,SLC25A6,VIM,CYFIP2,MRPL41,PSMD2,SQSTM1,VDAC1,PSMA4,LCK,PSMD3
GO:0000084	S phase of mitotic cell cycle (BP)	20	718	112	34208	2,28E-13	1,14E-11	PSMA2,PSMC2,PSMC5,POLD4,UBB,PSMA7,PSMD11,PSMD6,UBA52,PSMD4,PSMD8,PSMD12,UBC,RFC5,PSMC3,SKP1,PSMD2,MCM3,PSMA4,PSMD3
GO:0000216	M/G1 transition of mitotic cell cycle (BP)	17	718	78	34208	4,71E-13	2,28E-11	PSMA2,PSMC2,PSMC5,UBB,PSMA7,PSMD11,PSMD6,UBA52,PSMD4,PSMD8,PSMD12,UBC,PSMC3,PSMD2,MCM3,PSMA4,PSMD3
GO:0006886	intracellular protein transport (BP)	25	718	196	34208	6,76E-13	3,17E-11	TMED10,TNPO1,IPO7,ARCN1,IPO4,NPM1,IPO8,COPA,TIMM23,IPO5,CSE1L,MYO6,AP1B1,AP1G1,IPO9,SEC13,CLTC,COPB1,XPO5,SSR4,COPB2,XPO1,AP2B1,XPOT,AP2A1
GO:0042981	regulation of apoptotic process (BP)	25	718	205	34208	1,87E-12	8,51E-11	PSMA2,PSMC2,DAPK3,PSMC5,PHB,UBB,PSMA7,PSMD11,PSMD6,UBA52,PSMD4,PSMD8,PPP2R1A,PSMD12,LGALS1,UBC,PSMC3,LMNA,CALR,PRDX2,ACTN1,PSMD2,PSMA4,ACTN4,PSMD3
GO:0006521	regulation of cellular amino acid metabolic process (BP)	13	718	51	34208	3,15E-11	1,39E-09	PSMA2,PSMC2,PSMC5,PSMA7,PSMD11,PSMD6,PSMD4,PSMD8,PSMD12,PSMC3,PSMD2,PSMA4,PSMD3
GO:0034641	cellular nitrogen compound metabolic process (BP)	23	718	200	34208	4,82E-11	2,07E-09	PSMA2,ALDH18A1,PSMC2,PSMC5,PSMA7,PSMD11,PSMD6,SLC1A5,PSMD4,SLC38A2,OGDH,PSMD8,PSMD12,SLC3A2,PSMC3,DLST,GCDH,HSD17B10,PSMD2,PSMA4,GSTZ1,SLC38A1,PSMD3
GO:0000209	protein polyubiquitination (BP)	17	718	107	34208	9,90E-11	4,12E-09	PSMA2,PSMC2,PSMC5,UBB,PSMA7,PSMD11,PSMD6,UBA52,PSMD4,PSMD8,PSMD12,UBC,UBR5,PSMC3,PSMD2,PSMA4,PSMD3
GO:0043687	post-translational protein modification (BP)	18	718	123	34208	1,14E-10	4,62E-09	UGGT2,RPN2,DPM1,PDIA3,GAS6,EIF5A2,EIF5A,STT3A,PREB,SEC13,GANAB,PIGU,DDOST,CALR,UGGT1,ALG1,CANX,RPN1
GO:0005975	carbohydrate metabolic process (BP)	27	718	290	34208	1,38E-10	5,45E-09	SLC25A1,PRPS1,PPP1CA,NUP210,ENO1,PRKACA,SLC2A1,CHID1,SLC3A2,ALDH1B1,PRKACB,SLC25A11,SLC25A10,GCNT3,GANAB,NUP160,PFKP,NUP205,GAA,NUP93,HK2,HKDC1,NUP85,PFKL,PPP1CB,SLC25A13,GAPDH

GO:0018279	protein N-linked glycosylation via asparagine (BP)	15	718	87	34208	3,80E-10	1,46E-08	UGGT2,RPN2,STT3B,DPM1,PDIA3,STT3A,PREB,SEC13,GANAB,DDOST,CALR,UGGT1,ALG1,CANX,RPN1
GO:0051028	mRNA transport (BP)	12	718	51	34208	5,01E-10	1,88E-08	HNRNPA1L2,NUP210,MYO1C,EIF5A2,EIF5A,SEC13,NUP205,XPO1,NUP93,LRPPRC,NUP85,HNRNPA1
GO:0006418	tRNA aminoacylation for protein translation (BP)	11	718	43	34208	1,01E-09	3,69E-08	GARS,DARS,KARS,TARS,AIMP2,EPRS,RARS2,LARS,RARS,MARS,IARS
GO:0035338	long-chain fatty-acyl-CoA biosynthetic process (BP)	8	718	18	34208	1,32E-09	4,70E-08	SLC25A1,FASN,HSD17B12,ACSL4,TECR,ACSL3,ELOVL5,ACSL5
GO:0006281	DNA repair (BP)	25	718	285	34208	2,28E-09	7,96E-08	PARP1,XRCC6,TRIM28,POLD4,FANCI,NPM1,UBB,RUVBL2,CDK1,DDB1,UBA52,SMC1A,PSMD4,NONO,UBC,RFC5,RUVBL1,MSH2,H2AFX,XRCC5,SFPQ,UPF1,RBM14,ATM,CUL4A
GO:0030833	regulation of actin filament polymerization (BP)	7	718	13	34208	2,68E-09	9,14E-08	ARPC2,ARPC1B,ACTR3,ARPC1A,ARPC3,ARPC5L,ARPC5
GO:0006112	energy reserve metabolic process (BP)	15	718	102	34208	3,77E-09	1,26E-07	IQGAP1,SLC25A4,PRKACA,FASN,SLC2A1,PRKACB,ITPR2,GNB1,PRKAR2A,SLC25A6,GNAS,GNB4,ITPR3,SLC25A5,GNB2
GO:0007596	blood coagulation (BP)	32	718	457	34208	3,87E-09	1,26E-07	C1QBP,PPIA,TUBA4A,ATP2A2,CFL1,PRKACA,CAPZA1,YWHAZ,HSPA5,GAS6,H3F3A,PABPC4,SLC3A2,PRKACB,ITPR2,CAPZB,SLC16A1,GNB1,CALU,ACTG1,FLNA,PRKAR2A,CAPZA2,GNAS,ITPR3,ACTN1,GNAI2,LCK,ACTN4,BSG,GNAI1,ITGB1
GO:0070934	CRD-mediated mRNA stabilization (BP)	5	718	5	34208	4,02E-09	1,28E-07	IGF2BP1,DHX9,HNRNPU,YBX1,SYNCRIP
GO:0015031	protein transport (BP)	30	718	413	34208	5,13E-09	1,60E-07	SEC16A,XPO4,IPO4,RAB20,NUP210,ARF4,VPS4A,PDCD6IP,MYO1C,MYH9,EIF5A2,RAB8A,EIF5A,TIMM50,TNPO3,SEC61B,PREB,IPO9,CHCHD4,NUP160,NUP205,NUP93,MYO5B,RAB6B,RAB8B,RAB6A,NUP85,SEC61A1,HSP90B1,ACTN4
GO:0006986	response to unfolded protein (BP)	11	718	51	34208	7,17E-09	2,19E-07	DNAJA1,HSPB1,HSP90AA1,HSPA6,HSPD1,HSPA8,HSPH1,DNAJC3,HSP90AB1,HSPA1A,SERPINH1
GO:0006810	transport (BP)	37	718	604	34208	8,76E-09	2,63E-07	TMED10,SLC25A1,IPO7,ARCN1,HNRNPA1L2,CYB5B,DYNC1H1,SLC25A3,ATP5B,SLC25A4,ATP2A2,UQCRC2,G3BP1,CPT1A,DYNLL2,OSBPL8,DYNLL1,SORL1,ITPR2,SLC25A11,SLC25A10,NDUFA4,ATP6V1A,SLC25A22,THOC4,NUP93,XPOT,HERC1,LRPPRC,SDHA,ERO1L,ABCD3,ETFA,SLC25A13,HNRNPA1,SLC25A5,ABCC1
GO:0016044	cellular membrane organization (BP)	14	718	95	34208	1,21E-08	3,50E-07	ARCN1,UBB,VPS4A,COPA,UBA52,HSPA8,AP1B1,AP1G1,UBC,PREB,SEC13,CLTC,COPB1,COPB2
GO:0006397	mRNA processing (BP)	20	718	202	34208	1,19E-08	3,50E-07	LGALS3,HNRNPA1L2,SNRPE,PRPF8,KHDRBS1,SRSF7,SF3B3,SRSF1,GEMIN4,DHX15,PTBP1,NONO,SNRPF,STRAP,HNRNPA2B1,EFTUD2,RBMXL1,PRPF39,SFPQ,HNRNPA1
GO:0006936	muscle contraction (BP)	14	718	96	34208	1,39E-08	3,94E-07	UTRN,FLII,MYL12B,CALD1,TPM4,SNTB1,ACTG2,ASPH,MYL6B,TPM1,MYL6,ACTA2,TPM3,ACTA1
GO:0006096	glycolysis (BP)	10	718	45	34208	2,56E-08	7,12E-07	LDHB,PDHB,ENO1,OGDH,PDHA1,PFKP,HK2,HKDC1,PFKL,GAPDH
GO:0051693	actin filament capping (BP)	7	718	17	34208	2,82E-08	7,56E-07	CAPZA1,SPTAN1,SPTBN2,CAPZB,CAPZA2,SPTBN1,VIL1
GO:0030048	actin filament-based movement (BP)	7	718	17	34208	2,82E-08	7,56E-07	ACTC1,MYH14,MYH10,MYO1B,MYO6,MYH9,MYO1E

GO:0042274	ribosomal small subunit biogenesis (BP)	6	718	11	34208	3,54E-08	9,31E-07	RPS16,RPS7,RPS15,RPS19,RPS24,RPS6
GO:0050796	regulation of insulin secretion (BP)	12	718	74	34208	4,46E-08	1,15E-06	IQGAP1,SLC25A4,CPT1A,PRKACA,SLC2A1,PRKACB,ITPR2,PRKAR2A,SLC25A6,GNAS,ITPR3,SLC25A5
GO:0006364	rRNA processing (BP)	13	718	90	34208	5,14E-08	1,31E-06	RPS16,RPL5,RPL11,RPL7,RPS7,RPS15,RPL14,RPS19,GEMIN4,RPS24,NOP56,RPL35A,RPS6
GO:0006921	cellular component disassembly involved in apoptosis (BP)	10	718	49	34208	6,12E-08	1,53E-06	KPNB1,PLEC,TJP2,DSP,LMNB1,SPTAN1,TJP1,CTNNB1,LMNA,VIM
GO:0044255	cellular lipid metabolic process (BP)	15	718	128	34208	8,64E-08	2,12E-06	SLC25A1,FHL2,ACADM,CPT1A,FASN,HSD17B12,HADHB,ACSL4,BDH1,TECR,HADHA,FAR1,ACSL3,ELOVL5,ACSL5
GO:0051258	protein polymerization (BP)	8	718	29	34208	1,05E-07	2,55E-06	TUBA1C,TUBA1A,TUBB,TUBA4A,TUBB2B,TUBB2A,TUBB2C,TUBB3
GO:0042493	response to drug (BP)	23	718	301	34208	1,27E-07	3,02E-06	ACTC1,NES,ACADM,CDK1,CPT1A,YWHAZ,LGALS1,BDH1,CTNNB1,HADHA,MSH2,PAM,ATP1A1,CALR,GNAS,XPO1,GGH,MAP1B,MAT2A,ABCD3,LCK,ABCC1,ITGB1
GO:0006006	glucose metabolic process (BP)	13	718	98	34208	1,44E-07	3,38E-06	SLC25A1,CPT1A,ENO1,PRKACA,PRKACB,SLC25A11,SLC25A10,PFKP,GAA,HK2,PFKL,SLC25A13,GAPDH
GO:0019432	triglyceride biosynthetic process (BP)	8	718	33	34208	3,16E-07	7,30E-06	SLC25A1,FASN,HSD17B12,ACSL4,TECR,ACSL3,ELOVL5,ACSL5
GO:0006890	retrograde vesicle-mediated transport, Golgi to ER (BP)	7	718	24	34208	4,42E-07	1,00E-05	TMED10,ARCN1,COPA,COPB1,COPB2,RAB6B,RAB6A
GO:0055085	transmembrane transport (BP)	34	718	630	34208	6,76E-07	1,51E-05	SLC25A1,SLC25A4,ATP2A2,NUP210,PRKACA,SLC2A1,SLC1A5,ATP6V0D1,SLC38A2,SLC3A2,PRKACB,ITPR2,SLC16A1,SLC25A10,PRKAR2A,ATP6V1A,NUP160,SLC25A6,SLC25A22,NUP205,ATP1A1,GNAS,NUP93,ITPR3,HK2,VDAC2,ABCD3,NUP85,TFRC,VDAC1,SLC25A13,SLC25A5,SLC38A1,ABCC1
GO:0016192	vesicle-mediated transport (BP)	17	718	192	34208	7,25E-07	1,60E-05	SEC16A,ARCN1,ARF4,VPS4A,COPA,SPTBN2,AP1B1,AP1G1,PREB,SEC13,CLTC,COPB1,COPB2,MYO5B,AP2B1,RAB6B,RAB6A
GO:0030049	muscle filament sliding (BP)	8	718	37	34208	8,17E-07	1,78E-05	ACTC1,TPM4,MYL6B,TPM1,VIM,MYL6,TPM3,ACTA1
GO:0042273	ribosomal large subunit biogenesis (BP)	5	718	10	34208	9,28E-07	1,99E-05	RPL5,RPL11,RPL7,RPL14,RPL35A
GO:0000086	G2/M transition of mitotic cell cycle (BP)	13	718	115	34208	9,46E-07	2,00E-05	TUBA1A,DYNC1H1,NES,TUBB,TUBA4A,YWHAE,CDK1,HSP90AA1,PRKACA,KHDRBS1,TUBB2C,DYNLL1,PPP2R1A
GO:0051085	chaperone mediated protein folding requiring cofactor (BP)	5	718	11	34208	1,67E-06	3,48E-05	HSPA8,C9orf167,HSPH1,ERO1L,TOR3A
GO:0006094	gluconeogenesis (BP)	8	718	42	34208	2,28E-06	4,68E-05	SLC25A1,ENO1,PRKACA,PRKACB,SLC25A11,SLC25A10,SLC25A13,GAPDH
GO:0051017	actin filament bundle assembly (BP)	6	718	20	34208	2,52E-06	5,12E-05	LIMA1,MYO1B,LCP1,CALD1,ACTN1,ACTN4
GO:0030168	platelet activation (BP)	18	718	234	34208	2,65E-06	5,29E-05	PPIA,TUBA4A,ATP2A2,CFL1,YWHAZ,HSPA5,GAS6,ITPR2,GNB1,CALU,FLNA,GNAS,ITPR3,ACTN1,GNAI2,LCK,ACTN4,GNAI1
GO:0000028	ribosomal small subunit assembly (BP)	4	718	6	34208	2,79E-06	5,51E-05	RPS14,RPS25,RPSA,RPS6

GO:0030036	actin cytoskeleton organization (BP)	13	718	128	34208	3,19E-06	6,22E-05	FLNB,MYH10,CFL1,CAPZA1,DYNLL1,ARPC1A,CORO1C,CAPZB,INF2,CAPZA2,ARPC5,SSH2,CORO1B
GO:0007173	epidermal growth factor receptor signaling pathway (BP)	10	718	76	34208	4,27E-06	8,22E-05	UBB,CDK1,ARF4,UBA52,UBC,CLTC,EPS8,AP2B1,AP2A1,VIL1
GO:0048205	COPI coating of Golgi vesicle (BP)	5	718	13	34208	4,50E-06	8,54E-05	TMED10,ARCN1,COPA,COPB1,COPB2
GO:0000380	alternative nuclear mRNA splicing, via spliceosome (BP)	4	718	7	34208	6,41E-06	1,19E-04	PTBP1,HNRNPM,SFPQ,HNRNPA1
GO:0042026	protein refolding (BP)	4	718	7	34208	6,41E-06	1,19E-04	HSP90AA1,HSPD1,CCT7,HSPA1A
GO:0071377	cellular response to glucagon stimulus (BP)	7	718	37	34208	1,04E-05	1,90E-04	PRKACA,PRKACB,GNB1,PRKAR2A,GNAS,GNB4,GNB2
GO:0009615	response to virus (BP)	13	718	144	34208	1,17E-05	2,11E-04	PSMA2,EEF1G,CFL1,PCBP2,HSPB1,ENO1,CCT5,DNAJC3,ACTA2,BANF1,HNRNPUL1,RPS15A,ABCE1
GO:0019047	provirus integration (BP)	4	718	8	34208	1,26E-05	2,25E-04	XRCC6,PPIA,XRCC5,BANF1
GO:0006662	glycerol ether metabolic process (BP)	5	718	16	34208	1,45E-05	2,56E-04	TMX3,PDIA6,P4HB,PDIA3,PDIA4
GO:0006464	protein modification process (BP)	13	718	150	34208	1,81E-05	3,16E-04	RPN2,PCMT1,UBA52,PCMTD1,UBR5,PCMTD2,ARAF,PAM,HECTD3,HERC1,RPN1,ERO1L,PLOD3
GO:0045454	cell redox homeostasis (BP)	8	718	55	34208	1,85E-05	3,19E-04	TMX3,TXNDC12,PDIA6,P4HB,PDIA3,AIFM1,QSOX2,PDIA4
GO:0034329	cell junction assembly (BP)	10	718	90	34208	1,97E-05	3,36E-04	PLEC,MLLT4,JUP,ACTG1,FLNA,ITGB4,CTNND1,ACTN1,CTNNA1,ITGB1
GO:0006396	RNA processing (BP)	10	718	92	34208	2,39E-05	3,98E-04	HNRNPD,HNRNPK,HNRNPF,DDX17,PABPC4,HNRNPU,PRPF39,HNRNPUL1,HNRNPH1,SYNCRIP
GO:0006916	anti-apoptosis (BP)	15	718	200	34208	2,39E-05	4,02E-04	NPM1,UBB,CDK1,CFL1,HSPB1,UBA52,YWHAZ,HSPA5,UBC,HSPA9,PRDX2,MYO18A,SQSTM1,HSPA1A,HSP90B1
GO:0008645	hexose transport (BP)	7	718	42	34208	2,48E-05	4,09E-04	NUP210,SLC2A1,NUP160,NUP205,NUP93,HK2,NUP85
GO:0006950	response to stress (BP)	13	718	155	34208	2,57E-05	4,19E-04	NPM1,HSPB1,HSP90AA1,HSPA6,HSPD1,HSPA8,HYOU1,HSPH1,HSP90AB1,SQSTM1,HSPA1A,HSP90B1,SERPINH1
GO:0007017	microtubule-based process (BP)	5	718	18	34208	2,74E-05	4,42E-04	TUBA1C,TUBA1A,TUBB,DYNLL2,DYNLL1
GO:0001701	in utero embryonic development (BP)	14	718	179	34208	2,78E-05	4,44E-04	MYH10,RDH10,CSDA,SRSF1,MYH9,CTNNB1,TPM1,MSH2,RCN1,MYO1E,YBX1,PRMT1,PLOD3,ITGB1
GO:0007411	axon guidance (BP)	19	718	307	34208	3,14E-05	4,86E-04	MYH14,MYH10,CDK1,CFL1,HSP90AA1,SPTAN1,MYL12B,SPTBN2,MYH9,ACTG1,CLTC,ABLIM1,MYL6,AP2B1,SPTBN1,TUBB3,HSP90AB1,AP2A1,ITGB1
GO:0006302	double-strand break repair (BP)	8	718	59	34208	3,13E-05	4,88E-04	XRCC6,PRKDC,MSH2,H2AFX,XRCC5,TRIP13,MRE11A,ATM
GO:0006406	mRNA export from nucleus (BP)	8	718	59	34208	3,13E-05	4,88E-04	SRSF3,SRSF7,SRSF1,EIF5A,NUP160,THOC4,DDX39A,UPF1
GO:0055015	ventricular cardiac muscle cell development (BP)	4	718	10	34208	3,65E-05	5,54E-04	FHL2,MYH10,CDK1,LMNA

GO:0006610	ribosomal protein import into nucleus (BP)	3	718	4	34208	3,63E-05	5,55E-04	KPNB1,RPL23,IPO5
GO:0002576	platelet degranulation (BP)	9	718	79	34208	4,18E-05	6,27E-04	PPIA,TUBA4A,CFL1,HSPA5,GAS6,CALU,FLNA,ACTN1,ACTN4
GO:0034332	adherens junction organization (BP)	6	718	33	34208	5,72E-05	8,41E-04	DSP,MLLT4,JUP,ACTG1,CTNND1,CTNNA1
GO:0042059	negative regulation of epidermal growth factor receptor signaling pathway (BP)	6	718	33	34208	5,72E-05	8,41E-04	UBB,UBA52,UBC,CLTC,AP2B1,AP2A1
GO:0006090	pyruvate metabolic process (BP)	5	718	21	34208	6,18E-05	9,01E-04	LDHB,PDHB,SLC16A1,PDHA1,BSG
GO:0060048	cardiac muscle contraction (BP)	5	718	22	34208	7,86E-05	1,13E-03	ACTC1,SRSF1,TPM1,GAA,CAMK2D
GO:0000723	telomere maintenance (BP)	7	718	50	34208	7,95E-05	1,14E-03	PARP1,XRCC6,POLD4,HIST3H3,PRKDC,RFC5,XRCC5
GO:0006511	ubiquitin-dependent protein catabolic process (BP)	12	718	150	34208	8,25E-05	1,17E-03	PSMA2,PSMC2,PSMA7,CUL2,UBR2,UBR5,TCEB1,SKP1,CUL5,SQSTM1,PSMA4,CUL4A
GO:0019059	initiation of viral infection (BP)	4	718	12	34208	8,33E-05	1,17E-03	XRCC6,PPIA,XRCC5,BANF1
GO:0030705	cytoskeleton-dependent intracellular transport (BP)	3	718	5	34208	8,92E-05	1,21E-03	TUBA1C,TUBA1A,TUBB
GO:0071681	cellular response to indole-3-methanol (BP)	3	718	5	34208	8,92E-05	1,21E-03	CTNNB1,JUP,CTNNA1
GO:0071229	cellular response to acid (BP)	3	718	5	34208	8,92E-05	1,21E-03	ANXA2,S100A10,HIST1H2BA
GO:0033119	negative regulation of RNA splicing (BP)	3	718	5	34208	8,92E-05	1,21E-03	RPS26,RPS13,PTBP1
GO:0006754	ATP biosynthetic process (BP)	6	718	37	34208	1,12E-04	1,50E-03	ATP5B,ATP2A2,ATP5C1,ATP1A1,ATP5A1,SLC25A13
GO:0007018	microtubule-based movement (BP)	9	718	90	34208	1,17E-04	1,55E-03	TUBA1C,TUBA1A,DYNC1H1,TUBB,TUBA4A,TUBB2B,TUBB2A,TUBB2C,TUBB3
GO:0016337	cell-cell adhesion (BP)	8	718	74	34208	1,61E-04	2,12E-03	DSP,MYH9,SCRIB,CTNNB1,JUP,CTNND1,CYFIP2,FAT1
GO:0016584	nucleosome positioning (BP)	3	718	6	34208	1,76E-04	2,25E-03	HIST1H1E,HIST1H1C,HIST1H1D
GO:0047497	mitochondrion transport along microtubule (BP)	3	718	6	34208	1,76E-04	2,25E-03	RHOT1,LRPPRC,MAP1B
GO:0051099	positive regulation of binding (BP)	3	718	6	34208	1,76E-04	2,25E-03	ANXA2,S100A10,HIST1H2BA

GO:0006099	tricarboxylic acid cycle (BP)	5	718	26	34208	1,83E-04	2,33E-03	PDHB,OGDH,SUCLA2,DLST,SDHA
GO:0048011	nerve growth factor receptor signaling pathway (BP)	14	718	215	34208	1,99E-04	2,51E-03	YWHAE,UBB,CDK1,PRKACA,UBA52,UBC,PRKACB,ITPR2,CLTC,PRKAR2A,AP2B1,ITPR3,SQSTM1,AP2A1
GO:0009725	response to hormone stimulus (BP)	7	718	58	34208	2,07E-04	2,59E-03	FHL2,ACADM,BDH1,CTNNB1,MMS19,MAT2A,RBM14
GO:0051603	proteolysis involved in cellular protein catabolic process (BP)	5	718	27	34208	2,21E-04	2,74E-03	PSMA2,PSMA7,HSPA5,DNAJC3,PSMA4
GO:0043066	negative regulation of apoptotic process (BP)	16	718	272	34208	2,31E-04	2,84E-03	FHL2,NES,KRT18,HSPB1,CSDA,TMEM161A,HSPA5,DNAJA3,GAS6,HSPD1,CTNNB1,ARAF,CTNNA1,XRCC5,ATM,RPS6
GO:0017148	negative regulation of translation (BP)	5	718	28	34208	2,65E-04	3,20E-03	CAPRIN1,GNB2L1,IGF2BP1,CALR,IGF2BP3
GO:0050690	regulation of defense response to virus by virus (BP)	5	718	28	34208	2,65E-04	3,20E-03	AP1B1,AP1G1,AP2B1,AP2A1,LCK
GO:0015758	glucose transport (BP)	7	718	61	34208	2,84E-04	3,41E-03	NUP210,SLC2A1,NUP160,NUP205,NUP93,HK2,NUP85
GO:0006369	termination of RNA polymerase II transcription (BP)	6	718	44	34208	3,00E-04	3,57E-03	SRSF3,SNRPE,SRSF7,SRSF1,SNRPF,THOC4
GO:0006611	protein export from nucleus (BP)	4	718	17	34208	3,68E-04	4,32E-03	EIF5A,HSPA9,XPO5,CALR
GO:0046688	response to copper ion (BP)	4	718	17	34208	3,68E-04	4,32E-03	ACADM,CDK1,PAM,TFRC
GO:0045905	positive regulation of translational termination (BP)	2	718	2	34208	4,40E-04	5,00E-03	EIF5A2,EIF5A
GO:0006452	translational frameshifting (BP)	2	718	2	34208	4,40E-04	5,00E-03	EIF5A2,EIF5A
GO:0045901	positive regulation of translational elongation (BP)	2	718	2	34208	4,40E-04	5,00E-03	EIF5A2,EIF5A
GO:0010827	regulation of glucose transport (BP)	5	718	31	34208	4,34E-04	5,05E-03	NUP210,NUP160,NUP205,NUP93,NUP85
GO:0006310	DNA recombination (BP)	8	718	86	34208	4,56E-04	5,10E-03	RUVBL2,SMC1A,NONO,RUVBL1,H2AFX,XRCC5,SFPQ,RBM14
GO:0022904	respiratory electron transport chain (BP)	8	718	86	34208	4,56E-04	5,10E-03	ATP5B,UQCRC2,ATP5C1,NDUFA4,ATP5A1,SDHA,ETFA,COX5A
GO:0019433	triglyceride catabolic process (BP)	4	718	18	34208	4,66E-04	5,14E-03	PPP1CA,PRKACA,PRKACB,PPP1CB
GO:0034504	protein localization to nucleus (BP)	4	718	18	34208	4,66E-04	5,14E-03	RPL11,LMNA,CALR,XPO1

GO:0006554	lysine catabolic process (BP)	3	718	8	34208	4,77E-04	5,22E-03	OGDH,DLST,GCDH
GO:0030433	ER-associated protein catabolic process (BP)	5	718	32	34208	5,06E-04	5,50E-03	HSPA5,SEC61B,OS9,HSP90B1,ERLEC1
GO:0006461	protein complex assembly (BP)	9	718	111	34208	5,67E-04	6,12E-03	DARS,CDK1,CAPZA1,SF3B3,PPP2R1A,TCEB2,EPRS,CAPZA2,VIL1
GO:0006486	protein glycosylation (BP)	7	718	70	34208	6,65E-04	7,12E-03	UGGT2,STT3B,STT3A,DDOST,UGGT1,ALG1,RPN1
GO:0006119	oxidative phosphorylation (BP)	3	718	9	34208	7,04E-04	7,49E-03	UQCRC2,ATP5C1,MSH2
GO:0007016	cytoskeletal anchoring at plasma membrane (BP)	3	718	10	34208	9,90E-04	1,04E-02	FLNB,CTNNB1,JUP
GO:0048025	negative regulation of nuclear mRNA splicing, via spliceosome (BP)	3	718	10	34208	9,90E-04	1,04E-02	RBMX,SRSF7,PTBP1
GO:0008283	cell proliferation (BP)	16	718	312	34208	1,02E-03	1,07E-02	SRA1,MYH10,PRDX1,KHDRBS1,CSE1L,GAS6,SCRIB,CDC16,UBR5,GNB1,EPS8,XRCC5,CUL5,RPS27,MRE11A,RPL23A
GO:0034660	ncRNA metabolic process (BP)	4	718	22	34208	1,04E-03	1,08E-02	SNRPE,GEMIN4,SNRPF,SNRPD1
GO:0000289	nuclear-transcribed mRNA poly(A) tail shortening (BP)	4	718	23	34208	1,24E-03	1,27E-02	TNKS1BP1,CNOT1,PABPC1,EIF4A1
GO:0000462	maturation of SSU-rRNA from tricistronic rRNA transcript (SSU-rRNA, 5.8S rRNA, LSU-rRNA) (BP)	2	718	3	34208	1,30E-03	1,28E-02	RPS24,RPS8
GO:0006189	'de novo' IMP biosynthetic process (BP)	2	718	3	34208	1,30E-03	1,28E-02	GART,PAICS
GO:0009991	response to extracellular stimulus (BP)	2	718	3	34208	1,30E-03	1,28E-02	RPS19,ACTA1
GO:0051208	sequestering of calcium ion (BP)	2	718	3	34208	1,30E-03	1,28E-02	CALR,HSP90B1
GO:0003136	negative regulation of heart induction by canonical Wnt receptor signaling pathway (BP)	2	718	3	34208	1,30E-03	1,28E-02	CTNNB1,JUP
GO:0006420	arginyl-tRNA aminoacylation (BP)	2	718	3	34208	1,30E-03	1,28E-02	RARS2,RARS
GO:0019673	GDP-mannose metabolic process (BP)	2	718	3	34208	1,30E-03	1,28E-02	DPM1,GMDS
GO:0048208	COPII vesicle coating (BP)	3	718	11	34208	1,34E-03	1,29E-02	TMED10,PREB,SEC13

GO:0006303	double-strand break repair via nonhomologous end joining (BP)	3	718	11	34208	1,34E-03	1,29E-02	XRCC6,PRKDC,XRCC5
GO:0042149	cellular response to glucose starvation (BP)	3	718	11	34208	1,34E-03	1,29E-02	SLC2A1,HSPA5,MYBBP1A
GO:0006184	GTP catabolic process (BP)	10	718	151	34208	1,38E-03	1,32E-02	ARF4,GNB1,GNAS,EFTUD2,GNAI2,RAB6B,RAB8B,RAB6A,GNAI1,GNB2
GO:0030163	protein catabolic process (BP)	5	718	41	34208	1,61E-03	1,53E-02	PSMC2,PSMC5,CUL2,UBR2,PSMC3
GO:0007050	cell cycle arrest (BP)	9	718	129	34208	1,65E-03	1,56E-02	KHDRBS1,CUL2,GAS2L3,MSH2,CALR,CUL5,ATM,MACF1,CUL4A
GO:0050900	leukocyte migration (BP)	8	718	106	34208	1,80E-03	1,69E-02	PPIA,GAS6,MYH9,SLC3A2,SLC16A1,LCK,BSG,ITGB1
GO:0008629	induction of apoptosis by intracellular signals (BP)	5	718	43	34208	2,00E-03	1,84E-02	DYNLL2,CUL2,DYNLL1,CUL5,CUL4A
GO:0006913	nucleocytoplasmic transport (BP)	4	718	26	34208	1,99E-03	1,84E-02	NPM1,EIF5A,MYBBP1A,NUP205
GO:0000387	spliceosomal snRNP assembly (BP)	4	718	26	34208	1,99E-03	1,84E-02	SNRPE,GEMIN4,SNRPF,SNRPD1
GO:0042776	mitochondrial ATP synthesis coupled proton transport (BP)	3	718	13	34208	2,25E-03	2,05E-02	ATP5B,ATP5C1,ATP5A1
GO:0015986	ATP synthesis coupled proton transport (BP)	3	718	13	34208	2,25E-03	2,05E-02	ATP5B,ATP5C1,ATP5A1
GO:0007569	cell aging (BP)	4	718	27	34208	2,30E-03	2,07E-02	NPM1,CDK1,DNAJA3,HSD17B10
GO:0048678	response to axon injury (BP)	4	718	27	34208	2,30E-03	2,07E-02	CDK1,ARF4,LGALS1,MAP1B
GO:0006564	L-serine biosynthetic process (BP)	2	718	4	34208	2,57E-03	2,18E-02	PHGDH,SHMT2
GO:0015853	adenine transport (BP)	2	718	4	34208	2,57E-03	2,18E-02	SLC25A4,SLC25A5
GO:0051014	actin filament severing (BP)	2	718	4	34208	2,57E-03	2,18E-02	DSTN,VIL1
GO:0045040	protein import into mitochondrial outer membrane (BP)	2	718	4	34208	2,57E-03	2,18E-02	HSP90AA1,SAMM50
GO:0009156	ribonucleoside monophosphate biosynthetic process (BP)	2	718	4	34208	2,57E-03	2,18E-02	PRPS1,PRPS2
GO:0044249	cellular biosynthetic process (BP)	2	718	4	34208	2,57E-03	2,18E-02	PRPS1,PRPS2
GO:0006172	ADP biosynthetic process (BP)	2	718	4	34208	2,57E-03	2,18E-02	ATP5B,ATP5A1
GO:0046796	viral genome transport in host cell (BP)	2	718	4	34208	2,57E-03	2,18E-02	KPNB1,XPO1

GO:0006518	peptide metabolic process (BP)	2	718	4	34208	2,57E-03	2,18E-02	MME,PAM
GO:0008612	peptidyl-lysine modification to hypusine (BP)	2	718	4	34208	2,57E-03	2,18E-02	EIF5A2,EIF5A
GO:0007584	response to nutrient (BP)	7	718	89	34208	2,72E-03	2,29E-02	ACADM,ACSL4,BDH1,ACSL3,GNAI2,ACSL5,TFRC
GO:0042176	regulation of protein catabolic process (BP)	3	718	14	34208	2,82E-03	2,35E-02	XPO1,PSMD2,PSMD3
GO:0009306	protein secretion (BP)	3	718	14	34208	2,82E-03	2,35E-02	GNAS,CANX,PDIA4
GO:0016197	endosome transport (BP)	5	718	47	34208	2,98E-03	2,47E-02	UBB,VPS4A,UBA52,UBC,SQSTM1
GO:0006635	fatty acid beta-oxidation (BP)	4	718	29	34208	3,01E-03	2,48E-02	ACADM,CPT1A,HADHB,HADHA
GO:0051289	protein homotetramerization (BP)	5	718	48	34208	3,26E-03	2,68E-02	ACADM,PFKP,SHMT2,HSD17B10,PFKL
GO:0032480	negative regulation of type I interferon production (BP)	4	718	30	34208	3,42E-03	2,76E-02	UBB,PCBP2,UBA52,UBC
GO:0033077	T cell differentiation in thymus (BP)	4	718	30	34208	3,42E-03	2,76E-02	DNAJA3,PRKDC,CTNNB1,RPS6
GO:0033572	transferrin transport (BP)	4	718	30	34208	3,42E-03	2,76E-02	ATP6V0D1,CLTC,ATP6V1A,TFRC
GO:0045333	cellular respiration (BP)	3	718	15	34208	3,47E-03	2,78E-02	FASTKD3,FASTKD5,SLC25A13
GO:0006919	activation of cysteine-type endopeptidase activity involved in apoptotic process (BP)	6	718	71	34208	3,78E-03	3,01E-02	PDCD6,DNAJA3,HSPD1,AIFM1,RPS3,LCK
GO:0015992	proton transport (BP)	5	718	50	34208	3,90E-03	3,10E-02	ATP5B,ATP6V0D1,ATP5C1,ATP6V1A,ATP5A1
GO:0006366	transcription from RNA polymerase II promoter (BP)	14	718	294	34208	4,00E-03	3,16E-02	PARP1,PSMC5,SRSF3,SNRPE,RBMX,SRSF7,SRSF1,SNRPF,TCEB1,TCEB2,THOC4,YBX1,TRIP13,TRIM29
GO:0055114	oxidation-reduction process (BP)	12	718	233	34208	4,02E-03	3,16E-02	ACADM,HADHB,OGDH,PHGDH,PDHA1,PAM,FAR1,DHCR7,SDHA,ERO1L,QSOX2,ETFA
GO:0030240	skeletal muscle thin filament assembly (BP)	2	718	5	34208	4,22E-03	3,18E-02	ACTC1,ACTA1
GO:0051096	positive regulation of helicase activity (BP)	2	718	5	34208	4,22E-03	3,18E-02	SSBP1,MSH2
GO:0005981	regulation of glycogen catabolic process (BP)	2	718	5	34208	4,22E-03	3,18E-02	PPP1CA,PPP1CB
GO:0009113	purine base biosynthetic process (BP)	2	718	5	34208	4,22E-03	3,18E-02	GART,PAICS
GO:0050847	progesterone receptor signaling pathway (BP)	2	718	5	34208	4,22E-03	3,18E-02	PHB,UBR5

GO:0002026	regulation of the force of heart contraction (BP)	3	718	16	34208	4,20E-03	3,25E-02	ATP2A2,ATP1A1,GAA
GO:0034199	activation of protein kinase A activity (BP)	3	718	16	34208	4,20E-03	3,25E-02	PRKACA,PRKACB,PRKAR2A
GO:0006509	membrane protein ectodomain proteolysis (BP)	3	718	16	34208	4,20E-03	3,25E-02	RBMX,MYH9,ERAP1
GO:0015991	ATP hydrolysis coupled proton transport (BP)	4	718	32	34208	4,34E-03	3,25E-02	ATP5B,ATP6V0D1,ATP6V1A,ATP5A1
GO:0008284	positive regulation of cell proliferation (BP)	16	718	361	34208	4,38E-03	3,27E-02	EPCAM,LRP5,RPS9,RPS4X,EIF5A2,DNAJA2,CNBP,EIF5A,NAP1L1,CALR,GNAI2,SHMT2,SSR1,TNS3,RPS15A,ITGB1
GO:0043065	positive regulation of apoptotic process (BP)	10	718	178	34208	4,55E-03	3,38E-02	PDIA3,DNAJA3,HSPD1,PRKDC,GNB2L1,SCRIB,CTNNB1,ATM,RPS6,ITGB1
GO:0007420	brain development (BP)	10	718	179	34208	4,74E-03	3,50E-02	NES,MYH10,ARF4,ATP6V0D1,PRKDC,BDH1,GPR56,PHGDH,ACSL3,ATM
GO:0031124	mRNA 3'-end processing (BP)	4	718	33	34208	4,86E-03	3,57E-02	SRSF3,SRSF7,SRSF1,THOC4
GO:0000381	regulation of alternative nuclear mRNA splicing, via spliceosome (BP)	3	718	17	34208	5,03E-03	3,63E-02	RBMX,PTBP1,DDX5
GO:0006260	DNA replication (BP)	9	718	152	34208	4,96E-03	3,63E-02	POLD4,PHB,CDK1,SSBP1,RFC5,NAP1L1,UPF1,RBM14,MCM3
GO:0031100	organ regeneration (BP)	5	718	53	34208	5,02E-03	3,64E-02	CDK1,CSDA,LCP1,EIF4A1,CAD
GO:0050821	protein stabilization (BP)	5	718	53	34208	5,02E-03	3,64E-02	DNAJA3,HSPD1,FLNA,CALR,GAPDH
GO:0008219	cell death (BP)	9	718	156	34208	5,86E-03	4,20E-02	GARS,HSPB1,SPTBN2,DNAJA3,HSPD1,NOP56,FUS,ATM,ATXN10
GO:0006401	RNA catabolic process (BP)	3	718	18	34208	5,94E-03	4,22E-02	HNRNPD,PABPC4,ABCE1
GO:0006164	purine nucleotide biosynthetic process (BP)	3	718	18	34208	5,94E-03	4,22E-02	GART,PRPS1,PAICS
GO:0070588	calcium ion transmembrane transport (BP)	4	718	35	34208	6,01E-03	4,23E-02	ATP2A2,ITPR2,ITPR3,CUL5
GO:0006606	protein import into nucleus (BP)	4	718	35	34208	6,01E-03	4,23E-02	KPNB1,IPO7,PDIA3,IPO9
GO:0014075	response to amine stimulus (BP)	2	718	6	34208	6,24E-03	4,23E-02	CDK1,CAD
GO:0051271	negative regulation of cellular component	2	718	6	34208	6,24E-03	4,23E-02	ACTN1,ACTN4

	movement (BP)							
GO:0006924	activation-induced cell death of T cells (BP)	2	718	6	34208	6,24E-03	4,23E-02	DNAJA3,RPS6
GO:0034501	protein localization to kinetochore (BP)	2	718	6	34208	6,24E-03	4,23E-02	CDK1,ZNF828
GO:0007270	neuron-neuron synaptic transmission (BP)	2	718	6	34208	6,24E-03	4,23E-02	TMOD2,VDAC1
GO:0030388	fructose 1,6-bisphosphate metabolic process (BP)	2	718	6	34208	6,24E-03	4,23E-02	PFKP,PFKL
GO:0071901	negative regulation of protein serine/threonine kinase activity (BP)	2	718	6	34208	6,24E-03	4,23E-02	LRP5,HSPB1
GO:0007098	centrosome cycle (BP)	2	718	6	34208	6,24E-03	4,23E-02	NPM1,CDK1
GO:0006091	generation of precursor metabolites and energy (BP)	5	718	56	34208	6,35E-03	4,29E-02	SLC25A3,ATP5B,SLC25A4,OGDH,DLST
GO:0007067	mitosis (BP)	10	718	187	34208	6,40E-03	4,30E-02	TUBB,CDK1,TUBB2B,TUBB2A,CDC16,RUVBL1,CLTC,AKAP8,PPP1R12A,RPS6
GO:0006833	water transport (BP)	4	718	36	34208	6,65E-03	4,46E-02	PRKACA,PRKACB,PRKAR2A,GNAS
GO:0016477	cell migration (BP)	7	718	105	34208	6,74E-03	4,48E-02	CDK1,SCRIB,JUP,MYO18A,FAT1,TNS3,ITGB1
GO:0006470	protein dephosphorylation (BP)	7	718	105	34208	6,74E-03	4,48E-02	PPP1CA,PPP6C,PPP2R1A,TIMM50,SSH2,PPP1CB,PPP1R12A
GO:0045214	sarcomere organization (BP)	3	718	19	34208	6,94E-03	4,55E-02	TPM1,ACTG1,ITGB1
GO:0055007	cardiac muscle cell differentiation (BP)	3	718	19	34208	6,94E-03	4,55E-02	ACADM,CALR,ITGB1
GO:0046686	response to cadmium ion (BP)	3	718	19	34208	6,94E-03	4,55E-02	CDK1,BDH1,CTNNB1
GO:0045216	cell-cell junction organization (BP)	5	718	58	34208	7,36E-03	4,80E-02	MLLT4,JUP,ACTG1,CTNND1,CTNNA1
